# Associations between body composition, fat distribution and metabolic consequences of excess adiposity with severe COVID-19 outcomes: observational study and Mendelian randomisation analysis

**DOI:** 10.1038/s41366-021-01054-3

**Published:** 2022-01-14

**Authors:** Min Gao, Qin Wang, Carmen Piernas, Nerys M. Astbury, Susan A. Jebb, Michael V. Holmes, Paul Aveyard

**Affiliations:** 1 Nuffield Department of Primary Care Health Sciences, University of Oxford, Radcliffe Observatory Quarter, Oxford, UK; 2 NIHR Oxford Biomedical Research Centre, Oxford University Hospitals, NHS Foundation Trust, Oxford, UK; 3 Nuffield Department of Population Health, University of Oxford, Old Road Campus, Oxford, UK; 4 Medical Research Council Population Health Research Unit, University of Oxford, Oxford, UK

**Keywords:** Microbiology, Genetics

## Abstract

**Background:**

Higher body mass index (BMI) and metabolic consequences of excess weight are associated with increased risk of severe COVID-19, though their mediating pathway is unclear.

**Methods:**

A prospective cohort study included 435,504 UK Biobank participants. A two-sample Mendelian randomisation (MR) study used the COVID-19 Host Genetics Initiative in 1.6 million participants. We examined associations of total adiposity, body composition, fat distribution and metabolic consequences of excess weight, particularly type 2 diabetes, with incidence and severity of COVID-19, assessed by test positivity, hospital admission, intensive care unit (ICU) admission and death.

**Results:**

BMI and body fat were associated with COVID-19 in the observational and MR analyses but muscle mass was not. The observational study suggested the association with central fat distribution was stronger than for BMI, but there was little evidence from the MR analyses than this was causal. There was evidence that strong associations of metabolic consequences with COVID-19 outcomes in observational but not MR analyses. Type 2 diabetes was strongly associated with COVID-19 in observational but not MR analyses. In adjusted models, the observational analysis showed that the association of BMI with COVID-19 diminished, while central fat distribution and metabolic consequences of excess weight remained strongly associated. In contrast, MR showed the reverse, with only BMI retaining a direct effect on COVID-19.

**Conclusions:**

Excess total adiposity is probably casually associated with severe COVID-19. Mendelian randomisation data do not support causality for the observed associations of central fat distribution or metabolic consequences of excess adiposity with COVID-19.

## Background

Higher body mass index (BMI) is associated with severe outcomes from COVID-19, after adjusting for common diseases caused by excess weight [1]. There is also evidence that higher BMI may have a bigger impact in younger people and people of non-White ethnic groups [2–4]. It is possible that the effects of higher body mass are compounded by differences in body composition (such as the ratio of muscle to fat) or the distribution of adiposity, where central distribution is associated with a higher likelihood of metabolic disturbance at any given BMI. A more detailed understanding of the relationship between body size and composition may provide insights to the mechanisms linking obesity and severe COVID-19 outcomes.

SARS-CoV-2 viral entry via angiotensin converting enzyme-2 receptors on alveolar type 2 pneumocytes is followed by viral proliferation and cytotoxicity, marked local complement deposition, severe inflammation in the alveoli and frequent and widespread microthromboses in the pulmonary capillaries [5]. Obesity may exacerbate these processes. Obesity is associated with increased circulating pro-inflammatory cytokines and key complement components, dysfunction of both endothelium and platelets, reduced serum adiponectin, and reduced fibrinolytic capacity, all of which could exacerbate the pulmonary pathology of COVID-19 [6, 7]. These are consequences of the metabolic dysfunction that frequently accompanies obesity. Metabolic dysfunction in obesity relates both to excess intracellular lipid in cells other than adipocytes and insulin resistance leading to compensatory hyperinsulinemia [8].

It is also possible that the association between excess weight and severe outcomes of COVID-19 arises due to residual confounding. Obesity is associated with an increased risk of cardiometabolic diseases, which themselves have also been associated with severe COVID-19 [9], however it is unclear whether obesity is an independent determinant of COVID-19 severity. Incomplete measurement of comorbidity and failure to capture all comorbidity may leave an apparent independent association [10]. Mendelian randomisation (MR) can overcome this using unconfounded genetic variation as a natural experiment to investigate the causal relations between risk factors and outcomes in observational data [11]. The aim here is to use observational and MR analyses to examine associations between total adiposity, body composition, fat distribution, metabolic consequences of excess adiposity, particularly type 2 diabetes, with incidence and severity of COVID-19.

## Methods

### Study design and data sources

For the observational study, we used the UK Biobank (UKBB) prospective cohort that recruited 502,664 participants aged 40–69 years between 2006 and 2010 [12, 13], and ethical approval was obtained from the North West Multicentre Research Ethics Committee (11/NW/03820). Participants reported on sociodemographic, physical, behavioural, and health-related factors at baseline. Trained staff measured weight, body composition using a Tanita BC418MA bioimpedance (BIA) analyser, height, and waist and hip circumference, and took samples for biological and genetic analyses [14, 15].

Data from UKBB were linked to COVID-19 test results (Public Health England) between March 16 and November 10, 2020, in England only; and linked data on hospital records up to June 30, 2020 and death records up to September 19, 2020. More information on COVID-19 in UKBB can be found here: http://biobank.ndph.ox.ac.uk/ukb/exinfo.cgi?src=COVID19_tests. The cohort was followed up until the earliest occurrence of a COVID-19 outcome of interest, death from other causes, or the study end date (November 10 2020).

Two sample MR analyses were used to assess the causal effects of adiposity on the risk of COVID-19 outcomes. Data on adiposity, lean mass, fat distribution and metabolic biomarkers are publicly available (Supplementary, Table S1). Data on COVID-19 endpoints were obtained from GWAS summary statistics reported by the COVID-19 Host Genetics Initiative, release 5, Jan 2021 (https://www.covid19hg.org) (Supplementary Table S1) [16].

### Outcomes

The observational study included the following outcomes: (1) COVID-19 test positivity, defined as positivity with COVID-19 by polymerase chain reaction; (2) COVID-19 hospital admission, defined as having ICD-10 code in hospital record for either confirmed (U07.1) or suspected COVID-19 (U07.2); (3) COVID-19 intensive care unit (ICU) admission, defined as critical care admissions to COVID-19; (4) COVID-19 death, defined as individuals who had died with COVID-19. In the MR analysis, outcomes included: (1) COVID-19 test; (2) COVID-19 hospital admission; (3) Very severe confirmed COVID-19 defined as hospitalised COVID-19 cases with respiratory support or death.

### Exposures

The exposures related to total adiposity, body composition, fat distribution and metabolic consequences of excess adiposity but measured differently in the observational and MR studies because of the constraints of the source data. Fat in the peripheral adipose tissue is metabolically inert [17], but truncal fat is associated with metabolic consequences of excess adiposity leading to organ dysfunction, metabolic disturbance, dysregulated secretion of cytokines and adipokines, and insulin resistance [18].

In the UKBB observational study, the exposures were:

1. Total adiposity assessed by body mass index (BMI) and whole-body fat mass index (FMI, calculated as fat mass divided by height squared) assessed by bioelectrical impedance analysis (BIA).
2. Lean mass index assessed by BIA via appendicular skeletal muscle mass index (SMMI), calculated as the sum of the predicted muscle mass from the four limbs divided by height squared.
3. Fat distribution assessed by waist-hip circumference ratio (WHR).
4. Metabolic disturbance was defined as the metabolic consequences of excess adiposity, marked by the presence of non-diabetic hyperglycaemia, type 2 diabetes, or non-alcoholic fatty liver disease (NAFLD) [19, 20]. These were reported doctor diagnoses, or from medication, or HbA1c > 6%, or coded in the hospital in-patient data according to the International Classification of Diseases 10th revision (ICD-10) E11.0-E11.9. NAFLD was defined as ICD-10 code K76.0.
5. Type 2 diabetes, a condition typically due to insulin resistance and beta-cell failure [21].

In the MR analyses, we developed genetic instruments using public GWAS summary data via clumping [22]. The clumping method is described on the homepage of PLINK (https://zzz.bwh.harvard.edu/plink/). For each exposure, clumps were formed around ‘index variants’ that had *p* values less than 1x5e-8. Index variants were chosen starting with the lowest *p* value. Secondary hits were identified if they were within the clumping window (10 Mb) of an index SNP, reached GWAS significance (*p* < 5e-8) and had a low LD with the index SNP (*r*^2^ < 0.001 based on 1000 Genomes phase 3 data from European descendants). Genetic instruments were developed for:

1. Total adiposity assessed by BMI, and body fat percentage.
2. Lean mass assessed by whole-body fat-free mass, and arm and leg lean mass.
3. Fat mass distribution assessed by peripheral fat mass (arm and leg fat mass, hip circumference), and abdominal adiposity (trunk fat, waist circumference, hip circumference, WHR and waist-to-hip ratio adjusted for BMI [WHRadj]).
4. Metabolic consequences of excess adiposity assessed by genetic markers for insulin resistance, insulin-like growth factor (IGF-1) [23], glucose [24], glycated haemoglobin [24], and adiponectin [25]. We also assessed lipid parameters that relate to metabolic disturbance, including apolipoprotein A, high-density lipoproteins (HDL), apolipoprotein B, low-density lipoprotein (LDL), triglycerides [26].
5. Type 2 diabetes.

In the observational analyses, where exposures were continuous e.g. BMI, we calculated sex-specific z-scores. This created a standard unit of exposure for continuous variables. For quantitative traits in MR analyses, rank-based inverse normal transformation (RINT) is generally applied to achieve a normal distribution, during which the residuals of the raw values (after adjusting for the covariates) were mapped to the quantiles of a normal distribution. The values after RINT are in a standard unit and are used in the GWAS analyses.

### Covariates for observational study

Covariates were binary unless otherwise specified. Ethnicity was classified as white, black, Asian, other or missing. Other covariates included Townsend index of deprivation (quintiles); education group (four categories); smoking status (never, previous, current, missing); physical activity derived from metabolic equivalent of task scores per week (low, moderate, high); alcohol intake (none, occasional, moderate, heavy, missing); fruit and vegetables intake (servings/day); non-obesity-related morbidity (namely, COPD, asthma, autoimmune condition, colitis, Crohn disease) and obesity-related morbidity (namely, hypertension, CVD, Reflux, sleep apnoea), with each disease treated as a binary variable. For details and distributions on these covariates, see Supplementary Table S2.

### Statistical analyses

Participants in the observational UKBB study were excluded if they were living outside England and so without data on COVID-19 data (*n* = 56,649, 11.3%), died before the COVID-19 pandemic (set as February 1, 2020, *n* = 29,477, 5.9%), or with missing data for the main exposures (*n* = 10,352, 2.1%). We used directed acyclic graph to identify potential confounders, colliders, and biasing relationships (Supplementary Fig. S1). Confounding would be maximally reduced by controlling for demographic factors (age, gender, Townsend index, education) as well as behavioural risk factors (smoking, alcohol consumption, diet, and physical activity). Since behavioural risk factors were incompletely measured, we further adjusted for downstream morbidity which would increase the severity of COVID-19, that is, non-obesity-related morbidity and obesity-related morbidity.

In the observational study, we examined the association of each exposure separately with COVID-19 outcomes. To do so, we used Cox proportional hazards models with follow up time (days) as timescale variable to obtain hazard ratios with 95% confidence intervals (CIs) with sequential adjustment. The adjustments were for age and gender (Model 1), other demographic factors (Model 2), behavioural risk factors (Model 3), non-obesity-related morbidity (Model 4), and obesity-related morbidity (Model 5, final model). Since fat mass and muscle mass are strongly correlated, we used mutually adjusted models for these exposures. The proportional hazards assumption was based on Schoenfeld residuals and was not violated. We corrected for regression dilution bias using the MacMahon-Peto method, using data for each exposure collected in three subsequent re-surveys in 2012–2013; 2014, and 2019 [27, 28]. In sensitivity analysis, we assessed the impact of potential selection bias by weighting for demographic factors that predicted participation in UK Biobank [29]. Restricted cubic spline models with the same covariate specification were computed with five knots to examine whether the association was non-linear, testing departures from linearity with likelihood ratio tests [30]. Multiplicative interaction terms were fitted in final models to examine heterogeneity in the associations by age, gender, ethnic group, hypertension and CVD.

The MR analyses were conducted via inverse-variance weighted MR analysis and sensitivity analyses included weighted median, MR-Egger, and weighted mode [31]. Different methods have different assumptions and limitations, thus when all the methods give consistent estimates, we have more confidence in the causal estimates. The MR estimates are reported as log(OR) in COVID-19 per SD higher in quantitative exposure traits, or equivalently log(OR) in COVID-19 per log(OR) in binary exposure traits. For each exposure, genetic instruments were identified by clumping (as stated in the method section). Then, same set of SNPs were identified in the outcome GWAS. If the SNPs were missing in the outcome GWAS, proxies (*R*^2^ > 0.8) were used. The harmonisation of SNP-exposure and SNP-outcome associations were run by TwoSampleMR:harmonise_data function, to ensure both associations were flipped to the same allele. The MR results across all the methods (including number of SNPs used) are provided in Supplementary material 2 and Supplementary material 3. We also conducted multivariate modelling to assess the comparative causal role of exposure traits for the risk of COVID-19 guided by conditional F-statistics [32, 33].

To assess whether the association between adiposity and COVID-19 is related principally to total adiposity, its distribution, or metabolic disturbance, we added the WHR and metabolic disturbance to the model including BMI in both observational study and multivariate MR models.

## Results

Of 435,504 participants in the observational study during the study period (1st Feb 2020–10th November 2020), 5,566 tested positive for COVID-19 prior to 10th November 2020, of whom 567 were admitted to hospital with COVID-19, 107 were admitted to ICU, and 366 died of COVID-19 (accounting for 26% of hospital and 43% of ICU admission). Forty-three percent of deaths occurred in people who had been in ICU, and 26% occurred in people who had been in hospital but not ICU, meaning these outcomes were not independent. The mean age was 68 years, mean BMI was 27.4 kg/m^2^ (standard deviation (SD) 4.8), with 12.7% having conditions related to the metabolic disturbances of excess adiposity and 9.2% having type 2 diabetes (Supplementary Table S2).

In the MR analyses, genetic associations with the outcomes were obtained from release 5 (January 2021) of the COVID-19 host genetics initiative analysed for people of European descent. 38,984 tested positive for COVID-19 among 1,683,768 people, 9986 were hospitalised with COVD-19 among 1,887,658, and 5101 received ventilator support or died with COVID-19 among 1,388,342 (Supplementary material 1).

### Association between total adiposity and COVID-19 outcomes

In the observational study, each SD higher BMI was associated with 22% increased risk in COVID-19 test positivity adjusted for non-obesity-related comorbidity, and 14% increase when adjusted for obesity-related comorbidity (Supplementary Table S3a). The association between BMI and severe COVID-19 was stronger, with the main attenuation of risk occurring from adjustment for obesity-related comorbidity. The strength of associations between FMI and COVID-19 outcomes was similar to that of BMI (Table 1). Consistent estimates from final models were obtained with inverse probability weighting (Supplementary Table S4).

**Table 1:** Associations of adiposity markers with COVID-19 outcomes in the UK Biobank study (*n* = 435,504).

	COVID-19 positive test (*N* = 5566)	COVID-19 hospital admission (*N* = 567)	COVID-19 ICU admission (*N* = 107)	COVID-19 death (*N* = 366)
**Exposures**	HR (95% CI)	HR (95% CI)	HR (95% CI)	HR (95% CI)
BMI	1.14(1.09–1.20)	1.21(1.11–1.31)	1.38(1.16–1.65)	1.23(1.11–1.37)
FMI	1.11(1.01–1.21)	1.21(1.05–1.40)	1.04(0.76–1.43)	1.21(1.02–1.45)
SMMI	1.05(0.96–1.13)	1.01(0.89–1.15)	1.32(0.99–1.76)	1.03(0.88–1.22)
WHR	1.21(1.12–1.31)	1.36(1.20–1.55)	1.43(1.07–1.92)	1.37(1.17–1.61)
Metabolic disturbance	1.50(1.32–1.69)	1.69(1.39–2.04)	1.54(0.99–2.41)	1.77(1.41–2.22)
Type 2 diabetes	1.51(1.33–1.72)	1.51(1.23–1.85)	1.24(0.76–2.02)	1.80(1.42–2.28)

Estimates are hazard ratios (HR) with 95% CI per one increase in *z* score of the exposure except for metabolic disturbance and type 2 diabetes which was binary exposures.

Adjusted for demographic factors (age, gender, Townsend index, education), behavioural risk factors (smoking, alcohol consumption, diet and physical activity), non-obesity-related morbidity (COPD, asthma, autoimmune rheumatological conditions, ulcerative colitis and Crohn’s disease) and obesity-related morbidity (hypertension, CVD, GORD and sleep apnoea).

The spline analyses and likelihood ratio tests produced evidence that the associations of BMI, FMI with COVID-19 test positivity and death were linear (*P* > 0.05), while the splines for the associations with hospital admission were non-linear (*P* < 0.05) (Supplementary Fig. S2a,b).

**Fig. 1:**
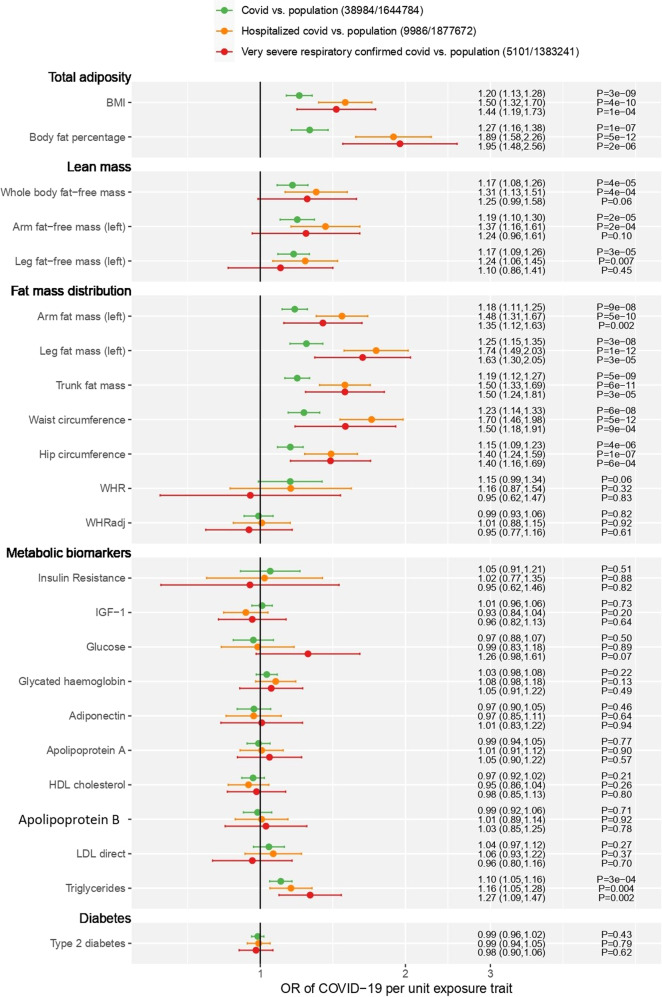
The MR association between adiposity traits and COVID-19 outcomes. Note: definitions of cases and controls for COVID-19 GWAS data are in Supplementary material 1.

In the MR analyses, each SD higher BMI was associated with 20% increased odds of COVID-19 test positivity and more than 50% increased odds of severe COVID-19 (Fig. 1). The associations with the proportion of body fat were similar. The results were consistent across different MR methods (Supplementary Fig. S3).

### Association between lean mass and COVID-19 outcomes

There was no evidence in the observational study that SMMI was associated with any COVID-19 outcomes (Table 1 and Supplementary, Table S3c). However, in the MR analyses, arm and leg fat-free mass and whole-body fat-free mass were associated with increased odds of COVID-19 test positivity and severe COVID-19, with the odds being between 17% and 37% higher per SD (Fig. 1).

### Association between fat distribution and COVID-19 outcomes

The observational associations between WHR and COVID-19 outcomes were stronger than for measures of total adiposity (Table 1). As with total adiposity measures, the associations of WHR with COVID-19 test positivity, hospitalisation and death were linear (*P* > 0.05, Supplementary Fig. S2).

However, in MR analyses, there was weak evidence that WHR was associated with COVID-19 outcomes (Fig. 1) but stronger associations for trunk fat and waist circumference were observed. Trunk fat and waist circumference were associated with around 20% increased odds of COVID-19 test positivity and 50-70% increased odds of severe COVID-19.

### Association between metabolic consequences of excess adiposity and COVID-19 outcomes

The metabolic consequences of excess adiposity were strongly associated with COVID-19 test positivity and severe COVID-19, with risks around 50% higher (Table 1). However, the MR analyses provided no evidence to support the causal role of the metabolic disturbance arising from excess adiposity. There was no evidence of association between biomarkers typically indicating insulin resistance and COVID-19 test positivity or severe disease (Fig. 1). There was a positive association for triglycerides with COVID-19 but no other lipid measures.

### Association between type 2 diabetes and COVID-19 outcomes

In the observational study, type 2 diabetes was associated with a 51% increased risk of COVID-19 positivity and a 50-80% increased risk of severe disease (Table 1). In the MR analysis, there was no evidence that type 2 diabetes increased the risk of any COVID-19 outcome (Fig. 1).

#### Modification of the associations between adiposity markers and COVID-19 outcomes by age, gender, ethnicity and CVD

In the observational analyses, there was little evidence that age or gender modified the association between BMI and COVID-19, but some evidence that ethnicity did so (Supplementary Fig. S4a–e).

#### Assessing the risks from total adiposity mutually adjusting for fat distribution, metabolic consequences of excess adiposity and type 2 diabetes

In the observational analyses adjusted for all covariates (final models), the coefficients for BMI reduced by over 30% when further adjusting for fat distribution or metabolic disturbance, while those for metabolic consequences of excess adiposity reduced by less than 20% when mutually adjusting for BMI (Table 2).

**Table 2:** Observational associations of BMI and metabolic consequences of excess adiposity (mutually adjusted), BMI and type 2 diabetes (mutually adjusted), and BMI and WHR (mutually adjusted) with COVID-19 outcomes.

	COVID-19 positive test (*N* = 5566)	COVID-19 hospital admission (*N* = 567)	COVID-19 ICU admission (*N* = 107)	COVID-19 death (*N* = 366)
Exposures	HR (95% CI)	HR (95% CI)	HR (95% CI)	HR (95% CI)
BMI (unadjusted for WHR/metabolic disturbance/type 2 diabetes)	1.14(1.09–1.20)	1.21(1.11–1.31)	1.38(1.16–1.65)	1.23(1.11–1.37)
BMI (adjusted for WHR)	1.10(1.04–1.16)	1.13(1.03–1.24)	1.02(0.62–1.69)	1.16(1.03–1.30)
BMI (adjusted for metabolic disturbance)	1.11(1.05–1.17)	1.16(1.06–1.26)	1.35(1.13–1.62)	1.17(1.05–1.30)
BMI (adjusted for type 2 diabetes)	1.11(1.05–1.17)	1.17(1.08–1.28)	1.38(1.15–1.65)	1.17(1.05–1.31)
Waist to hip ratio (unadjusted for BMI)	1.21(1.12–1.31)	1.36(1.20–1.55)	1.43(1.07–1.92)	1.37(1.17–1.61)
Waist to hip ratio (adjusted for BMI)	1.14(1.04–1.24)	1.25(1.09–1.45)	1.17(0.84–1.63)	1.24(1.04–1.49)
Metabolic disturbance (unadjusted for BMI)	1.50(1.32–1.69)	1.69(1.39–2.04)	1.54(0.99–2.41)	1.77(1.41–2.22)
Metabolic disturbance (adjusted for BMI)	1.42(1.25–1.61)	1.56(1.29–1.90)	1.31(0.83–2.07)	1.64(1.29–2.07)
Type 2 diabetes (unadjusted for BMI)	1.51(1.33–1.72)	1.51(1.23–1.85)	1.24(0.76–2.02)	1.80(1.42–2.28)
Type 2 diabetes (adjusted for BMI)	1.42(1.24–1.63)	1.38(1.11–1.70)	1.31(0.83–2.07)	1.65(1.29–2.11)

Estimates are unweighted hazard ratios (HR) with 95% CI per one unit increase in z score of the exposure; except for conditions arising from the metabolic consequences of excess adiposity and type 2 diabetes, which are binary exposures and adjusted for social and demographic factors, behavioural risk factors and comorbidity.

**Fig. 2:**
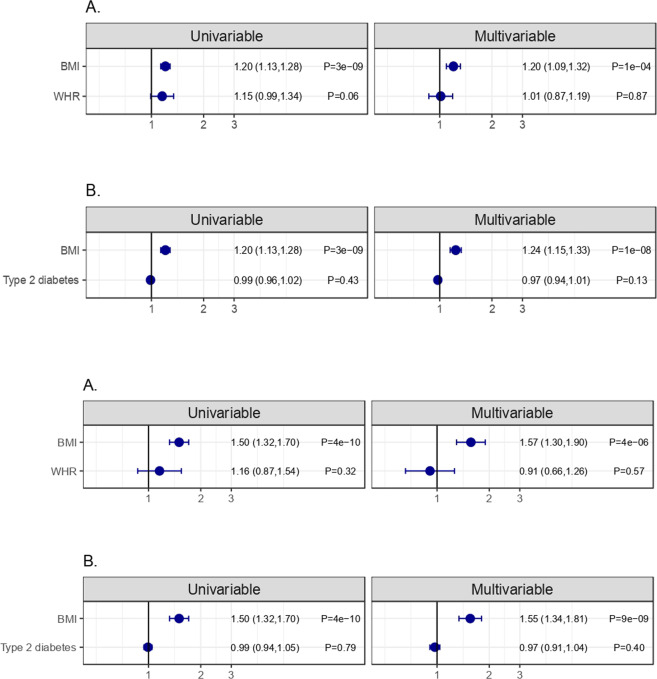
Univariable and multivariable MR associations of BMI, WHR, type 2 diabetes with COVID-19 outcomes. MR associations of **A** BMI and WHR (univariable and multivariable) and **B** BMI and type 2 diabetes (univariable and multivariable) with COVID-19 test (1) and hospital admission (2) in multivariable MR. Note: For quantitative traits, the units are OR per SD; for binary traits, the units are OR per log(OR).

However, the multivariable MR analysis produced contrasting findings from the observational study (Fig. 2). Adjusting for waist-hip ratio, type 2 diabetes, or metabolic disturbance did not meaningfully alter the relationship between BMI and COVID-19 outcomes (Fig. 2 and Supplementary Fig. S5). As in the univariable MR analyses, there was no evidence that WHR or type 2 diabetes were themselves associated with COVID-19 test positivity or severe COVID–19. This same pattern was seen when in multivariable MR with all the traits related to central fat distribution, or metabolic disturbance (Supplementary Fig. S6).

## Discussion

In the observational study, total adiposity measured by BMI and FMI was significantly associated with COVID-19 positivity, hospitalisation, ICU admission (BMI only) and death, driven by stronger associations for people with a BMI above the mean. MR analyses showed consistent associations with similar effect estimates for total adiposity. However, the observational study and MR analyses produced discordant findings on fat distribution. The observational study suggested that the association between central fat distribution and COVID-19 outcomes was stronger than for total adiposity. Also, the observational study showed the strongest associations with metabolic consequences of excess adiposity, namely insulin resistance and type 2 diabetes. The MR analyses however found less compelling evidence that central fat distribution, insulin resistance or other markers of metabolic disturbance from excess adiposity were casually associated with COVID-19 outcomes.

In our study, some cases of COVID-19 derived from a time when testing for COVID-19 was occurring mainly in people ill enough to be medically evaluated, clouding data on incidence. As such, the study mainly examines associations in people with more severe COVID-19. The UK Biobank employed extensive and rigorous assessment of its participants, allowing adjustment for multiple confounders, but the key exposures were assessed 10–14 years ago. We corrected for regression dilution bias, but this could still bias estimates of association towards the null. MR analyses are not influenced by regression dilution bias and showed comparable risk from BMI. Also, MR analyses used genetic variants to proxy the exposures, which through meiosis are generally free of residual confounding. The strong *F*-statistics (*F* > 10, Supplementary Fig. S6) in the univariable MR analyses suggests that weak instrument bias should be minimal [34]. In addition, similar results were observed across the four MR methods, where each has different assumptions and limitations, suggesting that MR findings were not affected by unbalanced horizontal pleiotropy [35]. The non-randomness of study participation and COVID-19 testing may lead to collider bias [36], however, the use of genetic data meta-analysed across multiple populations and cohorts with different study designs and sampling strategies lowers this risk.

Our observational study and MR analyses suggest that excess total adiposity increases susceptibility to infection and severe COVID-19, which is supported by a recent review from large studies [37]. There was no evidence of association between SMMI and admission to hospital or death, suggesting that the links to BMI are driven by excess adiposity. Some evidence has suggested that abdominal fat could confer additional risks for COVID-19 [37–39] and a few studies have reported that visceral obesity measured by computed tomography could be an important independent risk factor, superior to BMI in predicting the severe COVID-19 [37, 38, 40]. Our observational analyses found that the association between WHR and COVID-19 outcomes was stronger than for BMI in individuals with or without obesity, supporting previous research. However, our MR study and other MR studies did not support this and indicated that the impact of WHR on COVID-19 was weaker and disappeared after adjustment for BMI [41]. Prospective associations of visceral fat distribution with COVID-19 outcomes may be partly due to the clinical clustering of metabolic risk factors with obesity. As such, the impact of visceral fat accumulation observed may not be causal, but may have arisen because of other concomitant factors that predict likelihood of receiving specialist care for COVID-19.

The clearest finding from our observational study was that conditions strongly associated with insulin resistance, a metabolic consequence of excess adiposity, were strongly associated with risk of severe COVID-19 and adjusting for total adiposity did not greatly diminish the strength of this relationship. The MR analyses, however, showed strong evidence that genetic markers of type 2 diabetes and glucose dysregulation were at most weakly associated with COVID-19 outcomes. These null associations were not due to weak instrument bias, with *F*-statistics > 10 for all these traits. It could be explained if glucose dysregulation in type 2 diabetes is not the factor that explains higher risk for severe COVID-19 in people with type 2 diabetes. One mechanism might be inflammation, with recent findings from Mendelian randomisation studies and two randomised controlled trials converging on IL6R inhibition as an effective therapeutic approach for COVID-19 [42–45]. The hypothesis that this is and inflammatory and not a glucose effect is not supported by findings from cohort studies showing that type 1 diabetes is also a risk factor for severe outcomes adjusted for cardiovascular disease and that risk is proportional to HbA1c [46]. It is possible that part of the risk in type 1 diabetes arises from vascular dysfunction. A meta-analysis documented consistent evidence type 1 diabetes was strongly associated with endothelial and vascular smooth muscle dysfunction [47], appearing early, well before manifest cardiovascular disease. COVID-19 is marked by endothelial dysfunction [48], with the presence of a hypercoagulable state being strongly associated with adverse outcomes in COVID-19 [49]. However, there is now direct evidence implicating blood glucose as a causal mechanism in severe COVID-19. A study found that elevated glucose levels directly induce viral replication and proinflammatory cytokine expression in SARS-CoV-2-infected monocytes, which subsequently promotes T cell dysfunction and lung epithelial cell death [50]. MR data appear out of kilter with other evidence.

Previous MR studies have estimated the associations of genetic proxies of adiposity, cardiometabolic traits and metabolic biomarkers with COVID-19-related outcomes. In the UK Biobank and the HUNT study, genetically proxied higher BMI was associated with a higher risk of developing sepsis and severe COVID-19, while there was no strong evidence supporting an association of genetically proxied low-density lipoprotein cholesterol, systolic blood pressure or type 2 diabetes liability with risk of sepsis or severe COVID-19 [51]. A recent MR study using UK Biobank to evaluate the associations of 17 obesity-related cardiometabolic traits with COVID-19 susceptibility and severity, supported only BMI as a causal risk factor for COVID-19 hospitalisation independently or through its cardiometabolic consequences [52]. Future research is required to understand the mechanisms through which obesity is associated with a risk of poor health outcomes or mortality, and whether obesity-related conditions are along the causal pathway.

These results have implications for policy and practice. Excess total adiposity is probably causal for severe COVID-19. Ectopic fat accumulation is correlated with excess weight and appears to be crucial in causing metabolic disease [21], but the MR analyses suggest that metabolic disturbance of obesity may not itself cause severe COVID-19. In England, the Government issued a call to action to reduce risk of COVID-19 through weight loss. Ectopic fat is lost quickly during weight loss, rapidly normalising metabolic state [53, 54], while prolonged efforts are needed to reduce overall adiposity. However, the biggest risk to people with excess fat is non-communicable disease [55], which is particularly associated with ectopic fat and weight loss should reduce these complications [56]. The MR data contradict other evidence that suggests that glucose regulation may have a causal role on severe COVID-19. Thus, it remains uncertain whether tightening glycaemic control in diabetes reduces the risk from COVID-19, but there is clear evidence it prevents macro and microvascular disease for people with type 2 diabetes [57].

## Conclusion

Excess total adiposity measured by BMI and the proportion of body fat is strongly and probably casually associated with severe COVID-19. Mendelian randomisation data found no evidence that the strong observational associations of central fat distribution, insulin resistance and metabolic consequences of excess adiposity, or type 2 diabetes with COVID-19 were causal.

## Supplementary information

Supplementary material 1

Supplementary material 2

Supplementary material 3

Supplementary information

## Data Availability

Data to replicate the study can be applied through access at https://www.ukbiobank.ac.uk/enable-your-research/apply-for-access and at https://www.covid19hg.org/.
