# Supplementary material for: Associations between body composition, fat distribution and metabolic consequences of excess adiposity with severe COVID-19 outcomes: observational study and Mendelian randomisation analysis
Source: Int J Obes (Lond). 2022 Jan 14;46(5):943–50. doi: 10.1038/s41366-021-01054-3 (PMC8758930; doi:10.1038/s41366-021-01054-3)
Supplement: Supplementary file 1 — Supplementary material 1 [file 41366_2021_1054_MOESM1_ESM.docx]

**Supplementary material 1**

**Definitions of cases and controls for COVID-19 GWAS data**

In detail, the cases and controls used in the three COVID-19 GWAS summary data (our primary analyses) are defined as:

**1)** COVID-19 vs. population (Ncases = 38,984/ Ncontrols = 1,644,784)

**Cases:** individuals with laboratory confirmation of COVID-19 test positivity (RNA and/or serology based) OR EHR/ICD coding/ Physician Confirmed COVID-19 OR self-reported COVID-19 positive (e.g. by questionnaire)

**Controls:** everybody that is not a case, i.e. population

**2)** hospitalized COVID-19 vs. population (9,986/1,877,672)

**Cases:** Hospitalized laboratory confirmed COVID-19 test positivity (RNA and/or serology based), hospitalization due to corona-related symptoms.

**Controls:** everybody that is not a case, i.e. population

**3)** very severe respiratory confirmed COVID-19 vs. population (5,101/1,383,241)

**Cases:** Hospitalized laboratory confirmed COVID-19 test positivity (RNA and/or serology based), AND death OR respiratory support (intubation, CPAP, BiPAP, CNP (continue external negative pressure), Optiflow/very high flow Positive End Expiratory Pressure Oxygen AND hospitalization with COVID19 as primary reason for admission.

**Controls:** everybody that is not a case, i.e. population
