# Supplementary material for: Associations between body composition, fat distribution and metabolic consequences of excess adiposity with severe COVID-19 outcomes: observational study and Mendelian randomisation analysis
Source: Int J Obes (Lond). 2022 Jan 14;46(5):943–50. doi: 10.1038/s41366-021-01054-3 (PMC8758930; doi:10.1038/s41366-021-01054-3)
Supplement: Supplementary file 2 — Supplementary material 2 [file 41366_2021_1054_MOESM2_ESM.docx]

**Supplementary material 2**

| **Supplementary material 2. MR estimates from all exposures to the three COVID-19 outcomes across all the methods.** | | | | | | |
| --- | --- | --- | --- | --- | --- | --- |
| exposure | outcome | method | nsnp | b | se | pval |
| Adiponectin | covid_vs_pop | MR Egger | 17 | 0.0126294 | 0.06367066 | 0.84543057 |
| Adiponectin | covid_vs_pop | Weighted median | 17 | -0.0251299 | 0.03804394 | 0.50890062 |
| Adiponectin | covid_vs_pop | Inverse variance weighted | 17 | -0.0301527 | 0.04084365 | 0.46036421 |
| Adiponectin | covid_vs_pop | Simple mode | 17 | -0.0815643 | 0.07834818 | 0.31332887 |
| Adiponectin | covid_vs_pop | Weighted mode | 17 | -0.0301397 | 0.03513865 | 0.40370468 |
| Alanine aminotransferase \|\| id:ukb-d-30620_irnt | covid_vs_pop | MR Egger | 181 | -0.1788836 | 0.07217745 | 0.01412415 |
| Alanine aminotransferase \|\| id:ukb-d-30620_irnt | covid_vs_pop | Weighted median | 181 | -0.0567902 | 0.05415474 | 0.29433252 |
| Alanine aminotransferase \|\| id:ukb-d-30620_irnt | covid_vs_pop | Inverse variance weighted | 181 | 0.02963603 | 0.03706411 | 0.42394943 |
| Alanine aminotransferase \|\| id:ukb-d-30620_irnt | covid_vs_pop | Simple mode | 181 | 0.08852299 | 0.15346413 | 0.56477374 |
| Alanine aminotransferase \|\| id:ukb-d-30620_irnt | covid_vs_pop | Weighted mode | 181 | -0.1185544 | 0.09000009 | 0.18942251 |
| Albumin \|\| id:ukb-d-30600_irnt | covid_vs_pop | MR Egger | 188 | -0.0542255 | 0.06662998 | 0.41678273 |
| Albumin \|\| id:ukb-d-30600_irnt | covid_vs_pop | Weighted median | 188 | -0.0742701 | 0.05490439 | 0.17614597 |
| Albumin \|\| id:ukb-d-30600_irnt | covid_vs_pop | Inverse variance weighted | 188 | -0.0465994 | 0.03444362 | 0.17608222 |
| Albumin \|\| id:ukb-d-30600_irnt | covid_vs_pop | Simple mode | 188 | -0.1083507 | 0.13018451 | 0.40630952 |
| Albumin \|\| id:ukb-d-30600_irnt | covid_vs_pop | Weighted mode | 188 | -0.0905789 | 0.08540233 | 0.29023355 |
| Alkaline phosphatase \|\| id:ukb-d-30610_irnt | covid_vs_pop | MR Egger | 280 | 0.04901459 | 0.0400563 | 0.22212324 |
| Alkaline phosphatase \|\| id:ukb-d-30610_irnt | covid_vs_pop | Weighted median | 280 | 0.03857319 | 0.03862222 | 0.31792525 |
| Alkaline phosphatase \|\| id:ukb-d-30610_irnt | covid_vs_pop | Inverse variance weighted | 280 | 0.03289358 | 0.02402229 | 0.17090741 |
| Alkaline phosphatase \|\| id:ukb-d-30610_irnt | covid_vs_pop | Simple mode | 280 | 0.07321312 | 0.08829125 | 0.40768718 |
| Alkaline phosphatase \|\| id:ukb-d-30610_irnt | covid_vs_pop | Weighted mode | 280 | 0.03450478 | 0.05441184 | 0.52650839 |
| Apoliprotein A \|\| id:ukb-d-30630_irnt | covid_vs_pop | MR Egger | 233 | 0.08627228 | 0.04588115 | 0.06131973 |
| Apoliprotein A \|\| id:ukb-d-30630_irnt | covid_vs_pop | Weighted median | 233 | 0.02689274 | 0.04161957 | 0.51817821 |
| Apoliprotein A \|\| id:ukb-d-30630_irnt | covid_vs_pop | Inverse variance weighted | 233 | -0.008208 | 0.02799067 | 0.76933785 |
| Apoliprotein A \|\| id:ukb-d-30630_irnt | covid_vs_pop | Simple mode | 233 | -0.1142261 | 0.0932166 | 0.22167313 |
| Apoliprotein A \|\| id:ukb-d-30630_irnt | covid_vs_pop | Weighted mode | 233 | 0.05442431 | 0.04927531 | 0.27052283 |
| Apolipoprotein B \|\| id:ukb-d-30640_irnt | covid_vs_pop | MR Egger | 148 | 0.03908029 | 0.05411029 | 0.47130591 |
| Apolipoprotein B \|\| id:ukb-d-30640_irnt | covid_vs_pop | Weighted median | 148 | -0.0253673 | 0.04604618 | 0.58169544 |
| Apolipoprotein B \|\| id:ukb-d-30640_irnt | covid_vs_pop | Inverse variance weighted | 148 | -0.012791 | 0.03448364 | 0.71069055 |
| Apolipoprotein B \|\| id:ukb-d-30640_irnt | covid_vs_pop | Simple mode | 148 | -0.018605 | 0.0811232 | 0.81892133 |
| Apolipoprotein B \|\| id:ukb-d-30640_irnt | covid_vs_pop | Weighted mode | 148 | -0.018605 | 0.0411811 | 0.65208954 |
| Arm fat mass (left) \|\| id:ukb-b-8338 | covid_vs_pop | MR Egger | 406 | 0.1769408 | 0.08479075 | 0.03753318 |
| Arm fat mass (left) \|\| id:ukb-b-8338 | covid_vs_pop | Weighted median | 406 | 0.15130963 | 0.05337778 | 0.00458697 |
| Arm fat mass (left) \|\| id:ukb-b-8338 | covid_vs_pop | Inverse variance weighted | 406 | 0.16465227 | 0.03081155 | 9.10E-08 |
| Arm fat mass (left) \|\| id:ukb-b-8338 | covid_vs_pop | Simple mode | 406 | -0.0108867 | 0.15058021 | 0.94240038 |
| Arm fat mass (left) \|\| id:ukb-b-8338 | covid_vs_pop | Weighted mode | 406 | 0.0969695 | 0.09190566 | 0.29200943 |
| Arm fat mass (right) \|\| id:ukb-b-6704 | covid_vs_pop | MR Egger | 411 | 0.1592895 | 0.08497111 | 0.06155595 |
| Arm fat mass (right) \|\| id:ukb-b-6704 | covid_vs_pop | Weighted median | 411 | 0.15908381 | 0.05481762 | 0.00370722 |
| Arm fat mass (right) \|\| id:ukb-b-6704 | covid_vs_pop | Inverse variance weighted | 411 | 0.16284945 | 0.03124055 | 1.86E-07 |
| Arm fat mass (right) \|\| id:ukb-b-6704 | covid_vs_pop | Simple mode | 411 | -0.0073306 | 0.15022696 | 0.96110488 |
| Arm fat mass (right) \|\| id:ukb-b-6704 | covid_vs_pop | Weighted mode | 411 | 0.11073135 | 0.09733777 | 0.25595235 |
| Arm fat percentage (left) \|\| id:ukb-b-20188 | covid_vs_pop | MR Egger | 380 | 0.26070488 | 0.150517 | 0.08407816 |
| Arm fat percentage (left) \|\| id:ukb-b-20188 | covid_vs_pop | Weighted median | 380 | 0.25611983 | 0.07403336 | 0.00054114 |
| Arm fat percentage (left) \|\| id:ukb-b-20188 | covid_vs_pop | Inverse variance weighted | 380 | 0.23736715 | 0.05064342 | 2.77E-06 |
| Arm fat percentage (left) \|\| id:ukb-b-20188 | covid_vs_pop | Simple mode | 380 | 0.2936965 | 0.22144735 | 0.18555264 |
| Arm fat percentage (left) \|\| id:ukb-b-20188 | covid_vs_pop | Weighted mode | 380 | 0.21258348 | 0.12650514 | 0.09369696 |
| Arm fat percentage (right) \|\| id:ukb-b-12854 | covid_vs_pop | MR Egger | 378 | 0.26760334 | 0.14838304 | 0.07211588 |
| Arm fat percentage (right) \|\| id:ukb-b-12854 | covid_vs_pop | Weighted median | 378 | 0.26579725 | 0.07282021 | 0.00026219 |
| Arm fat percentage (right) \|\| id:ukb-b-12854 | covid_vs_pop | Inverse variance weighted | 378 | 0.2461865 | 0.05025954 | 9.67E-07 |
| Arm fat percentage (right) \|\| id:ukb-b-12854 | covid_vs_pop | Simple mode | 378 | 0.30460557 | 0.22038156 | 0.16773674 |
| Arm fat percentage (right) \|\| id:ukb-b-12854 | covid_vs_pop | Weighted mode | 378 | 0.26834905 | 0.14512423 | 0.06522619 |
| Arm fat-free mass (left) \|\| id:ukb-b-19925 | covid_vs_pop | MR Egger | 496 | 0.09811757 | 0.10516413 | 0.35127851 |
| Arm fat-free mass (left) \|\| id:ukb-b-19925 | covid_vs_pop | Weighted median | 496 | 0.14695749 | 0.06407287 | 0.02181354 |
| Arm fat-free mass (left) \|\| id:ukb-b-19925 | covid_vs_pop | Inverse variance weighted | 496 | 0.17677426 | 0.04171482 | 2.26E-05 |
| Arm fat-free mass (left) \|\| id:ukb-b-19925 | covid_vs_pop | Simple mode | 496 | -0.100118 | 0.19828638 | 0.61384231 |
| Arm fat-free mass (left) \|\| id:ukb-b-19925 | covid_vs_pop | Weighted mode | 496 | -0.0107209 | 0.14146093 | 0.939619 |
| Arm fat-free mass (right) \|\| id:ukb-b-19520 | covid_vs_pop | MR Egger | 497 | 0.13070872 | 0.1024685 | 0.2026956 |
| Arm fat-free mass (right) \|\| id:ukb-b-19520 | covid_vs_pop | Weighted median | 497 | 0.14856446 | 0.06578975 | 0.02393505 |
| Arm fat-free mass (right) \|\| id:ukb-b-19520 | covid_vs_pop | Inverse variance weighted | 497 | 0.16395944 | 0.04043409 | 5.01E-05 |
| Arm fat-free mass (right) \|\| id:ukb-b-19520 | covid_vs_pop | Simple mode | 497 | 0.45074022 | 0.20236926 | 0.02637416 |
| Arm fat-free mass (right) \|\| id:ukb-b-19520 | covid_vs_pop | Weighted mode | 497 | 0.04592217 | 0.14743023 | 0.75556349 |
| Aspartate aminotransferase \|\| id:ukb-d-30650_irnt | covid_vs_pop | MR Egger | 218 | -0.0433718 | 0.07886645 | 0.58292907 |
| Aspartate aminotransferase \|\| id:ukb-d-30650_irnt | covid_vs_pop | Weighted median | 218 | 0.00032075 | 0.05400415 | 0.99526114 |
| Aspartate aminotransferase \|\| id:ukb-d-30650_irnt | covid_vs_pop | Inverse variance weighted | 218 | -0.0035306 | 0.03780212 | 0.92558748 |
| Aspartate aminotransferase \|\| id:ukb-d-30650_irnt | covid_vs_pop | Simple mode | 218 | 0.0076966 | 0.13968417 | 0.95610947 |
| Aspartate aminotransferase \|\| id:ukb-d-30650_irnt | covid_vs_pop | Weighted mode | 218 | 0.03175452 | 0.10369331 | 0.75971932 |
| Body fat percentage \|\| id:ukb-b-8909 | covid_vs_pop | MR Egger | 376 | 0.35250949 | 0.14330977 | 0.01435417 |
| Body fat percentage \|\| id:ukb-b-8909 | covid_vs_pop | Weighted median | 376 | 0.29819586 | 0.07185072 | 3.32E-05 |
| Body fat percentage \|\| id:ukb-b-8909 | covid_vs_pop | Inverse variance weighted | 376 | 0.23616078 | 0.04472052 | 1.29E-07 |
| Body fat percentage \|\| id:ukb-b-8909 | covid_vs_pop | Simple mode | 376 | 0.4112549 | 0.24349183 | 0.09205288 |
| Body fat percentage \|\| id:ukb-b-8909 | covid_vs_pop | Weighted mode | 376 | 0.34991384 | 0.15418759 | 0.02381111 |
| C-reactive protein \|\| id:ukb-d-30710_irnt | covid_vs_pop | MR Egger | 187 | 0.02557456 | 0.05546854 | 0.64529413 |
| C-reactive protein \|\| id:ukb-d-30710_irnt | covid_vs_pop | Weighted median | 187 | 0.02486483 | 0.04611977 | 0.58979296 |
| C-reactive protein \|\| id:ukb-d-30710_irnt | covid_vs_pop | Inverse variance weighted | 187 | 0.07361865 | 0.03280472 | 0.02482288 |
| C-reactive protein \|\| id:ukb-d-30710_irnt | covid_vs_pop | Simple mode | 187 | 0.11410526 | 0.09142171 | 0.21355602 |
| C-reactive protein \|\| id:ukb-d-30710_irnt | covid_vs_pop | Weighted mode | 187 | 0.04222349 | 0.04287881 | 0.32604237 |
| Calcium \|\| id:ukb-d-30680_irnt | covid_vs_pop | MR Egger | 191 | -0.0234965 | 0.07269711 | 0.74689276 |
| Calcium \|\| id:ukb-d-30680_irnt | covid_vs_pop | Weighted median | 191 | 0.01346473 | 0.05045624 | 0.78957734 |
| Calcium \|\| id:ukb-d-30680_irnt | covid_vs_pop | Inverse variance weighted | 191 | -0.0015806 | 0.03219898 | 0.96084796 |
| Calcium \|\| id:ukb-d-30680_irnt | covid_vs_pop | Simple mode | 191 | -0.0825026 | 0.11932215 | 0.49014104 |
| Calcium \|\| id:ukb-d-30680_irnt | covid_vs_pop | Weighted mode | 191 | -0.0159484 | 0.09046046 | 0.86024403 |
| Cholesterol \|\| id:ukb-d-30690_irnt | covid_vs_pop | MR Egger | 163 | 0.12593787 | 0.05545816 | 0.02448063 |
| Cholesterol \|\| id:ukb-d-30690_irnt | covid_vs_pop | Weighted median | 163 | 0.07611485 | 0.04268275 | 0.07454242 |
| Cholesterol \|\| id:ukb-d-30690_irnt | covid_vs_pop | Inverse variance weighted | 163 | 0.05097807 | 0.03381306 | 0.1316456 |
| Cholesterol \|\| id:ukb-d-30690_irnt | covid_vs_pop | Simple mode | 163 | 0.06556133 | 0.07913362 | 0.40861258 |
| Cholesterol \|\| id:ukb-d-30690_irnt | covid_vs_pop | Weighted mode | 163 | 0.07601546 | 0.04947836 | 0.12640643 |
| Creatinine \|\| id:ukb-d-30700_irnt | covid_vs_pop | MR Egger | 306 | -0.0119591 | 0.0760004 | 0.87506913 |
| Creatinine \|\| id:ukb-d-30700_irnt | covid_vs_pop | Weighted median | 306 | -0.0154428 | 0.04930701 | 0.75413122 |
| Creatinine \|\| id:ukb-d-30700_irnt | covid_vs_pop | Inverse variance weighted | 306 | -0.0046369 | 0.03269897 | 0.88723347 |
| Creatinine \|\| id:ukb-d-30700_irnt | covid_vs_pop | Simple mode | 306 | -0.096823 | 0.11641604 | 0.40622996 |
| Creatinine \|\| id:ukb-d-30700_irnt | covid_vs_pop | Weighted mode | 306 | -0.0406588 | 0.0791728 | 0.6079416 |
| Cystatin C \|\| id:ukb-d-30720_irnt | covid_vs_pop | MR Egger | 288 | -0.0946072 | 0.05897686 | 0.10978663 |
| Cystatin C \|\| id:ukb-d-30720_irnt | covid_vs_pop | Weighted median | 288 | -0.027967 | 0.04689104 | 0.55089067 |
| Cystatin C \|\| id:ukb-d-30720_irnt | covid_vs_pop | Inverse variance weighted | 288 | -0.0238969 | 0.02979762 | 0.42256814 |
| Cystatin C \|\| id:ukb-d-30720_irnt | covid_vs_pop | Simple mode | 288 | 0.00178991 | 0.1092218 | 0.98693633 |
| Cystatin C \|\| id:ukb-d-30720_irnt | covid_vs_pop | Weighted mode | 288 | -0.0308331 | 0.06288251 | 0.62427598 |
| Diastolic blood pressure, automated reading \|\| id:ukb-b-7992 | covid_vs_pop | MR Egger | 246 | -0.0769993 | 0.14341529 | 0.59182769 |
| Diastolic blood pressure, automated reading \|\| id:ukb-b-7992 | covid_vs_pop | Weighted median | 246 | -0.0477186 | 0.06175844 | 0.43972083 |
| Diastolic blood pressure, automated reading \|\| id:ukb-b-7992 | covid_vs_pop | Inverse variance weighted | 246 | -0.0813931 | 0.04678805 | 0.08192683 |
| Diastolic blood pressure, automated reading \|\| id:ukb-b-7992 | covid_vs_pop | Simple mode | 246 | 0.18949569 | 0.1902038 | 0.32009847 |
| Diastolic blood pressure, automated reading \|\| id:ukb-b-7992 | covid_vs_pop | Weighted mode | 246 | 0.05601669 | 0.15792615 | 0.72311812 |
| Direct bilirubin \|\| id:ukb-d-30660_irnt | covid_vs_pop | MR Egger | 76 | -0.0283972 | 0.05549648 | 0.61038865 |
| Direct bilirubin \|\| id:ukb-d-30660_irnt | covid_vs_pop | Weighted median | 76 | -0.0632618 | 0.049405 | 0.20037899 |
| Direct bilirubin \|\| id:ukb-d-30660_irnt | covid_vs_pop | Inverse variance weighted | 76 | -0.0250109 | 0.04172765 | 0.54891737 |
| Direct bilirubin \|\| id:ukb-d-30660_irnt | covid_vs_pop | Simple mode | 76 | -0.0372368 | 0.0756794 | 0.62413281 |
| Direct bilirubin \|\| id:ukb-d-30660_irnt | covid_vs_pop | Weighted mode | 76 | -0.0372368 | 0.04518552 | 0.41250325 |
| Gamma glutamyltransferase \|\| id:ukb-d-30730_irnt | covid_vs_pop | MR Egger | 255 | -0.0355324 | 0.04739578 | 0.45413502 |
| Gamma glutamyltransferase \|\| id:ukb-d-30730_irnt | covid_vs_pop | Weighted median | 255 | -0.041585 | 0.04066031 | 0.30642937 |
| Gamma glutamyltransferase \|\| id:ukb-d-30730_irnt | covid_vs_pop | Inverse variance weighted | 255 | -0.0063464 | 0.02657658 | 0.81126219 |
| Gamma glutamyltransferase \|\| id:ukb-d-30730_irnt | covid_vs_pop | Simple mode | 255 | 0.1343741 | 0.0920782 | 0.14570505 |
| Gamma glutamyltransferase \|\| id:ukb-d-30730_irnt | covid_vs_pop | Weighted mode | 255 | -0.0644531 | 0.04295108 | 0.13469695 |
| Glucose \|\| id:ukb-d-30740_irnt | covid_vs_pop | MR Egger | 103 | -0.1276288 | 0.09092778 | 0.16349671 |
| Glucose \|\| id:ukb-d-30740_irnt | covid_vs_pop | Weighted median | 103 | -0.0324254 | 0.05948541 | 0.58568535 |
| Glucose \|\| id:ukb-d-30740_irnt | covid_vs_pop | Inverse variance weighted | 103 | -0.0329671 | 0.04895546 | 0.50068704 |
| Glucose \|\| id:ukb-d-30740_irnt | covid_vs_pop | Simple mode | 103 | -0.0066275 | 0.11116813 | 0.95257736 |
| Glucose \|\| id:ukb-d-30740_irnt | covid_vs_pop | Weighted mode | 103 | -0.0397958 | 0.05694354 | 0.48622708 |
| Glycated haemoglobin \|\| id:ukb-d-30750_irnt | covid_vs_pop | MR Egger | 277 | -0.0034951 | 0.04997786 | 0.94429743 |
| Glycated haemoglobin \|\| id:ukb-d-30750_irnt | covid_vs_pop | Weighted median | 277 | -0.0157565 | 0.04020656 | 0.69514 |
| Glycated haemoglobin \|\| id:ukb-d-30750_irnt | covid_vs_pop | Inverse variance weighted | 277 | 0.03108384 | 0.02555203 | 0.22379757 |
| Glycated haemoglobin \|\| id:ukb-d-30750_irnt | covid_vs_pop | Simple mode | 277 | 0.03143469 | 0.10132964 | 0.75662625 |
| Glycated haemoglobin \|\| id:ukb-d-30750_irnt | covid_vs_pop | Weighted mode | 277 | -0.0458191 | 0.06122989 | 0.45490863 |
| HDL cholesterol \|\| id:ukb-d-30760_irnt | covid_vs_pop | MR Egger | 252 | 0.01996886 | 0.04592309 | 0.66405899 |
| HDL cholesterol \|\| id:ukb-d-30760_irnt | covid_vs_pop | Weighted median | 252 | 0.02000883 | 0.03851198 | 0.60337844 |
| HDL cholesterol \|\| id:ukb-d-30760_irnt | covid_vs_pop | Inverse variance weighted | 252 | -0.0341976 | 0.02749082 | 0.21351236 |
| HDL cholesterol \|\| id:ukb-d-30760_irnt | covid_vs_pop | Simple mode | 252 | -0.1635938 | 0.08732856 | 0.0621872 |
| HDL cholesterol \|\| id:ukb-d-30760_irnt | covid_vs_pop | Weighted mode | 252 | 0.02444459 | 0.04330455 | 0.57293108 |
| Hip circumference \|\| id:ukb-b-15590 | covid_vs_pop | MR Egger | 401 | 0.06814331 | 0.08504203 | 0.4234408 |
| Hip circumference \|\| id:ukb-b-15590 | covid_vs_pop | Weighted median | 401 | 0.12520056 | 0.0506589 | 0.01345692 |
| Hip circumference \|\| id:ukb-b-15590 | covid_vs_pop | Inverse variance weighted | 401 | 0.14369659 | 0.03118842 | 4.08E-06 |
| Hip circumference \|\| id:ukb-b-15590 | covid_vs_pop | Simple mode | 401 | 0.02046616 | 0.14984088 | 0.89142681 |
| Hip circumference \|\| id:ukb-b-15590 | covid_vs_pop | Weighted mode | 401 | 0.08571834 | 0.09572831 | 0.37109392 |
| IGF-1 \|\| id:ukb-d-30770_irnt | covid_vs_pop | MR Egger | 323 | -0.0608859 | 0.05037552 | 0.22769027 |
| IGF-1 \|\| id:ukb-d-30770_irnt | covid_vs_pop | Weighted median | 323 | 0.01907605 | 0.03854841 | 0.62069919 |
| IGF-1 \|\| id:ukb-d-30770_irnt | covid_vs_pop | Inverse variance weighted | 323 | 0.00872434 | 0.02482456 | 0.72525844 |
| IGF-1 \|\| id:ukb-d-30770_irnt | covid_vs_pop | Simple mode | 323 | -0.0023836 | 0.09556453 | 0.98011628 |
| IGF-1 \|\| id:ukb-d-30770_irnt | covid_vs_pop | Weighted mode | 323 | 0.00839103 | 0.05220282 | 0.8723998 |
| Insulin Resistance | covid_vs_pop | MR Egger | 49 | -0.0571991 | 0.14190246 | 0.68871168 |
| Insulin Resistance | covid_vs_pop | Weighted median | 49 | -0.0411648 | 0.11531037 | 0.72109805 |
| Insulin Resistance | covid_vs_pop | Inverse variance weighted | 49 | 0.04765136 | 0.07239356 | 0.51039263 |
| Insulin Resistance | covid_vs_pop | Simple mode | 49 | 0.14988201 | 0.22846142 | 0.5149249 |
| Insulin Resistance | covid_vs_pop | Weighted mode | 49 | -0.065893 | 0.11013292 | 0.55244957 |
| LDL direct \|\| id:ukb-d-30780_irnt | covid_vs_pop | MR Egger | 143 | 0.07184043 | 0.05951362 | 0.22940486 |
| LDL direct \|\| id:ukb-d-30780_irnt | covid_vs_pop | Weighted median | 143 | 0.07152048 | 0.04759435 | 0.13291396 |
| LDL direct \|\| id:ukb-d-30780_irnt | covid_vs_pop | Inverse variance weighted | 143 | 0.04144241 | 0.03758053 | 0.2701302 |
| LDL direct \|\| id:ukb-d-30780_irnt | covid_vs_pop | Simple mode | 143 | 0.11670625 | 0.09273569 | 0.21028249 |
| LDL direct \|\| id:ukb-d-30780_irnt | covid_vs_pop | Weighted mode | 143 | 0.06320532 | 0.04943501 | 0.20313995 |
| Leg fat mass (left) \|\| id:ukb-b-7212 | covid_vs_pop | MR Egger | 403 | 0.21620385 | 0.11212869 | 0.05453899 |
| Leg fat mass (left) \|\| id:ukb-b-7212 | covid_vs_pop | Weighted median | 403 | 0.25107314 | 0.06791698 | 0.00021836 |
| Leg fat mass (left) \|\| id:ukb-b-7212 | covid_vs_pop | Inverse variance weighted | 403 | 0.2203522 | 0.03992122 | 3.40E-08 |
| Leg fat mass (left) \|\| id:ukb-b-7212 | covid_vs_pop | Simple mode | 403 | 0.00495088 | 0.18457129 | 0.97861366 |
| Leg fat mass (left) \|\| id:ukb-b-7212 | covid_vs_pop | Weighted mode | 403 | 0.17891669 | 0.12385034 | 0.1493436 |
| Leg fat mass (right) \|\| id:ukb-b-18096 | covid_vs_pop | MR Egger | 407 | 0.15176614 | 0.11346357 | 0.18178534 |
| Leg fat mass (right) \|\| id:ukb-b-18096 | covid_vs_pop | Weighted median | 407 | 0.24548252 | 0.06903069 | 0.00037635 |
| Leg fat mass (right) \|\| id:ukb-b-18096 | covid_vs_pop | Inverse variance weighted | 407 | 0.2363167 | 0.04017164 | 4.04E-09 |
| Leg fat mass (right) \|\| id:ukb-b-18096 | covid_vs_pop | Simple mode | 407 | -0.0372462 | 0.20767554 | 0.85775378 |
| Leg fat mass (right) \|\| id:ukb-b-18096 | covid_vs_pop | Weighted mode | 407 | 0.14913582 | 0.12365883 | 0.22850962 |
| Leg fat percentage (left) \|\| id:ukb-b-18377 | covid_vs_pop | MR Egger | 361 | 0.39721502 | 0.18832449 | 0.03561883 |
| Leg fat percentage (left) \|\| id:ukb-b-18377 | covid_vs_pop | Weighted median | 361 | 0.3729934 | 0.09231481 | 5.33E-05 |
| Leg fat percentage (left) \|\| id:ukb-b-18377 | covid_vs_pop | Inverse variance weighted | 361 | 0.31274513 | 0.05746132 | 5.25E-08 |
| Leg fat percentage (left) \|\| id:ukb-b-18377 | covid_vs_pop | Simple mode | 361 | 0.90153718 | 0.29869917 | 0.00272385 |
| Leg fat percentage (left) \|\| id:ukb-b-18377 | covid_vs_pop | Weighted mode | 361 | 0.66129819 | 0.21976766 | 0.00280497 |
| Leg fat percentage (right) \|\| id:ukb-b-20531 | covid_vs_pop | MR Egger | 367 | 0.35322377 | 0.18013975 | 0.05065872 |
| Leg fat percentage (right) \|\| id:ukb-b-20531 | covid_vs_pop | Weighted median | 367 | 0.36104035 | 0.08542736 | 2.38E-05 |
| Leg fat percentage (right) \|\| id:ukb-b-20531 | covid_vs_pop | Inverse variance weighted | 367 | 0.30011865 | 0.05554609 | 6.55E-08 |
| Leg fat percentage (right) \|\| id:ukb-b-20531 | covid_vs_pop | Simple mode | 367 | 0.61472017 | 0.30719773 | 0.04612424 |
| Leg fat percentage (right) \|\| id:ukb-b-20531 | covid_vs_pop | Weighted mode | 367 | 0.50818111 | 0.21688107 | 0.01965738 |
| Leg fat-free mass (left) \|\| id:ukb-b-16099 | covid_vs_pop | MR Egger | 487 | 0.16183392 | 0.09358715 | 0.08440383 |
| Leg fat-free mass (left) \|\| id:ukb-b-16099 | covid_vs_pop | Weighted median | 487 | 0.09743301 | 0.06189356 | 0.1154406 |
| Leg fat-free mass (left) \|\| id:ukb-b-16099 | covid_vs_pop | Inverse variance weighted | 487 | 0.15936563 | 0.03833072 | 3.22E-05 |
| Leg fat-free mass (left) \|\| id:ukb-b-16099 | covid_vs_pop | Simple mode | 487 | 0.03106738 | 0.16849829 | 0.85379382 |
| Leg fat-free mass (left) \|\| id:ukb-b-16099 | covid_vs_pop | Weighted mode | 487 | 0.03106738 | 0.12314401 | 0.80092748 |
| Leg fat-free mass (right) \|\| id:ukb-b-12828 | covid_vs_pop | MR Egger | 492 | 0.09488665 | 0.09308448 | 0.30853481 |
| Leg fat-free mass (right) \|\| id:ukb-b-12828 | covid_vs_pop | Weighted median | 492 | 0.09244458 | 0.0617522 | 0.1343868 |
| Leg fat-free mass (right) \|\| id:ukb-b-12828 | covid_vs_pop | Inverse variance weighted | 492 | 0.12730463 | 0.03804728 | 0.00081999 |
| Leg fat-free mass (right) \|\| id:ukb-b-12828 | covid_vs_pop | Simple mode | 492 | -0.0375518 | 0.18183297 | 0.83647175 |
| Leg fat-free mass (right) \|\| id:ukb-b-12828 | covid_vs_pop | Weighted mode | 492 | 0.01970519 | 0.13183033 | 0.88124112 |
| Lipoprotein A \|\| id:ukb-d-30790_irnt | covid_vs_pop | MR Egger | 14 | -0.091491 | 0.09193705 | 0.33930814 |
| Lipoprotein A \|\| id:ukb-d-30790_irnt | covid_vs_pop | Weighted median | 14 | -0.1274271 | 0.06322147 | 0.04384524 |
| Lipoprotein A \|\| id:ukb-d-30790_irnt | covid_vs_pop | Inverse variance weighted | 14 | -0.1103272 | 0.05339175 | 0.03879349 |
| Lipoprotein A \|\| id:ukb-d-30790_irnt | covid_vs_pop | Simple mode | 14 | -0.0284391 | 0.10262208 | 0.78604216 |
| Lipoprotein A \|\| id:ukb-d-30790_irnt | covid_vs_pop | Weighted mode | 14 | -0.118419 | 0.07886776 | 0.15712364 |
| Oestradiol \|\| id:ukb-d-30800_irnt | covid_vs_pop | Wald ratio | 1 | -0.1804381 | 0.31845689 | 0.570985 |
| Phosphate \|\| id:ukb-d-30810_irnt | covid_vs_pop | MR Egger | 137 | -0.0349411 | 0.0657645 | 0.59607979 |
| Phosphate \|\| id:ukb-d-30810_irnt | covid_vs_pop | Weighted median | 137 | -0.0373865 | 0.05577203 | 0.50263833 |
| Phosphate \|\| id:ukb-d-30810_irnt | covid_vs_pop | Inverse variance weighted | 137 | 0.00629099 | 0.03507347 | 0.85765039 |
| Phosphate \|\| id:ukb-d-30810_irnt | covid_vs_pop | Simple mode | 137 | -0.1031904 | 0.10664976 | 0.33498016 |
| Phosphate \|\| id:ukb-d-30810_irnt | covid_vs_pop | Weighted mode | 137 | -0.025461 | 0.06474359 | 0.69474428 |
| SHBG \|\| id:ukb-d-30830_irnt | covid_vs_pop | MR Egger | 237 | 0.03692269 | 0.04815896 | 0.44403906 |
| SHBG \|\| id:ukb-d-30830_irnt | covid_vs_pop | Weighted median | 237 | -0.0014656 | 0.0422914 | 0.97235418 |
| SHBG \|\| id:ukb-d-30830_irnt | covid_vs_pop | Inverse variance weighted | 237 | -0.0244133 | 0.02699358 | 0.36577655 |
| SHBG \|\| id:ukb-d-30830_irnt | covid_vs_pop | Simple mode | 237 | -0.1634239 | 0.10420448 | 0.11815098 |
| SHBG \|\| id:ukb-d-30830_irnt | covid_vs_pop | Weighted mode | 237 | -0.0125225 | 0.05480037 | 0.81944632 |
| Systolic blood pressure, automated reading \|\| id:ukb-b-20175 | covid_vs_pop | MR Egger | 231 | 0.15681336 | 0.13581363 | 0.24944885 |
| Systolic blood pressure, automated reading \|\| id:ukb-b-20175 | covid_vs_pop | Weighted median | 231 | -0.0433202 | 0.06781494 | 0.52295301 |
| Systolic blood pressure, automated reading \|\| id:ukb-b-20175 | covid_vs_pop | Inverse variance weighted | 231 | -0.0335466 | 0.04455126 | 0.45145604 |
| Systolic blood pressure, automated reading \|\| id:ukb-b-20175 | covid_vs_pop | Simple mode | 231 | -0.219941 | 0.20323589 | 0.28029899 |
| Systolic blood pressure, automated reading \|\| id:ukb-b-20175 | covid_vs_pop | Weighted mode | 231 | -0.1184272 | 0.16348178 | 0.46955279 |
| Testosterone \|\| id:ukb-d-30850_irnt | covid_vs_pop | MR Egger | 89 | -0.2455085 | 0.13257386 | 0.06743751 |
| Testosterone \|\| id:ukb-d-30850_irnt | covid_vs_pop | Weighted median | 89 | -0.0996694 | 0.11553841 | 0.38832901 |
| Testosterone \|\| id:ukb-d-30850_irnt | covid_vs_pop | Inverse variance weighted | 89 | 0.0269645 | 0.07548969 | 0.72094623 |
| Testosterone \|\| id:ukb-d-30850_irnt | covid_vs_pop | Simple mode | 89 | -0.2810034 | 0.24974487 | 0.26358 |
| Testosterone \|\| id:ukb-d-30850_irnt | covid_vs_pop | Weighted mode | 89 | -0.1037539 | 0.12782757 | 0.41917073 |
| Total bilirubin \|\| id:ukb-d-30840_irnt | covid_vs_pop | MR Egger | 121 | 0.08353301 | 0.05612473 | 0.139305 |
| Total bilirubin \|\| id:ukb-d-30840_irnt | covid_vs_pop | Weighted median | 121 | 0.03081677 | 0.05064088 | 0.54283241 |
| Total bilirubin \|\| id:ukb-d-30840_irnt | covid_vs_pop | Inverse variance weighted | 121 | 0.00326459 | 0.03984678 | 0.93470345 |
| Total bilirubin \|\| id:ukb-d-30840_irnt | covid_vs_pop | Simple mode | 121 | 0.06241997 | 0.08773439 | 0.47817674 |
| Total bilirubin \|\| id:ukb-d-30840_irnt | covid_vs_pop | Weighted mode | 121 | 0.04783978 | 0.04409198 | 0.28009677 |
| Total protein \|\| id:ukb-d-30860_irnt | covid_vs_pop | MR Egger | 214 | -0.0442958 | 0.07471995 | 0.55393094 |
| Total protein \|\| id:ukb-d-30860_irnt | covid_vs_pop | Weighted median | 214 | -0.0178803 | 0.05388007 | 0.74000013 |
| Total protein \|\| id:ukb-d-30860_irnt | covid_vs_pop | Inverse variance weighted | 214 | -0.0276519 | 0.03381911 | 0.41356126 |
| Total protein \|\| id:ukb-d-30860_irnt | covid_vs_pop | Simple mode | 214 | -0.1004884 | 0.15629285 | 0.52094824 |
| Total protein \|\| id:ukb-d-30860_irnt | covid_vs_pop | Weighted mode | 214 | -0.028201 | 0.109983 | 0.79788002 |
| Triglycerides \|\| id:ukb-d-30870_irnt | covid_vs_pop | MR Egger | 213 | 0.10217744 | 0.04397586 | 0.02110496 |
| Triglycerides \|\| id:ukb-d-30870_irnt | covid_vs_pop | Weighted median | 213 | 0.07176326 | 0.03909419 | 0.06640942 |
| Triglycerides \|\| id:ukb-d-30870_irnt | covid_vs_pop | Inverse variance weighted | 213 | 0.09821902 | 0.02717373 | 0.00030095 |
| Triglycerides \|\| id:ukb-d-30870_irnt | covid_vs_pop | Simple mode | 213 | 0.02515944 | 0.08170583 | 0.75844012 |
| Triglycerides \|\| id:ukb-d-30870_irnt | covid_vs_pop | Weighted mode | 213 | 0.07393908 | 0.03820832 | 0.05430141 |
| Trunk fat mass \|\| id:ukb-b-20044 | covid_vs_pop | MR Egger | 405 | 0.25561563 | 0.08431427 | 0.00258884 |
| Trunk fat mass \|\| id:ukb-b-20044 | covid_vs_pop | Weighted median | 405 | 0.20336837 | 0.05017896 | 5.06E-05 |
| Trunk fat mass \|\| id:ukb-b-20044 | covid_vs_pop | Inverse variance weighted | 405 | 0.17650008 | 0.03010336 | 4.54E-09 |
| Trunk fat mass \|\| id:ukb-b-20044 | covid_vs_pop | Simple mode | 405 | 0.05765576 | 0.14529577 | 0.69171232 |
| Trunk fat mass \|\| id:ukb-b-20044 | covid_vs_pop | Weighted mode | 405 | 0.15443987 | 0.09785646 | 0.11529572 |
| Trunk fat percentage \|\| id:ukb-b-16407 | covid_vs_pop | MR Egger | 371 | 0.34444613 | 0.11558324 | 0.00307273 |
| Trunk fat percentage \|\| id:ukb-b-16407 | covid_vs_pop | Weighted median | 371 | 0.23944884 | 0.05528674 | 1.48E-05 |
| Trunk fat percentage \|\| id:ukb-b-16407 | covid_vs_pop | Inverse variance weighted | 371 | 0.18021718 | 0.0363924 | 7.34E-07 |
| Trunk fat percentage \|\| id:ukb-b-16407 | covid_vs_pop | Simple mode | 371 | 0.35072102 | 0.1889151 | 0.0641769 |
| Trunk fat percentage \|\| id:ukb-b-16407 | covid_vs_pop | Weighted mode | 371 | 0.30293518 | 0.13004321 | 0.02037031 |
| Trunk fat-free mass \|\| id:ukb-b-17409 | covid_vs_pop | MR Egger | 539 | 0.10018641 | 0.08688587 | 0.24939029 |
| Trunk fat-free mass \|\| id:ukb-b-17409 | covid_vs_pop | Weighted median | 539 | 0.12157024 | 0.05672992 | 0.03211589 |
| Trunk fat-free mass \|\| id:ukb-b-17409 | covid_vs_pop | Inverse variance weighted | 539 | 0.1327105 | 0.03650205 | 0.00027723 |
| Trunk fat-free mass \|\| id:ukb-b-17409 | covid_vs_pop | Simple mode | 539 | 0.20088448 | 0.17575594 | 0.25355773 |
| Trunk fat-free mass \|\| id:ukb-b-17409 | covid_vs_pop | Weighted mode | 539 | 0.06437884 | 0.13094399 | 0.6231661 |
| Type 2 diabetes \|\| id:ebi-a-GCST006867 | covid_vs_pop | MR Egger | 115 | -0.051224 | 0.03512367 | 0.14750703 |
| Type 2 diabetes \|\| id:ebi-a-GCST006867 | covid_vs_pop | Weighted median | 115 | -0.0579491 | 0.02384435 | 0.01508595 |
| Type 2 diabetes \|\| id:ebi-a-GCST006867 | covid_vs_pop | Inverse variance weighted | 115 | -0.0118666 | 0.014956 | 0.42752454 |
| Type 2 diabetes \|\| id:ebi-a-GCST006867 | covid_vs_pop | Simple mode | 115 | 0.00079029 | 0.060233 | 0.9895545 |
| Type 2 diabetes \|\| id:ebi-a-GCST006867 | covid_vs_pop | Weighted mode | 115 | -0.0639976 | 0.03114061 | 0.04215292 |
| Urate \|\| id:ukb-d-30880_irnt | covid_vs_pop | MR Egger | 228 | -0.0724972 | 0.05343154 | 0.1761905 |
| Urate \|\| id:ukb-d-30880_irnt | covid_vs_pop | Weighted median | 228 | -0.050634 | 0.05353196 | 0.34421749 |
| Urate \|\| id:ukb-d-30880_irnt | covid_vs_pop | Inverse variance weighted | 228 | -0.0107919 | 0.03294357 | 0.74322435 |
| Urate \|\| id:ukb-d-30880_irnt | covid_vs_pop | Simple mode | 228 | -0.0702484 | 0.13369837 | 0.59980108 |
| Urate \|\| id:ukb-d-30880_irnt | covid_vs_pop | Weighted mode | 228 | -0.0477088 | 0.06596295 | 0.47026082 |
| Urea \|\| id:ukb-d-30670_irnt | covid_vs_pop | MR Egger | 152 | -0.0472459 | 0.10872066 | 0.66450498 |
| Urea \|\| id:ukb-d-30670_irnt | covid_vs_pop | Weighted median | 152 | 0.02007526 | 0.06154364 | 0.74427637 |
| Urea \|\| id:ukb-d-30670_irnt | covid_vs_pop | Inverse variance weighted | 152 | -0.0048883 | 0.04714003 | 0.91740963 |
| Urea \|\| id:ukb-d-30670_irnt | covid_vs_pop | Simple mode | 152 | 0.06726728 | 0.1408302 | 0.63359136 |
| Urea \|\| id:ukb-d-30670_irnt | covid_vs_pop | Weighted mode | 152 | 0.03527803 | 0.09961447 | 0.72372377 |
| Vitamin D \|\| id:ukb-d-30890_irnt | covid_vs_pop | MR Egger | 56 | -0.1133741 | 0.06299199 | 0.07747699 |
| Vitamin D \|\| id:ukb-d-30890_irnt | covid_vs_pop | Weighted median | 56 | -0.0655541 | 0.05494401 | 0.23282718 |
| Vitamin D \|\| id:ukb-d-30890_irnt | covid_vs_pop | Inverse variance weighted | 56 | -0.0790489 | 0.04110153 | 0.05444764 |
| Vitamin D \|\| id:ukb-d-30890_irnt | covid_vs_pop | Simple mode | 56 | -0.0581115 | 0.10644501 | 0.58732192 |
| Vitamin D \|\| id:ukb-d-30890_irnt | covid_vs_pop | Weighted mode | 56 | -0.0657086 | 0.04696548 | 0.1674024 |
| Waist circumference \|\| id:ukb-b-9405 | covid_vs_pop | MR Egger | 356 | 0.18787926 | 0.11017706 | 0.08902535 |
| Waist circumference \|\| id:ukb-b-9405 | covid_vs_pop | Weighted median | 356 | 0.17728191 | 0.06695162 | 0.00809909 |
| Waist circumference \|\| id:ukb-b-9405 | covid_vs_pop | Inverse variance weighted | 356 | 0.20860578 | 0.03839633 | 5.54E-08 |
| Waist circumference \|\| id:ukb-b-9405 | covid_vs_pop | Simple mode | 356 | 0.03210808 | 0.18027259 | 0.85873936 |
| Waist circumference \|\| id:ukb-b-9405 | covid_vs_pop | Weighted mode | 356 | 0.08295692 | 0.11753272 | 0.4807631 |
| Waist-to-hip ratio \|\| id:ieu-a-73 | covid_vs_pop | MR Egger | 29 | 0.35849047 | 0.33659392 | 0.29628408 |
| Waist-to-hip ratio \|\| id:ieu-a-73 | covid_vs_pop | Weighted median | 29 | 0.26735936 | 0.10894925 | 0.01412845 |
| Waist-to-hip ratio \|\| id:ieu-a-73 | covid_vs_pop | Inverse variance weighted | 29 | 0.14322878 | 0.07757564 | 0.06484702 |
| Waist-to-hip ratio \|\| id:ieu-a-73 | covid_vs_pop | Simple mode | 29 | 0.37340587 | 0.23311262 | 0.12041737 |
| Waist-to-hip ratio \|\| id:ieu-a-73 | covid_vs_pop | Weighted mode | 29 | 0.32153083 | 0.17749297 | 0.08080182 |
| Weight \|\| id:ukb-b-11842 | covid_vs_pop | MR Egger | 480 | 0.11324035 | 0.07787644 | 0.14657406 |
| Weight \|\| id:ukb-b-11842 | covid_vs_pop | Weighted median | 480 | 0.10169421 | 0.05150494 | 0.04832997 |
| Weight \|\| id:ukb-b-11842 | covid_vs_pop | Inverse variance weighted | 480 | 0.16589095 | 0.03049203 | 5.31E-08 |
| Weight \|\| id:ukb-b-11842 | covid_vs_pop | Simple mode | 480 | -0.0228855 | 0.15180485 | 0.88023151 |
| Weight \|\| id:ukb-b-11842 | covid_vs_pop | Weighted mode | 480 | 0.03533557 | 0.08994784 | 0.69460869 |
| Whole body fat mass \|\| id:ukb-b-19393 | covid_vs_pop | MR Egger | 413 | 0.15253554 | 0.08999955 | 0.09086176 |
| Whole body fat mass \|\| id:ukb-b-19393 | covid_vs_pop | Weighted median | 413 | 0.19017009 | 0.05361653 | 0.00038986 |
| Whole body fat mass \|\| id:ukb-b-19393 | covid_vs_pop | Inverse variance weighted | 413 | 0.19152607 | 0.03231696 | 3.09E-09 |
| Whole body fat mass \|\| id:ukb-b-19393 | covid_vs_pop | Simple mode | 413 | 0.04651664 | 0.16184656 | 0.77394138 |
| Whole body fat mass \|\| id:ukb-b-19393 | covid_vs_pop | Weighted mode | 413 | 0.12322244 | 0.09531056 | 0.19678679 |
| Whole body fat-free mass \|\| id:ukb-b-13354 | covid_vs_pop | MR Egger | 529 | 0.12058806 | 0.0899376 | 0.18056404 |
| Whole body fat-free mass \|\| id:ukb-b-13354 | covid_vs_pop | Weighted median | 529 | 0.09888177 | 0.05721617 | 0.08394988 |
| Whole body fat-free mass \|\| id:ukb-b-13354 | covid_vs_pop | Inverse variance weighted | 529 | 0.15402575 | 0.03740357 | 3.82E-05 |
| Whole body fat-free mass \|\| id:ukb-b-13354 | covid_vs_pop | Simple mode | 529 | -0.0104264 | 0.17379409 | 0.95218407 |
| Whole body fat-free mass \|\| id:ukb-b-13354 | covid_vs_pop | Weighted mode | 529 | -0.0104264 | 0.13189152 | 0.93702058 |
| bmi | covid_vs_pop | MR Egger | 513 | 0.27060027 | 0.08374781 | 0.00131247 |
| bmi | covid_vs_pop | Weighted median | 513 | 0.21986331 | 0.0515062 | 1.97E-05 |
| bmi | covid_vs_pop | Inverse variance weighted | 513 | 0.18626061 | 0.03141912 | 3.06E-09 |
| bmi | covid_vs_pop | Simple mode | 513 | 0.27284959 | 0.15975507 | 0.08825652 |
| bmi | covid_vs_pop | Weighted mode | 513 | 0.17320578 | 0.10016501 | 0.08437527 |
| height | covid_vs_pop | MR Egger | 814 | 0.0851541 | 0.03906255 | 0.02954833 |
| height | covid_vs_pop | Weighted median | 814 | 0.03976643 | 0.02778445 | 0.15235922 |
| height | covid_vs_pop | Inverse variance weighted | 814 | 0.05619844 | 0.01830612 | 0.00214111 |
| height | covid_vs_pop | Simple mode | 814 | 0.01876375 | 0.08221942 | 0.81953603 |
| height | covid_vs_pop | Weighted mode | 814 | 0.04772318 | 0.05500926 | 0.38589779 |
| interleukin 6 receptor \|\| id:prot-b-23 | covid_vs_pop | Inverse variance weighted | 2 | -0.0124172 | 0.01207607 | 0.30383418 |
| WHRadj | covid_vs_pop | MR Egger | 316 | 0.12395314 | 0.08403574 | 0.14121315 |
| WHRadj | covid_vs_pop | Weighted median | 316 | 0.00241077 | 0.05781667 | 0.96674042 |
| WHRadj | covid_vs_pop | Inverse variance weighted | 316 | -0.0079623 | 0.0352495 | 0.82129073 |
| WHRadj | covid_vs_pop | Simple mode | 316 | -0.1407667 | 0.15809812 | 0.37394373 |
| WHRadj | covid_vs_pop | Weighted mode | 316 | -0.0169382 | 0.09374092 | 0.85672622 |
| Adiponectin | hcovid_vs_pop | MR Egger | 17 | -0.0424792 | 0.10679369 | 0.69640411 |
| Adiponectin | hcovid_vs_pop | Weighted median | 17 | -0.0426821 | 0.07367878 | 0.56238708 |
| Adiponectin | hcovid_vs_pop | Inverse variance weighted | 17 | -0.0314742 | 0.06704894 | 0.6387688 |
| Adiponectin | hcovid_vs_pop | Simple mode | 17 | 0.01958562 | 0.12525139 | 0.87769701 |
| Adiponectin | hcovid_vs_pop | Weighted mode | 17 | -0.0401738 | 0.06948267 | 0.57118878 |
| Alanine aminotransferase \|\| id:ukb-d-30620_irnt | hcovid_vs_pop | MR Egger | 179 | -0.3399562 | 0.14776831 | 0.02258029 |
| Alanine aminotransferase \|\| id:ukb-d-30620_irnt | hcovid_vs_pop | Weighted median | 179 | -0.1036224 | 0.11427512 | 0.36452319 |
| Alanine aminotransferase \|\| id:ukb-d-30620_irnt | hcovid_vs_pop | Inverse variance weighted | 179 | 0.02686631 | 0.07413435 | 0.7170529 |
| Alanine aminotransferase \|\| id:ukb-d-30620_irnt | hcovid_vs_pop | Simple mode | 179 | -0.205247 | 0.314023 | 0.51420983 |
| Alanine aminotransferase \|\| id:ukb-d-30620_irnt | hcovid_vs_pop | Weighted mode | 179 | -0.258599 | 0.21776742 | 0.23661235 |
| Albumin \|\| id:ukb-d-30600_irnt | hcovid_vs_pop | MR Egger | 184 | -0.0709094 | 0.14126559 | 0.61630393 |
| Albumin \|\| id:ukb-d-30600_irnt | hcovid_vs_pop | Weighted median | 184 | -0.1885807 | 0.11204387 | 0.09235628 |
| Albumin \|\| id:ukb-d-30600_irnt | hcovid_vs_pop | Inverse variance weighted | 184 | -0.1640969 | 0.07238366 | 0.02338755 |
| Albumin \|\| id:ukb-d-30600_irnt | hcovid_vs_pop | Simple mode | 184 | -0.1621878 | 0.24764024 | 0.5133331 |
| Albumin \|\| id:ukb-d-30600_irnt | hcovid_vs_pop | Weighted mode | 184 | -0.200968 | 0.15993871 | 0.21052488 |
| Alkaline phosphatase \|\| id:ukb-d-30610_irnt | hcovid_vs_pop | MR Egger | 278 | 0.00882262 | 0.08231441 | 0.91472249 |
| Alkaline phosphatase \|\| id:ukb-d-30610_irnt | hcovid_vs_pop | Weighted median | 278 | 0.03763521 | 0.07640823 | 0.62232745 |
| Alkaline phosphatase \|\| id:ukb-d-30610_irnt | hcovid_vs_pop | Inverse variance weighted | 278 | 0.08619447 | 0.04943362 | 0.08122178 |
| Alkaline phosphatase \|\| id:ukb-d-30610_irnt | hcovid_vs_pop | Simple mode | 278 | 0.10248644 | 0.16170894 | 0.52675321 |
| Alkaline phosphatase \|\| id:ukb-d-30610_irnt | hcovid_vs_pop | Weighted mode | 278 | 0.00094559 | 0.08477214 | 0.99110823 |
| Apoliprotein A \|\| id:ukb-d-30630_irnt | hcovid_vs_pop | MR Egger | 230 | 0.07948497 | 0.08689851 | 0.36132207 |
| Apoliprotein A \|\| id:ukb-d-30630_irnt | hcovid_vs_pop | Weighted median | 230 | 0.0829046 | 0.08009229 | 0.30061601 |
| Apoliprotein A \|\| id:ukb-d-30630_irnt | hcovid_vs_pop | Inverse variance weighted | 230 | 0.00654626 | 0.05251161 | 0.90079027 |
| Apoliprotein A \|\| id:ukb-d-30630_irnt | hcovid_vs_pop | Simple mode | 230 | 0.00213509 | 0.17372116 | 0.99020468 |
| Apoliprotein A \|\| id:ukb-d-30630_irnt | hcovid_vs_pop | Weighted mode | 230 | 0.14980742 | 0.08469975 | 0.07827719 |
| Apolipoprotein B \|\| id:ukb-d-30640_irnt | hcovid_vs_pop | MR Egger | 143 | 0.04235335 | 0.10272539 | 0.6807481 |
| Apolipoprotein B \|\| id:ukb-d-30640_irnt | hcovid_vs_pop | Weighted median | 143 | -0.0255274 | 0.09112322 | 0.77936894 |
| Apolipoprotein B \|\| id:ukb-d-30640_irnt | hcovid_vs_pop | Inverse variance weighted | 143 | 0.00643839 | 0.06414427 | 0.92004774 |
| Apolipoprotein B \|\| id:ukb-d-30640_irnt | hcovid_vs_pop | Simple mode | 143 | -0.1262112 | 0.17666015 | 0.47613435 |
| Apolipoprotein B \|\| id:ukb-d-30640_irnt | hcovid_vs_pop | Weighted mode | 143 | -0.0319233 | 0.09762494 | 0.74414996 |
| Arm fat mass (left) \|\| id:ukb-b-8338 | hcovid_vs_pop | MR Egger | 404 | 0.37108331 | 0.17025253 | 0.02986646 |
| Arm fat mass (left) \|\| id:ukb-b-8338 | hcovid_vs_pop | Weighted median | 404 | 0.28852163 | 0.11076729 | 0.009194 |
| Arm fat mass (left) \|\| id:ukb-b-8338 | hcovid_vs_pop | Inverse variance weighted | 404 | 0.39009057 | 0.06265131 | 4.77E-10 |
| Arm fat mass (left) \|\| id:ukb-b-8338 | hcovid_vs_pop | Simple mode | 404 | 0.50171466 | 0.30103022 | 0.09635922 |
| Arm fat mass (left) \|\| id:ukb-b-8338 | hcovid_vs_pop | Weighted mode | 404 | 0.26512813 | 0.18979844 | 0.16321446 |
| Arm fat mass (right) \|\| id:ukb-b-6704 | hcovid_vs_pop | MR Egger | 410 | 0.31598805 | 0.16568261 | 0.0571981 |
| Arm fat mass (right) \|\| id:ukb-b-6704 | hcovid_vs_pop | Weighted median | 410 | 0.29457975 | 0.10969607 | 0.00724392 |
| Arm fat mass (right) \|\| id:ukb-b-6704 | hcovid_vs_pop | Inverse variance weighted | 410 | 0.42991315 | 0.06188074 | 3.72E-12 |
| Arm fat mass (right) \|\| id:ukb-b-6704 | hcovid_vs_pop | Simple mode | 410 | 0.63362635 | 0.31563454 | 0.04535717 |
| Arm fat mass (right) \|\| id:ukb-b-6704 | hcovid_vs_pop | Weighted mode | 410 | 0.20277198 | 0.20934664 | 0.33332003 |
| Arm fat percentage (left) \|\| id:ukb-b-20188 | hcovid_vs_pop | MR Egger | 379 | 0.53135614 | 0.26422549 | 0.04503743 |
| Arm fat percentage (left) \|\| id:ukb-b-20188 | hcovid_vs_pop | Weighted median | 379 | 0.49663392 | 0.15081545 | 0.00099128 |
| Arm fat percentage (left) \|\| id:ukb-b-20188 | hcovid_vs_pop | Inverse variance weighted | 379 | 0.59739911 | 0.0903146 | 3.72E-11 |
| Arm fat percentage (left) \|\| id:ukb-b-20188 | hcovid_vs_pop | Simple mode | 379 | 0.69390785 | 0.43703265 | 0.11317417 |
| Arm fat percentage (left) \|\| id:ukb-b-20188 | hcovid_vs_pop | Weighted mode | 379 | 0.41000221 | 0.27908433 | 0.14263795 |
| Arm fat percentage (right) \|\| id:ukb-b-12854 | hcovid_vs_pop | MR Egger | 377 | 0.56259254 | 0.26639623 | 0.03535902 |
| Arm fat percentage (right) \|\| id:ukb-b-12854 | hcovid_vs_pop | Weighted median | 377 | 0.4932453 | 0.14853196 | 0.00089759 |
| Arm fat percentage (right) \|\| id:ukb-b-12854 | hcovid_vs_pop | Inverse variance weighted | 377 | 0.63050578 | 0.09170691 | 6.19E-12 |
| Arm fat percentage (right) \|\| id:ukb-b-12854 | hcovid_vs_pop | Simple mode | 377 | 0.67338062 | 0.43081776 | 0.11888701 |
| Arm fat percentage (right) \|\| id:ukb-b-12854 | hcovid_vs_pop | Weighted mode | 377 | 0.38653873 | 0.28735407 | 0.17938259 |
| Arm fat-free mass (left) \|\| id:ukb-b-19925 | hcovid_vs_pop | MR Egger | 493 | 0.06689413 | 0.21319026 | 0.75382391 |
| Arm fat-free mass (left) \|\| id:ukb-b-19925 | hcovid_vs_pop | Weighted median | 493 | 0.17317744 | 0.13292544 | 0.19263746 |
| Arm fat-free mass (left) \|\| id:ukb-b-19925 | hcovid_vs_pop | Inverse variance weighted | 493 | 0.31134198 | 0.08383595 | 0.00020425 |
| Arm fat-free mass (left) \|\| id:ukb-b-19925 | hcovid_vs_pop | Simple mode | 493 | -0.0315003 | 0.46545498 | 0.94607074 |
| Arm fat-free mass (left) \|\| id:ukb-b-19925 | hcovid_vs_pop | Weighted mode | 493 | -0.0315003 | 0.29265386 | 0.91432772 |
| Arm fat-free mass (right) \|\| id:ukb-b-19520 | hcovid_vs_pop | MR Egger | 493 | 0.00574824 | 0.21207025 | 0.97838671 |
| Arm fat-free mass (right) \|\| id:ukb-b-19520 | hcovid_vs_pop | Weighted median | 493 | 0.15915622 | 0.13264134 | 0.23017857 |
| Arm fat-free mass (right) \|\| id:ukb-b-19520 | hcovid_vs_pop | Inverse variance weighted | 493 | 0.28119483 | 0.08278936 | 0.00068251 |
| Arm fat-free mass (right) \|\| id:ukb-b-19520 | hcovid_vs_pop | Simple mode | 493 | -0.0777072 | 0.45137748 | 0.86338584 |
| Arm fat-free mass (right) \|\| id:ukb-b-19520 | hcovid_vs_pop | Weighted mode | 493 | -0.1118242 | 0.32050863 | 0.72731664 |
| Aspartate aminotransferase \|\| id:ukb-d-30650_irnt | hcovid_vs_pop | MR Egger | 211 | -0.1181497 | 0.14889385 | 0.42837703 |
| Aspartate aminotransferase \|\| id:ukb-d-30650_irnt | hcovid_vs_pop | Weighted median | 211 | -0.0600358 | 0.10643028 | 0.57269554 |
| Aspartate aminotransferase \|\| id:ukb-d-30650_irnt | hcovid_vs_pop | Inverse variance weighted | 211 | -0.1084069 | 0.06983842 | 0.12060176 |
| Aspartate aminotransferase \|\| id:ukb-d-30650_irnt | hcovid_vs_pop | Simple mode | 211 | -0.1952409 | 0.28784558 | 0.49833877 |
| Aspartate aminotransferase \|\| id:ukb-d-30650_irnt | hcovid_vs_pop | Weighted mode | 211 | 0.06868021 | 0.20367839 | 0.73630345 |
| Body fat percentage \|\| id:ukb-b-8909 | hcovid_vs_pop | MR Egger | 373 | 0.80661995 | 0.29107013 | 0.00586589 |
| Body fat percentage \|\| id:ukb-b-8909 | hcovid_vs_pop | Weighted median | 373 | 0.59436195 | 0.14162145 | 2.71E-05 |
| Body fat percentage \|\| id:ukb-b-8909 | hcovid_vs_pop | Inverse variance weighted | 373 | 0.63606844 | 0.09208328 | 4.93E-12 |
| Body fat percentage \|\| id:ukb-b-8909 | hcovid_vs_pop | Simple mode | 373 | 0.74171226 | 0.4916774 | 0.13226662 |
| Body fat percentage \|\| id:ukb-b-8909 | hcovid_vs_pop | Weighted mode | 373 | 0.44117023 | 0.29917748 | 0.1411631 |
| C-reactive protein \|\| id:ukb-d-30710_irnt | hcovid_vs_pop | MR Egger | 185 | 0.03027163 | 0.10168516 | 0.76627019 |
| C-reactive protein \|\| id:ukb-d-30710_irnt | hcovid_vs_pop | Weighted median | 185 | 0.1122889 | 0.09281088 | 0.2263296 |
| C-reactive protein \|\| id:ukb-d-30710_irnt | hcovid_vs_pop | Inverse variance weighted | 185 | 0.12816764 | 0.06001646 | 0.03271682 |
| C-reactive protein \|\| id:ukb-d-30710_irnt | hcovid_vs_pop | Simple mode | 185 | 0.20471531 | 0.20923348 | 0.32915816 |
| C-reactive protein \|\| id:ukb-d-30710_irnt | hcovid_vs_pop | Weighted mode | 185 | 0.09120685 | 0.08997007 | 0.31203486 |
| Calcium \|\| id:ukb-d-30680_irnt | hcovid_vs_pop | MR Egger | 190 | -0.0485738 | 0.16414172 | 0.76761338 |
| Calcium \|\| id:ukb-d-30680_irnt | hcovid_vs_pop | Weighted median | 190 | 0.10583894 | 0.10342396 | 0.30614224 |
| Calcium \|\| id:ukb-d-30680_irnt | hcovid_vs_pop | Inverse variance weighted | 190 | 0.0721267 | 0.0723767 | 0.318985 |
| Calcium \|\| id:ukb-d-30680_irnt | hcovid_vs_pop | Simple mode | 190 | 0.20393029 | 0.28509752 | 0.47530632 |
| Calcium \|\| id:ukb-d-30680_irnt | hcovid_vs_pop | Weighted mode | 190 | 0.26555155 | 0.25259046 | 0.29445733 |
| Cholesterol \|\| id:ukb-d-30690_irnt | hcovid_vs_pop | MR Egger | 161 | 0.07052149 | 0.10427469 | 0.49982961 |
| Cholesterol \|\| id:ukb-d-30690_irnt | hcovid_vs_pop | Weighted median | 161 | 0.02511654 | 0.09175694 | 0.78429285 |
| Cholesterol \|\| id:ukb-d-30690_irnt | hcovid_vs_pop | Inverse variance weighted | 161 | 0.02305362 | 0.06269912 | 0.71310702 |
| Cholesterol \|\| id:ukb-d-30690_irnt | hcovid_vs_pop | Simple mode | 161 | -0.1248191 | 0.16550833 | 0.45186415 |
| Cholesterol \|\| id:ukb-d-30690_irnt | hcovid_vs_pop | Weighted mode | 161 | -0.0659034 | 0.09644904 | 0.49540697 |
| Creatinine \|\| id:ukb-d-30700_irnt | hcovid_vs_pop | MR Egger | 302 | -0.1050264 | 0.15617934 | 0.50180011 |
| Creatinine \|\| id:ukb-d-30700_irnt | hcovid_vs_pop | Weighted median | 302 | 0.00335012 | 0.10222545 | 0.97385647 |
| Creatinine \|\| id:ukb-d-30700_irnt | hcovid_vs_pop | Inverse variance weighted | 302 | 0.07706059 | 0.06719141 | 0.25143054 |
| Creatinine \|\| id:ukb-d-30700_irnt | hcovid_vs_pop | Simple mode | 302 | -0.2811364 | 0.29185488 | 0.33618302 |
| Creatinine \|\| id:ukb-d-30700_irnt | hcovid_vs_pop | Weighted mode | 302 | -0.1418113 | 0.20071556 | 0.48040598 |
| Cystatin C \|\| id:ukb-d-30720_irnt | hcovid_vs_pop | MR Egger | 284 | -0.3453217 | 0.12204814 | 0.00499853 |
| Cystatin C \|\| id:ukb-d-30720_irnt | hcovid_vs_pop | Weighted median | 284 | -0.1257058 | 0.09868019 | 0.20270934 |
| Cystatin C \|\| id:ukb-d-30720_irnt | hcovid_vs_pop | Inverse variance weighted | 284 | -0.0322468 | 0.06094612 | 0.59673329 |
| Cystatin C \|\| id:ukb-d-30720_irnt | hcovid_vs_pop | Simple mode | 284 | -0.072872 | 0.22607302 | 0.74743431 |
| Cystatin C \|\| id:ukb-d-30720_irnt | hcovid_vs_pop | Weighted mode | 284 | -0.1404388 | 0.12620519 | 0.26674668 |
| Diastolic blood pressure, automated reading \|\| id:ukb-b-7992 | hcovid_vs_pop | MR Egger | 243 | -0.1078022 | 0.26590619 | 0.68553254 |
| Diastolic blood pressure, automated reading \|\| id:ukb-b-7992 | hcovid_vs_pop | Weighted median | 243 | -0.2026827 | 0.12287162 | 0.09903525 |
| Diastolic blood pressure, automated reading \|\| id:ukb-b-7992 | hcovid_vs_pop | Inverse variance weighted | 243 | -0.1334439 | 0.08643446 | 0.12261899 |
| Diastolic blood pressure, automated reading \|\| id:ukb-b-7992 | hcovid_vs_pop | Simple mode | 243 | -0.4521001 | 0.40945663 | 0.27062626 |
| Diastolic blood pressure, automated reading \|\| id:ukb-b-7992 | hcovid_vs_pop | Weighted mode | 243 | -0.4919613 | 0.37917867 | 0.19571552 |
| Direct bilirubin \|\| id:ukb-d-30660_irnt | hcovid_vs_pop | MR Egger | 72 | -0.0362671 | 0.10041976 | 0.71907149 |
| Direct bilirubin \|\| id:ukb-d-30660_irnt | hcovid_vs_pop | Weighted median | 72 | -0.0644335 | 0.10801644 | 0.55083059 |
| Direct bilirubin \|\| id:ukb-d-30660_irnt | hcovid_vs_pop | Inverse variance weighted | 72 | 0.00782317 | 0.07502887 | 0.916956 |
| Direct bilirubin \|\| id:ukb-d-30660_irnt | hcovid_vs_pop | Simple mode | 72 | -0.114932 | 0.21041367 | 0.58662746 |
| Direct bilirubin \|\| id:ukb-d-30660_irnt | hcovid_vs_pop | Weighted mode | 72 | -0.1326546 | 0.10298499 | 0.20189458 |
| Gamma glutamyltransferase \|\| id:ukb-d-30730_irnt | hcovid_vs_pop | MR Egger | 251 | 0.02712718 | 0.09634946 | 0.77852251 |
| Gamma glutamyltransferase \|\| id:ukb-d-30730_irnt | hcovid_vs_pop | Weighted median | 251 | 0.02154447 | 0.07957017 | 0.7865751 |
| Gamma glutamyltransferase \|\| id:ukb-d-30730_irnt | hcovid_vs_pop | Inverse variance weighted | 251 | 0.01640795 | 0.05334934 | 0.75841971 |
| Gamma glutamyltransferase \|\| id:ukb-d-30730_irnt | hcovid_vs_pop | Simple mode | 251 | -0.084201 | 0.16516168 | 0.61063457 |
| Gamma glutamyltransferase \|\| id:ukb-d-30730_irnt | hcovid_vs_pop | Weighted mode | 251 | -0.0001258 | 0.08916851 | 0.99887548 |
| Glucose \|\| id:ukb-d-30740_irnt | hcovid_vs_pop | MR Egger | 102 | -0.0329304 | 0.1670223 | 0.84410126 |
| Glucose \|\| id:ukb-d-30740_irnt | hcovid_vs_pop | Weighted median | 102 | -0.0245116 | 0.12485194 | 0.84435568 |
| Glucose \|\| id:ukb-d-30740_irnt | hcovid_vs_pop | Inverse variance weighted | 102 | -0.012243 | 0.08866587 | 0.89017681 |
| Glucose \|\| id:ukb-d-30740_irnt | hcovid_vs_pop | Simple mode | 102 | 0.1149093 | 0.25072555 | 0.64771643 |
| Glucose \|\| id:ukb-d-30740_irnt | hcovid_vs_pop | Weighted mode | 102 | -0.0167838 | 0.11920724 | 0.8883119 |
| Glycated haemoglobin \|\| id:ukb-d-30750_irnt | hcovid_vs_pop | MR Egger | 273 | 0.0067105 | 0.09659294 | 0.94466522 |
| Glycated haemoglobin \|\| id:ukb-d-30750_irnt | hcovid_vs_pop | Weighted median | 273 | 0.01071091 | 0.07635768 | 0.88844445 |
| Glycated haemoglobin \|\| id:ukb-d-30750_irnt | hcovid_vs_pop | Inverse variance weighted | 273 | 0.07367894 | 0.04883749 | 0.13138687 |
| Glycated haemoglobin \|\| id:ukb-d-30750_irnt | hcovid_vs_pop | Simple mode | 273 | 0.18187918 | 0.18161638 | 0.31750082 |
| Glycated haemoglobin \|\| id:ukb-d-30750_irnt | hcovid_vs_pop | Weighted mode | 273 | 0.02978903 | 0.0899868 | 0.74087081 |
| HDL cholesterol \|\| id:ukb-d-30760_irnt | hcovid_vs_pop | MR Egger | 250 | -0.0465274 | 0.0825766 | 0.57364083 |
| HDL cholesterol \|\| id:ukb-d-30760_irnt | hcovid_vs_pop | Weighted median | 250 | -0.0230477 | 0.07857493 | 0.76927632 |
| HDL cholesterol \|\| id:ukb-d-30760_irnt | hcovid_vs_pop | Inverse variance weighted | 250 | -0.0559263 | 0.04936119 | 0.25721388 |
| HDL cholesterol \|\| id:ukb-d-30760_irnt | hcovid_vs_pop | Simple mode | 250 | 0.02805101 | 0.16468163 | 0.86488516 |
| HDL cholesterol \|\| id:ukb-d-30760_irnt | hcovid_vs_pop | Weighted mode | 250 | 0.00725613 | 0.0858216 | 0.93268792 |
| Hip circumference \|\| id:ukb-b-15590 | hcovid_vs_pop | MR Egger | 399 | 0.19670836 | 0.17478278 | 0.26108055 |
| Hip circumference \|\| id:ukb-b-15590 | hcovid_vs_pop | Weighted median | 399 | 0.271907 | 0.10743219 | 0.01137495 |
| Hip circumference \|\| id:ukb-b-15590 | hcovid_vs_pop | Inverse variance weighted | 399 | 0.33813679 | 0.06415784 | 1.36E-07 |
| Hip circumference \|\| id:ukb-b-15590 | hcovid_vs_pop | Simple mode | 399 | 0.40008455 | 0.30236898 | 0.1865396 |
| Hip circumference \|\| id:ukb-b-15590 | hcovid_vs_pop | Weighted mode | 399 | 0.26806704 | 0.21272591 | 0.20835279 |
| IGF-1 \|\| id:ukb-d-30770_irnt | hcovid_vs_pop | MR Egger | 316 | -0.1283468 | 0.10917948 | 0.24066223 |
| IGF-1 \|\| id:ukb-d-30770_irnt | hcovid_vs_pop | Weighted median | 316 | -0.070669 | 0.07925838 | 0.37259251 |
| IGF-1 \|\| id:ukb-d-30770_irnt | hcovid_vs_pop | Inverse variance weighted | 316 | -0.0688943 | 0.05388201 | 0.20103305 |
| IGF-1 \|\| id:ukb-d-30770_irnt | hcovid_vs_pop | Simple mode | 316 | -0.2128812 | 0.20575585 | 0.3016353 |
| IGF-1 \|\| id:ukb-d-30770_irnt | hcovid_vs_pop | Weighted mode | 316 | -0.1423037 | 0.11154561 | 0.20298585 |
| Insulin Resistance | hcovid_vs_pop | MR Egger | 48 | -0.0833606 | 0.27203605 | 0.76065838 |
| Insulin Resistance | hcovid_vs_pop | Weighted median | 48 | -0.0562695 | 0.23261802 | 0.80886024 |
| Insulin Resistance | hcovid_vs_pop | Inverse variance weighted | 48 | 0.02048538 | 0.1411308 | 0.88459109 |
| Insulin Resistance | hcovid_vs_pop | Simple mode | 48 | 0.13332019 | 0.3636468 | 0.71554786 |
| Insulin Resistance | hcovid_vs_pop | Weighted mode | 48 | -0.0180104 | 0.22062035 | 0.93528348 |
| LDL direct \|\| id:ukb-d-30780_irnt | hcovid_vs_pop | MR Egger | 140 | 0.06568375 | 0.11132887 | 0.55615721 |
| LDL direct \|\| id:ukb-d-30780_irnt | hcovid_vs_pop | Weighted median | 140 | 0.08372201 | 0.09319604 | 0.36900268 |
| LDL direct \|\| id:ukb-d-30780_irnt | hcovid_vs_pop | Inverse variance weighted | 140 | 0.06207915 | 0.06873481 | 0.36643621 |
| LDL direct \|\| id:ukb-d-30780_irnt | hcovid_vs_pop | Simple mode | 140 | -0.1266317 | 0.18009725 | 0.48315114 |
| LDL direct \|\| id:ukb-d-30780_irnt | hcovid_vs_pop | Weighted mode | 140 | 0.00767156 | 0.09492271 | 0.93570216 |
| Leg fat mass (left) \|\| id:ukb-b-7212 | hcovid_vs_pop | MR Egger | 402 | 0.50248366 | 0.2165061 | 0.02079561 |
| Leg fat mass (left) \|\| id:ukb-b-7212 | hcovid_vs_pop | Weighted median | 402 | 0.46717908 | 0.13134117 | 0.00037513 |
| Leg fat mass (left) \|\| id:ukb-b-7212 | hcovid_vs_pop | Inverse variance weighted | 402 | 0.55309732 | 0.07803688 | 1.36E-12 |
| Leg fat mass (left) \|\| id:ukb-b-7212 | hcovid_vs_pop | Simple mode | 402 | 0.5784068 | 0.36920746 | 0.11799292 |
| Leg fat mass (left) \|\| id:ukb-b-7212 | hcovid_vs_pop | Weighted mode | 402 | 0.39876256 | 0.23458352 | 0.08992996 |
| Leg fat mass (right) \|\| id:ukb-b-18096 | hcovid_vs_pop | MR Egger | 407 | 0.47061484 | 0.21735437 | 0.03095596 |
| Leg fat mass (right) \|\| id:ukb-b-18096 | hcovid_vs_pop | Weighted median | 407 | 0.52353384 | 0.13893383 | 0.00016441 |
| Leg fat mass (right) \|\| id:ukb-b-18096 | hcovid_vs_pop | Inverse variance weighted | 407 | 0.61193957 | 0.07788087 | 3.92E-15 |
| Leg fat mass (right) \|\| id:ukb-b-18096 | hcovid_vs_pop | Simple mode | 407 | 0.8062099 | 0.39104336 | 0.03987333 |
| Leg fat mass (right) \|\| id:ukb-b-18096 | hcovid_vs_pop | Weighted mode | 407 | 0.40628637 | 0.24800198 | 0.10214667 |
| Leg fat percentage (left) \|\| id:ukb-b-18377 | hcovid_vs_pop | MR Egger | 360 | 0.85337649 | 0.35414293 | 0.01646976 |
| Leg fat percentage (left) \|\| id:ukb-b-18377 | hcovid_vs_pop | Weighted median | 360 | 0.76291383 | 0.18199277 | 2.77E-05 |
| Leg fat percentage (left) \|\| id:ukb-b-18377 | hcovid_vs_pop | Inverse variance weighted | 360 | 0.85762822 | 0.10907654 | 3.76E-15 |
| Leg fat percentage (left) \|\| id:ukb-b-18377 | hcovid_vs_pop | Simple mode | 360 | 1.19242578 | 0.58549885 | 0.0424237 |
| Leg fat percentage (left) \|\| id:ukb-b-18377 | hcovid_vs_pop | Weighted mode | 360 | 0.6632475 | 0.39847901 | 0.09689549 |
| Leg fat percentage (right) \|\| id:ukb-b-20531 | hcovid_vs_pop | MR Egger | 365 | 0.70635132 | 0.35490632 | 0.04731335 |
| Leg fat percentage (right) \|\| id:ukb-b-20531 | hcovid_vs_pop | Weighted median | 365 | 0.67710552 | 0.17985192 | 0.00016669 |
| Leg fat percentage (right) \|\| id:ukb-b-20531 | hcovid_vs_pop | Inverse variance weighted | 365 | 0.85410533 | 0.11088122 | 1.33E-14 |
| Leg fat percentage (right) \|\| id:ukb-b-20531 | hcovid_vs_pop | Simple mode | 365 | 0.87147725 | 0.52321727 | 0.09665095 |
| Leg fat percentage (right) \|\| id:ukb-b-20531 | hcovid_vs_pop | Weighted mode | 365 | 0.5560135 | 0.3838299 | 0.14831199 |
| Leg fat-free mass (left) \|\| id:ukb-b-16099 | hcovid_vs_pop | MR Egger | 485 | 0.23661682 | 0.19407224 | 0.22335565 |
| Leg fat-free mass (left) \|\| id:ukb-b-16099 | hcovid_vs_pop | Weighted median | 485 | 0.11716332 | 0.12924891 | 0.36467459 |
| Leg fat-free mass (left) \|\| id:ukb-b-16099 | hcovid_vs_pop | Inverse variance weighted | 485 | 0.21463205 | 0.07898065 | 0.00657718 |
| Leg fat-free mass (left) \|\| id:ukb-b-16099 | hcovid_vs_pop | Simple mode | 485 | -0.1302676 | 0.38273353 | 0.73373236 |
| Leg fat-free mass (left) \|\| id:ukb-b-16099 | hcovid_vs_pop | Weighted mode | 485 | -0.0945275 | 0.25000675 | 0.70552229 |
| Leg fat-free mass (right) \|\| id:ukb-b-12828 | hcovid_vs_pop | MR Egger | 489 | 0.10329901 | 0.19145115 | 0.58974846 |
| Leg fat-free mass (right) \|\| id:ukb-b-12828 | hcovid_vs_pop | Weighted median | 489 | 0.06757479 | 0.12407547 | 0.58601043 |
| Leg fat-free mass (right) \|\| id:ukb-b-12828 | hcovid_vs_pop | Inverse variance weighted | 489 | 0.1543161 | 0.07751739 | 0.04651071 |
| Leg fat-free mass (right) \|\| id:ukb-b-12828 | hcovid_vs_pop | Simple mode | 489 | -0.0386905 | 0.43066292 | 0.92845161 |
| Leg fat-free mass (right) \|\| id:ukb-b-12828 | hcovid_vs_pop | Weighted mode | 489 | -0.0999329 | 0.26627553 | 0.70760132 |
| Lipoprotein A \|\| id:ukb-d-30790_irnt | hcovid_vs_pop | MR Egger | 13 | 0.1379259 | 0.22471903 | 0.55184833 |
| Lipoprotein A \|\| id:ukb-d-30790_irnt | hcovid_vs_pop | Weighted median | 13 | 0.02918633 | 0.1393796 | 0.83413466 |
| Lipoprotein A \|\| id:ukb-d-30790_irnt | hcovid_vs_pop | Inverse variance weighted | 13 | -0.0695947 | 0.13661784 | 0.61046373 |
| Lipoprotein A \|\| id:ukb-d-30790_irnt | hcovid_vs_pop | Simple mode | 13 | 0.17069929 | 0.21474972 | 0.44212404 |
| Lipoprotein A \|\| id:ukb-d-30790_irnt | hcovid_vs_pop | Weighted mode | 13 | 0.24024632 | 0.24173457 | 0.33991733 |
| Oestradiol \|\| id:ukb-d-30800_irnt | hcovid_vs_pop | Wald ratio | 1 | -0.1386291 | 0.61464088 | 0.82155547 |
| Phosphate \|\| id:ukb-d-30810_irnt | hcovid_vs_pop | MR Egger | 136 | -0.1173295 | 0.15125046 | 0.43927541 |
| Phosphate \|\| id:ukb-d-30810_irnt | hcovid_vs_pop | Weighted median | 136 | -0.1975974 | 0.11752273 | 0.09269406 |
| Phosphate \|\| id:ukb-d-30810_irnt | hcovid_vs_pop | Inverse variance weighted | 136 | -0.1107339 | 0.08039007 | 0.16837101 |
| Phosphate \|\| id:ukb-d-30810_irnt | hcovid_vs_pop | Simple mode | 136 | -0.3641847 | 0.2331326 | 0.12059727 |
| Phosphate \|\| id:ukb-d-30810_irnt | hcovid_vs_pop | Weighted mode | 136 | -0.2761946 | 0.12772095 | 0.03234297 |
| SHBG \|\| id:ukb-d-30830_irnt | hcovid_vs_pop | MR Egger | 235 | 0.13408807 | 0.10392674 | 0.19825424 |
| SHBG \|\| id:ukb-d-30830_irnt | hcovid_vs_pop | Weighted median | 235 | 0.05886298 | 0.08631146 | 0.49524944 |
| SHBG \|\| id:ukb-d-30830_irnt | hcovid_vs_pop | Inverse variance weighted | 235 | 0.0246719 | 0.05804881 | 0.67082219 |
| SHBG \|\| id:ukb-d-30830_irnt | hcovid_vs_pop | Simple mode | 235 | 0.14423343 | 0.17578367 | 0.41275668 |
| SHBG \|\| id:ukb-d-30830_irnt | hcovid_vs_pop | Weighted mode | 235 | 0.07546877 | 0.11017195 | 0.49401627 |
| Systolic blood pressure, automated reading \|\| id:ukb-b-20175 | hcovid_vs_pop | MR Egger | 231 | 0.39946505 | 0.25938815 | 0.12493414 |
| Systolic blood pressure, automated reading \|\| id:ukb-b-20175 | hcovid_vs_pop | Weighted median | 231 | 0.05552265 | 0.13022466 | 0.66984516 |
| Systolic blood pressure, automated reading \|\| id:ukb-b-20175 | hcovid_vs_pop | Inverse variance weighted | 231 | 0.02294858 | 0.08494944 | 0.78704948 |
| Systolic blood pressure, automated reading \|\| id:ukb-b-20175 | hcovid_vs_pop | Simple mode | 231 | -0.2528917 | 0.37765932 | 0.50376587 |
| Systolic blood pressure, automated reading \|\| id:ukb-b-20175 | hcovid_vs_pop | Weighted mode | 231 | 0.05202086 | 0.30694446 | 0.8655683 |
| Testosterone \|\| id:ukb-d-30850_irnt | hcovid_vs_pop | MR Egger | 88 | -0.5884404 | 0.27786513 | 0.03708556 |
| Testosterone \|\| id:ukb-d-30850_irnt | hcovid_vs_pop | Weighted median | 88 | -0.4051382 | 0.23058609 | 0.07891893 |
| Testosterone \|\| id:ukb-d-30850_irnt | hcovid_vs_pop | Inverse variance weighted | 88 | -0.2260148 | 0.15641333 | 0.14846232 |
| Testosterone \|\| id:ukb-d-30850_irnt | hcovid_vs_pop | Simple mode | 88 | -0.4510153 | 0.57153561 | 0.43218192 |
| Testosterone \|\| id:ukb-d-30850_irnt | hcovid_vs_pop | Weighted mode | 88 | -0.4510153 | 0.25074595 | 0.07553497 |
| Total bilirubin \|\| id:ukb-d-30840_irnt | hcovid_vs_pop | MR Egger | 119 | 0.2682896 | 0.11211762 | 0.01830566 |
| Total bilirubin \|\| id:ukb-d-30840_irnt | hcovid_vs_pop | Weighted median | 119 | 0.15927164 | 0.10696223 | 0.13647536 |
| Total bilirubin \|\| id:ukb-d-30840_irnt | hcovid_vs_pop | Inverse variance weighted | 119 | 0.0978888 | 0.07924276 | 0.21671785 |
| Total bilirubin \|\| id:ukb-d-30840_irnt | hcovid_vs_pop | Simple mode | 119 | -0.0080495 | 0.20231575 | 0.96833014 |
| Total bilirubin \|\| id:ukb-d-30840_irnt | hcovid_vs_pop | Weighted mode | 119 | 0.18097111 | 0.11097209 | 0.10560299 |
| Total protein \|\| id:ukb-d-30860_irnt | hcovid_vs_pop | MR Egger | 210 | -0.0668581 | 0.15033811 | 0.6569859 |
| Total protein \|\| id:ukb-d-30860_irnt | hcovid_vs_pop | Weighted median | 210 | -0.0837987 | 0.10994314 | 0.44594035 |
| Total protein \|\| id:ukb-d-30860_irnt | hcovid_vs_pop | Inverse variance weighted | 210 | -0.0115532 | 0.06549863 | 0.85998891 |
| Total protein \|\| id:ukb-d-30860_irnt | hcovid_vs_pop | Simple mode | 210 | -0.0895207 | 0.2942004 | 0.76121367 |
| Total protein \|\| id:ukb-d-30860_irnt | hcovid_vs_pop | Weighted mode | 210 | -0.1497775 | 0.18407439 | 0.41675465 |
| Triglycerides \|\| id:ukb-d-30870_irnt | hcovid_vs_pop | MR Egger | 210 | 0.17080961 | 0.08294631 | 0.04071305 |
| Triglycerides \|\| id:ukb-d-30870_irnt | hcovid_vs_pop | Weighted median | 210 | 0.1301865 | 0.07850362 | 0.09724681 |
| Triglycerides \|\| id:ukb-d-30870_irnt | hcovid_vs_pop | Inverse variance weighted | 210 | 0.14658909 | 0.05129806 | 0.00426864 |
| Triglycerides \|\| id:ukb-d-30870_irnt | hcovid_vs_pop | Simple mode | 210 | 0.21505342 | 0.14187833 | 0.13109115 |
| Triglycerides \|\| id:ukb-d-30870_irnt | hcovid_vs_pop | Weighted mode | 210 | 0.13784429 | 0.07248702 | 0.05859349 |
| Trunk fat mass \|\| id:ukb-b-20044 | hcovid_vs_pop | MR Egger | 403 | 0.60679241 | 0.17204202 | 0.0004689 |
| Trunk fat mass \|\| id:ukb-b-20044 | hcovid_vs_pop | Weighted median | 403 | 0.34896034 | 0.10231887 | 0.0006484 |
| Trunk fat mass \|\| id:ukb-b-20044 | hcovid_vs_pop | Inverse variance weighted | 403 | 0.40424805 | 0.06166594 | 5.55E-11 |
| Trunk fat mass \|\| id:ukb-b-20044 | hcovid_vs_pop | Simple mode | 403 | 0.37131451 | 0.27455516 | 0.17700053 |
| Trunk fat mass \|\| id:ukb-b-20044 | hcovid_vs_pop | Weighted mode | 403 | 0.32264557 | 0.20007383 | 0.10760909 |
| Trunk fat percentage \|\| id:ukb-b-16407 | hcovid_vs_pop | MR Egger | 370 | 0.93084969 | 0.24116706 | 0.00013402 |
| Trunk fat percentage \|\| id:ukb-b-16407 | hcovid_vs_pop | Weighted median | 370 | 0.47228701 | 0.11236385 | 2.63E-05 |
| Trunk fat percentage \|\| id:ukb-b-16407 | hcovid_vs_pop | Inverse variance weighted | 370 | 0.5351438 | 0.07696821 | 3.58E-12 |
| Trunk fat percentage \|\| id:ukb-b-16407 | hcovid_vs_pop | Simple mode | 370 | 0.46298207 | 0.35891261 | 0.19787319 |
| Trunk fat percentage \|\| id:ukb-b-16407 | hcovid_vs_pop | Weighted mode | 370 | 0.412588 | 0.24154928 | 0.08846059 |
| Trunk fat-free mass \|\| id:ukb-b-17409 | hcovid_vs_pop | MR Egger | 537 | -0.0010143 | 0.17959478 | 0.99549571 |
| Trunk fat-free mass \|\| id:ukb-b-17409 | hcovid_vs_pop | Weighted median | 537 | 0.04197746 | 0.11673644 | 0.71915243 |
| Trunk fat-free mass \|\| id:ukb-b-17409 | hcovid_vs_pop | Inverse variance weighted | 537 | 0.15758966 | 0.07478627 | 0.03510021 |
| Trunk fat-free mass \|\| id:ukb-b-17409 | hcovid_vs_pop | Simple mode | 537 | 0.00502948 | 0.40896017 | 0.99019226 |
| Trunk fat-free mass \|\| id:ukb-b-17409 | hcovid_vs_pop | Weighted mode | 537 | -0.1577408 | 0.25791083 | 0.5410552 |
| Type 2 diabetes \|\| id:ebi-a-GCST006867 | hcovid_vs_pop | MR Egger | 115 | -0.0770703 | 0.06431925 | 0.23332802 |
| Type 2 diabetes \|\| id:ebi-a-GCST006867 | hcovid_vs_pop | Weighted median | 115 | -0.022486 | 0.05390069 | 0.67655115 |
| Type 2 diabetes \|\| id:ebi-a-GCST006867 | hcovid_vs_pop | Inverse variance weighted | 115 | -0.0075129 | 0.02767388 | 0.78602115 |
| Type 2 diabetes \|\| id:ebi-a-GCST006867 | hcovid_vs_pop | Simple mode | 115 | -0.0081463 | 0.09928731 | 0.93475295 |
| Type 2 diabetes \|\| id:ebi-a-GCST006867 | hcovid_vs_pop | Weighted mode | 115 | -0.0285085 | 0.05752202 | 0.62112297 |
| Urate \|\| id:ukb-d-30880_irnt | hcovid_vs_pop | MR Egger | 223 | -0.0946357 | 0.10941573 | 0.38802115 |
| Urate \|\| id:ukb-d-30880_irnt | hcovid_vs_pop | Weighted median | 223 | -0.0899287 | 0.10628153 | 0.39747626 |
| Urate \|\| id:ukb-d-30880_irnt | hcovid_vs_pop | Inverse variance weighted | 223 | 0.0436459 | 0.0667612 | 0.51326552 |
| Urate \|\| id:ukb-d-30880_irnt | hcovid_vs_pop | Simple mode | 223 | -0.1339495 | 0.23447746 | 0.56839585 |
| Urate \|\| id:ukb-d-30880_irnt | hcovid_vs_pop | Weighted mode | 223 | -0.0929673 | 0.11441063 | 0.41733194 |
| Urea \|\| id:ukb-d-30670_irnt | hcovid_vs_pop | MR Egger | 152 | 0.08690474 | 0.19632123 | 0.65864499 |
| Urea \|\| id:ukb-d-30670_irnt | hcovid_vs_pop | Weighted median | 152 | -0.1096679 | 0.11956276 | 0.35901612 |
| Urea \|\| id:ukb-d-30670_irnt | hcovid_vs_pop | Inverse variance weighted | 152 | -0.0394528 | 0.08514576 | 0.64310918 |
| Urea \|\| id:ukb-d-30670_irnt | hcovid_vs_pop | Simple mode | 152 | -0.5706557 | 0.3099203 | 0.06754008 |
| Urea \|\| id:ukb-d-30670_irnt | hcovid_vs_pop | Weighted mode | 152 | -0.0575617 | 0.2180302 | 0.79213391 |
| Vitamin D \|\| id:ukb-d-30890_irnt | hcovid_vs_pop | MR Egger | 54 | -0.0873962 | 0.13201118 | 0.51087126 |
| Vitamin D \|\| id:ukb-d-30890_irnt | hcovid_vs_pop | Weighted median | 54 | -0.0729352 | 0.12079855 | 0.54599269 |
| Vitamin D \|\| id:ukb-d-30890_irnt | hcovid_vs_pop | Inverse variance weighted | 54 | -0.0253199 | 0.08552864 | 0.76719924 |
| Vitamin D \|\| id:ukb-d-30890_irnt | hcovid_vs_pop | Simple mode | 54 | 0.19607395 | 0.23499356 | 0.40781196 |
| Vitamin D \|\| id:ukb-d-30890_irnt | hcovid_vs_pop | Weighted mode | 54 | -0.0997946 | 0.1047708 | 0.34516535 |
| Waist circumference \|\| id:ukb-b-9405 | hcovid_vs_pop | MR Egger | 355 | 0.43347842 | 0.21662428 | 0.04615101 |
| Waist circumference \|\| id:ukb-b-9405 | hcovid_vs_pop | Weighted median | 355 | 0.4102271 | 0.1392834 | 0.00322674 |
| Waist circumference \|\| id:ukb-b-9405 | hcovid_vs_pop | Inverse variance weighted | 355 | 0.53142014 | 0.07689614 | 4.82E-12 |
| Waist circumference \|\| id:ukb-b-9405 | hcovid_vs_pop | Simple mode | 355 | 0.60056535 | 0.35709314 | 0.09348619 |
| Waist circumference \|\| id:ukb-b-9405 | hcovid_vs_pop | Weighted mode | 355 | 0.34918853 | 0.22103563 | 0.11504945 |
| Waist-to-hip ratio \|\| id:ieu-a-73 | hcovid_vs_pop | MR Egger | 29 | -0.3193257 | 0.62982023 | 0.61626326 |
| Waist-to-hip ratio \|\| id:ieu-a-73 | hcovid_vs_pop | Weighted median | 29 | 0.02123352 | 0.20615245 | 0.91796367 |
| Waist-to-hip ratio \|\| id:ieu-a-73 | hcovid_vs_pop | Inverse variance weighted | 29 | 0.14547673 | 0.14763916 | 0.32445056 |
| Waist-to-hip ratio \|\| id:ieu-a-73 | hcovid_vs_pop | Simple mode | 29 | -0.1663272 | 0.37488458 | 0.66068676 |
| Waist-to-hip ratio \|\| id:ieu-a-73 | hcovid_vs_pop | Weighted mode | 29 | -0.0385647 | 0.31622139 | 0.90380593 |
| Weight \|\| id:ukb-b-11842 | hcovid_vs_pop | MR Egger | 478 | 0.25287837 | 0.1566653 | 0.10716162 |
| Weight \|\| id:ukb-b-11842 | hcovid_vs_pop | Weighted median | 478 | 0.27473985 | 0.11008609 | 0.01257153 |
| Weight \|\| id:ukb-b-11842 | hcovid_vs_pop | Inverse variance weighted | 478 | 0.40724231 | 0.06142339 | 3.35E-11 |
| Weight \|\| id:ukb-b-11842 | hcovid_vs_pop | Simple mode | 478 | 0.10131463 | 0.32682575 | 0.7566995 |
| Weight \|\| id:ukb-b-11842 | hcovid_vs_pop | Weighted mode | 478 | 0.15951623 | 0.19864099 | 0.4223528 |
| Whole body fat mass \|\| id:ukb-b-19393 | hcovid_vs_pop | MR Egger | 412 | 0.42759109 | 0.17364907 | 0.01421195 |
| Whole body fat mass \|\| id:ukb-b-19393 | hcovid_vs_pop | Weighted median | 412 | 0.39579631 | 0.1099547 | 0.00031867 |
| Whole body fat mass \|\| id:ukb-b-19393 | hcovid_vs_pop | Inverse variance weighted | 412 | 0.44966697 | 0.06284392 | 8.35E-13 |
| Whole body fat mass \|\| id:ukb-b-19393 | hcovid_vs_pop | Simple mode | 412 | 0.49447843 | 0.32172353 | 0.12507087 |
| Whole body fat mass \|\| id:ukb-b-19393 | hcovid_vs_pop | Weighted mode | 412 | 0.28939664 | 0.2105351 | 0.1700117 |
| Whole body fat-free mass \|\| id:ukb-b-13354 | hcovid_vs_pop | MR Egger | 525 | 0.093561 | 0.18359187 | 0.61053682 |
| Whole body fat-free mass \|\| id:ukb-b-13354 | hcovid_vs_pop | Weighted median | 525 | 0.13326593 | 0.11936865 | 0.26424101 |
| Whole body fat-free mass \|\| id:ukb-b-13354 | hcovid_vs_pop | Inverse variance weighted | 525 | 0.26702018 | 0.07568106 | 0.00041835 |
| Whole body fat-free mass \|\| id:ukb-b-13354 | hcovid_vs_pop | Simple mode | 525 | 0.03018275 | 0.40930959 | 0.9412449 |
| Whole body fat-free mass \|\| id:ukb-b-13354 | hcovid_vs_pop | Weighted mode | 525 | -0.0658881 | 0.27679419 | 0.81194303 |
| bmi | hcovid_vs_pop | MR Egger | 513 | 0.38665114 | 0.17227783 | 0.02523731 |
| bmi | hcovid_vs_pop | Weighted median | 513 | 0.43789783 | 0.10727954 | 4.47E-05 |
| bmi | hcovid_vs_pop | Inverse variance weighted | 513 | 0.40579175 | 0.06478257 | 3.75E-10 |
| bmi | hcovid_vs_pop | Simple mode | 513 | 0.5079486 | 0.32897147 | 0.1231939 |
| bmi | hcovid_vs_pop | Weighted mode | 513 | 0.30872842 | 0.18132579 | 0.08924666 |
| height | hcovid_vs_pop | MR Egger | 812 | 0.15382755 | 0.08042415 | 0.05613842 |
| height | hcovid_vs_pop | Weighted median | 812 | 0.0220272 | 0.05962482 | 0.71180721 |
| height | hcovid_vs_pop | Inverse variance weighted | 812 | 0.03323862 | 0.03785571 | 0.37992498 |
| height | hcovid_vs_pop | Simple mode | 812 | -0.2238755 | 0.17028128 | 0.18896914 |
| height | hcovid_vs_pop | Weighted mode | 812 | -0.0188949 | 0.11152734 | 0.86550876 |
| interleukin 6 receptor \|\| id:prot-b-23 | hcovid_vs_pop | Inverse variance weighted | 2 | -0.0516388 | 0.0223987 | 0.02114214 |
| WHRadj | hcovid_vs_pop | MR Egger | 316 | 0.14883485 | 0.16906339 | 0.37934334 |
| WHRadj | hcovid_vs_pop | Weighted median | 316 | 0.09585537 | 0.10747054 | 0.37243461 |
| WHRadj | hcovid_vs_pop | Inverse variance weighted | 316 | 0.00716609 | 0.06938437 | 0.91773995 |
| WHRadj | hcovid_vs_pop | Simple mode | 316 | 0.20630602 | 0.28350712 | 0.46734234 |
| WHRadj | hcovid_vs_pop | Weighted mode | 316 | 0.05526022 | 0.16585095 | 0.73921097 |
| Adiponectin | scovid_vs_pop | MR Egger | 17 | -0.0356419 | 0.15704471 | 0.82352375 |
| Adiponectin | scovid_vs_pop | Weighted median | 17 | -0.0357994 | 0.10416232 | 0.73108035 |
| Adiponectin | scovid_vs_pop | Inverse variance weighted | 17 | 0.00696566 | 0.09934588 | 0.94410192 |
| Adiponectin | scovid_vs_pop | Simple mode | 17 | 0.05940284 | 0.24515767 | 0.81162264 |
| Adiponectin | scovid_vs_pop | Weighted mode | 17 | -0.0250632 | 0.10086915 | 0.80692998 |
| Alanine aminotransferase \|\| id:ukb-d-30620_irnt | scovid_vs_pop | MR Egger | 180 | -0.2296031 | 0.22945169 | 0.3183496 |
| Alanine aminotransferase \|\| id:ukb-d-30620_irnt | scovid_vs_pop | Weighted median | 180 | -0.0435873 | 0.1763967 | 0.80483237 |
| Alanine aminotransferase \|\| id:ukb-d-30620_irnt | scovid_vs_pop | Inverse variance weighted | 180 | 0.02652185 | 0.11228743 | 0.81328052 |
| Alanine aminotransferase \|\| id:ukb-d-30620_irnt | scovid_vs_pop | Simple mode | 180 | -0.4516629 | 0.47696578 | 0.34494069 |
| Alanine aminotransferase \|\| id:ukb-d-30620_irnt | scovid_vs_pop | Weighted mode | 180 | -0.3144496 | 0.34498008 | 0.36325745 |
| Albumin \|\| id:ukb-d-30600_irnt | scovid_vs_pop | MR Egger | 186 | -0.0612099 | 0.19094178 | 0.74890128 |
| Albumin \|\| id:ukb-d-30600_irnt | scovid_vs_pop | Weighted median | 186 | -0.0856579 | 0.1593257 | 0.59083411 |
| Albumin \|\| id:ukb-d-30600_irnt | scovid_vs_pop | Inverse variance weighted | 186 | -0.0589743 | 0.1006402 | 0.55788133 |
| Albumin \|\| id:ukb-d-30600_irnt | scovid_vs_pop | Simple mode | 186 | -0.3003466 | 0.35642637 | 0.4005062 |
| Albumin \|\| id:ukb-d-30600_irnt | scovid_vs_pop | Weighted mode | 186 | -0.1720241 | 0.21043831 | 0.41471926 |
| Alkaline phosphatase \|\| id:ukb-d-30610_irnt | scovid_vs_pop | MR Egger | 282 | 0.08343426 | 0.12764826 | 0.51388902 |
| Alkaline phosphatase \|\| id:ukb-d-30610_irnt | scovid_vs_pop | Weighted median | 282 | 0.02549845 | 0.1252431 | 0.8386726 |
| Alkaline phosphatase \|\| id:ukb-d-30610_irnt | scovid_vs_pop | Inverse variance weighted | 282 | 0.08250532 | 0.07705414 | 0.28428424 |
| Alkaline phosphatase \|\| id:ukb-d-30610_irnt | scovid_vs_pop | Simple mode | 282 | 0.04103632 | 0.28222488 | 0.88449701 |
| Alkaline phosphatase \|\| id:ukb-d-30610_irnt | scovid_vs_pop | Weighted mode | 282 | 0.07211631 | 0.14829194 | 0.62712426 |
| Apoliprotein A \|\| id:ukb-d-30630_irnt | scovid_vs_pop | MR Egger | 233 | 0.10973917 | 0.12915228 | 0.3963782 |
| Apoliprotein A \|\| id:ukb-d-30630_irnt | scovid_vs_pop | Weighted median | 233 | -0.0236539 | 0.12164822 | 0.84582709 |
| Apoliprotein A \|\| id:ukb-d-30630_irnt | scovid_vs_pop | Inverse variance weighted | 233 | 0.04428556 | 0.0782122 | 0.57124211 |
| Apoliprotein A \|\| id:ukb-d-30630_irnt | scovid_vs_pop | Simple mode | 233 | 0.10351014 | 0.24887291 | 0.67785618 |
| Apoliprotein A \|\| id:ukb-d-30630_irnt | scovid_vs_pop | Weighted mode | 233 | 0.01283844 | 0.12840664 | 0.92044442 |
| Apolipoprotein B \|\| id:ukb-d-30640_irnt | scovid_vs_pop | MR Egger | 148 | 0.08554815 | 0.1622374 | 0.59878405 |
| Apolipoprotein B \|\| id:ukb-d-30640_irnt | scovid_vs_pop | Weighted median | 148 | 0.0089037 | 0.14823487 | 0.95210402 |
| Apolipoprotein B \|\| id:ukb-d-30640_irnt | scovid_vs_pop | Inverse variance weighted | 148 | 0.0272611 | 0.09946757 | 0.78403066 |
| Apolipoprotein B \|\| id:ukb-d-30640_irnt | scovid_vs_pop | Simple mode | 148 | 0.10411781 | 0.29636103 | 0.72585069 |
| Apolipoprotein B \|\| id:ukb-d-30640_irnt | scovid_vs_pop | Weighted mode | 148 | 0.04260845 | 0.16144661 | 0.79221375 |
| Arm fat mass (left) \|\| id:ukb-b-8338 | scovid_vs_pop | MR Egger | 405 | 0.23558556 | 0.2549055 | 0.35593137 |
| Arm fat mass (left) \|\| id:ukb-b-8338 | scovid_vs_pop | Weighted median | 405 | 0.18505075 | 0.16031175 | 0.24836979 |
| Arm fat mass (left) \|\| id:ukb-b-8338 | scovid_vs_pop | Inverse variance weighted | 405 | 0.29908605 | 0.09516357 | 0.00167304 |
| Arm fat mass (left) \|\| id:ukb-b-8338 | scovid_vs_pop | Simple mode | 405 | -0.4326784 | 0.482811 | 0.37069819 |
| Arm fat mass (left) \|\| id:ukb-b-8338 | scovid_vs_pop | Weighted mode | 405 | -0.1689871 | 0.29149836 | 0.56242725 |
| Arm fat mass (right) \|\| id:ukb-b-6704 | scovid_vs_pop | MR Egger | 410 | 0.10535935 | 0.25520788 | 0.67994263 |
| Arm fat mass (right) \|\| id:ukb-b-6704 | scovid_vs_pop | Weighted median | 410 | 0.24278553 | 0.15817796 | 0.12481123 |
| Arm fat mass (right) \|\| id:ukb-b-6704 | scovid_vs_pop | Inverse variance weighted | 410 | 0.36740956 | 0.09696211 | 0.00015113 |
| Arm fat mass (right) \|\| id:ukb-b-6704 | scovid_vs_pop | Simple mode | 410 | -0.162565 | 0.4387056 | 0.71116006 |
| Arm fat mass (right) \|\| id:ukb-b-6704 | scovid_vs_pop | Weighted mode | 410 | -0.0884718 | 0.26869281 | 0.7421221 |
| Arm fat percentage (left) \|\| id:ukb-b-20188 | scovid_vs_pop | MR Egger | 380 | -0.0892196 | 0.38456835 | 0.81666418 |
| Arm fat percentage (left) \|\| id:ukb-b-20188 | scovid_vs_pop | Weighted median | 380 | 0.51478725 | 0.21145828 | 0.01491393 |
| Arm fat percentage (left) \|\| id:ukb-b-20188 | scovid_vs_pop | Inverse variance weighted | 380 | 0.54612959 | 0.13386821 | 4.51E-05 |
| Arm fat percentage (left) \|\| id:ukb-b-20188 | scovid_vs_pop | Simple mode | 380 | -0.7166093 | 0.69767023 | 0.30500675 |
| Arm fat percentage (left) \|\| id:ukb-b-20188 | scovid_vs_pop | Weighted mode | 380 | -0.5701537 | 0.44549206 | 0.20138809 |
| Arm fat percentage (right) \|\| id:ukb-b-12854 | scovid_vs_pop | MR Egger | 378 | 0.10897044 | 0.39951649 | 0.78519067 |
| Arm fat percentage (right) \|\| id:ukb-b-12854 | scovid_vs_pop | Weighted median | 378 | 0.43593144 | 0.21393606 | 0.04158278 |
| Arm fat percentage (right) \|\| id:ukb-b-12854 | scovid_vs_pop | Inverse variance weighted | 378 | 0.55998988 | 0.13982787 | 6.21E-05 |
| Arm fat percentage (right) \|\| id:ukb-b-12854 | scovid_vs_pop | Simple mode | 378 | -0.7848529 | 0.68869304 | 0.25516547 |
| Arm fat percentage (right) \|\| id:ukb-b-12854 | scovid_vs_pop | Weighted mode | 378 | -0.4294485 | 0.45575014 | 0.34664798 |
| Arm fat-free mass (left) \|\| id:ukb-b-19925 | scovid_vs_pop | MR Egger | 495 | -0.0981132 | 0.3313653 | 0.76728781 |
| Arm fat-free mass (left) \|\| id:ukb-b-19925 | scovid_vs_pop | Weighted median | 495 | 0.2212087 | 0.19683807 | 0.2610934 |
| Arm fat-free mass (left) \|\| id:ukb-b-19925 | scovid_vs_pop | Inverse variance weighted | 495 | 0.21841721 | 0.13099744 | 0.09544691 |
| Arm fat-free mass (left) \|\| id:ukb-b-19925 | scovid_vs_pop | Simple mode | 495 | 0.64737711 | 0.59571814 | 0.2776918 |
| Arm fat-free mass (left) \|\| id:ukb-b-19925 | scovid_vs_pop | Weighted mode | 495 | 0.07356979 | 0.36920286 | 0.84213622 |
| Arm fat-free mass (right) \|\| id:ukb-b-19520 | scovid_vs_pop | MR Egger | 497 | -0.2862009 | 0.33117572 | 0.38789857 |
| Arm fat-free mass (right) \|\| id:ukb-b-19520 | scovid_vs_pop | Weighted median | 497 | 0.22291786 | 0.20869401 | 0.28544993 |
| Arm fat-free mass (right) \|\| id:ukb-b-19520 | scovid_vs_pop | Inverse variance weighted | 497 | 0.26459869 | 0.13033794 | 0.04234666 |
| Arm fat-free mass (right) \|\| id:ukb-b-19520 | scovid_vs_pop | Simple mode | 497 | 0.48795947 | 0.58583699 | 0.40528676 |
| Arm fat-free mass (right) \|\| id:ukb-b-19520 | scovid_vs_pop | Weighted mode | 497 | 0.06015377 | 0.40878156 | 0.88307041 |
| Aspartate aminotransferase \|\| id:ukb-d-30650_irnt | scovid_vs_pop | MR Egger | 220 | -0.2111859 | 0.2355743 | 0.37098931 |
| Aspartate aminotransferase \|\| id:ukb-d-30650_irnt | scovid_vs_pop | Weighted median | 220 | -0.223681 | 0.16805062 | 0.18317798 |
| Aspartate aminotransferase \|\| id:ukb-d-30650_irnt | scovid_vs_pop | Inverse variance weighted | 220 | -0.2448526 | 0.11043325 | 0.02660943 |
| Aspartate aminotransferase \|\| id:ukb-d-30650_irnt | scovid_vs_pop | Simple mode | 220 | -0.1960878 | 0.39222815 | 0.61762424 |
| Aspartate aminotransferase \|\| id:ukb-d-30650_irnt | scovid_vs_pop | Weighted mode | 220 | -0.1635534 | 0.26188119 | 0.53292771 |
| Body fat percentage \|\| id:ukb-b-8909 | scovid_vs_pop | MR Egger | 375 | 0.38621738 | 0.43345445 | 0.37349183 |
| Body fat percentage \|\| id:ukb-b-8909 | scovid_vs_pop | Weighted median | 375 | 0.60617 | 0.20536275 | 0.00316026 |
| Body fat percentage \|\| id:ukb-b-8909 | scovid_vs_pop | Inverse variance weighted | 375 | 0.66703216 | 0.13916288 | 1.64E-06 |
| Body fat percentage \|\| id:ukb-b-8909 | scovid_vs_pop | Simple mode | 375 | 0.31121252 | 0.63332227 | 0.62343409 |
| Body fat percentage \|\| id:ukb-b-8909 | scovid_vs_pop | Weighted mode | 375 | 0.11478087 | 0.51196289 | 0.82272594 |
| C-reactive protein \|\| id:ukb-d-30710_irnt | scovid_vs_pop | MR Egger | 187 | 0.03467709 | 0.14445267 | 0.81055072 |
| C-reactive protein \|\| id:ukb-d-30710_irnt | scovid_vs_pop | Weighted median | 187 | 0.1428391 | 0.13603328 | 0.29370408 |
| C-reactive protein \|\| id:ukb-d-30710_irnt | scovid_vs_pop | Inverse variance weighted | 187 | 0.16804356 | 0.08521091 | 0.04859938 |
| C-reactive protein \|\| id:ukb-d-30710_irnt | scovid_vs_pop | Simple mode | 187 | -0.3390857 | 0.29927988 | 0.25867034 |
| C-reactive protein \|\| id:ukb-d-30710_irnt | scovid_vs_pop | Weighted mode | 187 | 0.08521982 | 0.12403553 | 0.49290081 |
| Calcium \|\| id:ukb-d-30680_irnt | scovid_vs_pop | MR Egger | 191 | -0.2884886 | 0.24560958 | 0.24163973 |
| Calcium \|\| id:ukb-d-30680_irnt | scovid_vs_pop | Weighted median | 191 | 0.03430554 | 0.15567495 | 0.82558576 |
| Calcium \|\| id:ukb-d-30680_irnt | scovid_vs_pop | Inverse variance weighted | 191 | 0.10273818 | 0.10917968 | 0.34670438 |
| Calcium \|\| id:ukb-d-30680_irnt | scovid_vs_pop | Simple mode | 191 | 0.10966898 | 0.40858398 | 0.78867314 |
| Calcium \|\| id:ukb-d-30680_irnt | scovid_vs_pop | Weighted mode | 191 | 0.07475204 | 0.35166221 | 0.83189202 |
| Cholesterol \|\| id:ukb-d-30690_irnt | scovid_vs_pop | MR Egger | 164 | 0.03932239 | 0.14698916 | 0.78940989 |
| Cholesterol \|\| id:ukb-d-30690_irnt | scovid_vs_pop | Weighted median | 164 | -0.0090802 | 0.13508546 | 0.94640824 |
| Cholesterol \|\| id:ukb-d-30690_irnt | scovid_vs_pop | Inverse variance weighted | 164 | 0.07204097 | 0.08844572 | 0.41534607 |
| Cholesterol \|\| id:ukb-d-30690_irnt | scovid_vs_pop | Simple mode | 164 | 0.20554325 | 0.26530561 | 0.43961486 |
| Cholesterol \|\| id:ukb-d-30690_irnt | scovid_vs_pop | Weighted mode | 164 | 0.00022889 | 0.14837011 | 0.99877097 |
| Creatinine \|\| id:ukb-d-30700_irnt | scovid_vs_pop | MR Egger | 307 | 0.23384021 | 0.23663686 | 0.32384695 |
| Creatinine \|\| id:ukb-d-30700_irnt | scovid_vs_pop | Weighted median | 307 | 0.2970061 | 0.15684104 | 0.05826804 |
| Creatinine \|\| id:ukb-d-30700_irnt | scovid_vs_pop | Inverse variance weighted | 307 | 0.19157562 | 0.10110674 | 0.05812077 |
| Creatinine \|\| id:ukb-d-30700_irnt | scovid_vs_pop | Simple mode | 307 | 0.61190165 | 0.42511586 | 0.15106612 |
| Creatinine \|\| id:ukb-d-30700_irnt | scovid_vs_pop | Weighted mode | 307 | 0.49848287 | 0.26174539 | 0.05778828 |
| Cystatin C \|\| id:ukb-d-30720_irnt | scovid_vs_pop | MR Egger | 289 | -0.0485165 | 0.17787522 | 0.78523727 |
| Cystatin C \|\| id:ukb-d-30720_irnt | scovid_vs_pop | Weighted median | 289 | 0.18089525 | 0.14776628 | 0.22087738 |
| Cystatin C \|\| id:ukb-d-30720_irnt | scovid_vs_pop | Inverse variance weighted | 289 | 0.04532791 | 0.08836803 | 0.60799004 |
| Cystatin C \|\| id:ukb-d-30720_irnt | scovid_vs_pop | Simple mode | 289 | -0.2027933 | 0.35997133 | 0.5736284 |
| Cystatin C \|\| id:ukb-d-30720_irnt | scovid_vs_pop | Weighted mode | 289 | 0.19161029 | 0.208081 | 0.3579022 |
| Diastolic blood pressure, automated reading \|\| id:ukb-b-7992 | scovid_vs_pop | MR Egger | 246 | -0.568654 | 0.42969079 | 0.18694017 |
| Diastolic blood pressure, automated reading \|\| id:ukb-b-7992 | scovid_vs_pop | Weighted median | 246 | -0.3031757 | 0.19070663 | 0.11189138 |
| Diastolic blood pressure, automated reading \|\| id:ukb-b-7992 | scovid_vs_pop | Inverse variance weighted | 246 | -0.2105947 | 0.1399376 | 0.13234495 |
| Diastolic blood pressure, automated reading \|\| id:ukb-b-7992 | scovid_vs_pop | Simple mode | 246 | 0.7259635 | 0.61980261 | 0.2426234 |
| Diastolic blood pressure, automated reading \|\| id:ukb-b-7992 | scovid_vs_pop | Weighted mode | 246 | 0.34683586 | 0.56306289 | 0.53847913 |
| Direct bilirubin \|\| id:ukb-d-30660_irnt | scovid_vs_pop | MR Egger | 78 | 0.07398572 | 0.14501495 | 0.61139432 |
| Direct bilirubin \|\| id:ukb-d-30660_irnt | scovid_vs_pop | Weighted median | 78 | -0.0280192 | 0.15361863 | 0.85527317 |
| Direct bilirubin \|\| id:ukb-d-30660_irnt | scovid_vs_pop | Inverse variance weighted | 78 | 0.01065105 | 0.10806064 | 0.92148331 |
| Direct bilirubin \|\| id:ukb-d-30660_irnt | scovid_vs_pop | Simple mode | 78 | 0.08428593 | 0.27274188 | 0.75813194 |
| Direct bilirubin \|\| id:ukb-d-30660_irnt | scovid_vs_pop | Weighted mode | 78 | 0.03215444 | 0.13876916 | 0.81737664 |
| Gamma glutamyltransferase \|\| id:ukb-d-30730_irnt | scovid_vs_pop | MR Egger | 257 | -0.0388588 | 0.14432876 | 0.78796404 |
| Gamma glutamyltransferase \|\| id:ukb-d-30730_irnt | scovid_vs_pop | Weighted median | 257 | -0.0546735 | 0.12663905 | 0.66593974 |
| Gamma glutamyltransferase \|\| id:ukb-d-30730_irnt | scovid_vs_pop | Inverse variance weighted | 257 | 0.0133545 | 0.08137927 | 0.86965087 |
| Gamma glutamyltransferase \|\| id:ukb-d-30730_irnt | scovid_vs_pop | Simple mode | 257 | -0.3738242 | 0.29022231 | 0.19888753 |
| Gamma glutamyltransferase \|\| id:ukb-d-30730_irnt | scovid_vs_pop | Weighted mode | 257 | -0.0848301 | 0.14540149 | 0.56012452 |
| Glucose \|\| id:ukb-d-30740_irnt | scovid_vs_pop | MR Egger | 102 | 0.13586209 | 0.23776114 | 0.56899532 |
| Glucose \|\| id:ukb-d-30740_irnt | scovid_vs_pop | Weighted median | 102 | 0.12141075 | 0.18297204 | 0.50697957 |
| Glucose \|\| id:ukb-d-30740_irnt | scovid_vs_pop | Inverse variance weighted | 102 | 0.22790064 | 0.12626551 | 0.07108535 |
| Glucose \|\| id:ukb-d-30740_irnt | scovid_vs_pop | Simple mode | 102 | 0.24629797 | 0.34212782 | 0.47324895 |
| Glucose \|\| id:ukb-d-30740_irnt | scovid_vs_pop | Weighted mode | 102 | 0.13900858 | 0.1764992 | 0.4327824 |
| Glycated haemoglobin \|\| id:ukb-d-30750_irnt | scovid_vs_pop | MR Egger | 276 | 0.1061004 | 0.15188619 | 0.48542454 |
| Glycated haemoglobin \|\| id:ukb-d-30750_irnt | scovid_vs_pop | Weighted median | 276 | 0.06005083 | 0.11353629 | 0.59686574 |
| Glycated haemoglobin \|\| id:ukb-d-30750_irnt | scovid_vs_pop | Inverse variance weighted | 276 | 0.05245507 | 0.07659339 | 0.49343787 |
| Glycated haemoglobin \|\| id:ukb-d-30750_irnt | scovid_vs_pop | Simple mode | 276 | 0.06179932 | 0.25028387 | 0.80515675 |
| Glycated haemoglobin \|\| id:ukb-d-30750_irnt | scovid_vs_pop | Weighted mode | 276 | 0.02716655 | 0.13973662 | 0.84599641 |
| HDL cholesterol \|\| id:ukb-d-30760_irnt | scovid_vs_pop | MR Egger | 252 | -0.0363612 | 0.11807185 | 0.75837082 |
| HDL cholesterol \|\| id:ukb-d-30760_irnt | scovid_vs_pop | Weighted median | 252 | 0.03044199 | 0.11068505 | 0.7832912 |
| HDL cholesterol \|\| id:ukb-d-30760_irnt | scovid_vs_pop | Inverse variance weighted | 252 | -0.0178217 | 0.07104395 | 0.80192602 |
| HDL cholesterol \|\| id:ukb-d-30760_irnt | scovid_vs_pop | Simple mode | 252 | -0.1568938 | 0.23924305 | 0.5125584 |
| HDL cholesterol \|\| id:ukb-d-30760_irnt | scovid_vs_pop | Weighted mode | 252 | 0.03182098 | 0.12108343 | 0.79291868 |
| Hip circumference \|\| id:ukb-b-15590 | scovid_vs_pop | MR Egger | 400 | 0.10240858 | 0.26314173 | 0.6973537 |
| Hip circumference \|\| id:ukb-b-15590 | scovid_vs_pop | Weighted median | 400 | 0.2521494 | 0.14841744 | 0.0893342 |
| Hip circumference \|\| id:ukb-b-15590 | scovid_vs_pop | Inverse variance weighted | 400 | 0.33549264 | 0.09745228 | 0.00057608 |
| Hip circumference \|\| id:ukb-b-15590 | scovid_vs_pop | Simple mode | 400 | 0.1060873 | 0.46259192 | 0.8187281 |
| Hip circumference \|\| id:ukb-b-15590 | scovid_vs_pop | Weighted mode | 400 | 0.1060873 | 0.30089708 | 0.72459694 |
| IGF-1 \|\| id:ukb-d-30770_irnt | scovid_vs_pop | MR Egger | 323 | -0.0527493 | 0.1656766 | 0.75039827 |
| IGF-1 \|\| id:ukb-d-30770_irnt | scovid_vs_pop | Weighted median | 323 | -0.0570357 | 0.12209699 | 0.64040345 |
| IGF-1 \|\| id:ukb-d-30770_irnt | scovid_vs_pop | Inverse variance weighted | 323 | -0.0381434 | 0.08230062 | 0.64303163 |
| IGF-1 \|\| id:ukb-d-30770_irnt | scovid_vs_pop | Simple mode | 323 | 0.28873398 | 0.29649608 | 0.33087662 |
| IGF-1 \|\| id:ukb-d-30770_irnt | scovid_vs_pop | Weighted mode | 323 | 0.00382204 | 0.16618759 | 0.98166584 |
| Insulin Resistance | scovid_vs_pop | MR Egger | 49 | -0.2416625 | 0.40672875 | 0.5552535 |
| Insulin Resistance | scovid_vs_pop | Weighted median | 49 | -0.1876219 | 0.3287665 | 0.5682137 |
| Insulin Resistance | scovid_vs_pop | Inverse variance weighted | 49 | -0.0490691 | 0.21682959 | 0.82096594 |
| Insulin Resistance | scovid_vs_pop | Simple mode | 49 | 0.02567376 | 0.65112395 | 0.96871118 |
| Insulin Resistance | scovid_vs_pop | Weighted mode | 49 | -0.1403093 | 0.3304591 | 0.67303345 |
| LDL direct \|\| id:ukb-d-30780_irnt | scovid_vs_pop | MR Egger | 146 | -0.1068899 | 0.15133651 | 0.48113885 |
| LDL direct \|\| id:ukb-d-30780_irnt | scovid_vs_pop | Weighted median | 146 | -0.0537715 | 0.1412366 | 0.70341174 |
| LDL direct \|\| id:ukb-d-30780_irnt | scovid_vs_pop | Inverse variance weighted | 146 | -0.0377282 | 0.09680087 | 0.69672059 |
| LDL direct \|\| id:ukb-d-30780_irnt | scovid_vs_pop | Simple mode | 146 | 0.00164136 | 0.26546766 | 0.99507529 |
| LDL direct \|\| id:ukb-d-30780_irnt | scovid_vs_pop | Weighted mode | 146 | -0.0293638 | 0.14589811 | 0.84077559 |
| Leg fat mass (left) \|\| id:ukb-b-7212 | scovid_vs_pop | MR Egger | 403 | 0.34413619 | 0.3187521 | 0.28095331 |
| Leg fat mass (left) \|\| id:ukb-b-7212 | scovid_vs_pop | Weighted median | 403 | 0.32025534 | 0.1970338 | 0.10408103 |
| Leg fat mass (left) \|\| id:ukb-b-7212 | scovid_vs_pop | Inverse variance weighted | 403 | 0.48877052 | 0.11666898 | 2.80E-05 |
| Leg fat mass (left) \|\| id:ukb-b-7212 | scovid_vs_pop | Simple mode | 403 | 0.22391964 | 0.59800686 | 0.70827199 |
| Leg fat mass (left) \|\| id:ukb-b-7212 | scovid_vs_pop | Weighted mode | 403 | 0.0177478 | 0.33975983 | 0.95836634 |
| Leg fat mass (right) \|\| id:ukb-b-18096 | scovid_vs_pop | MR Egger | 408 | 0.20746149 | 0.31951775 | 0.51651442 |
| Leg fat mass (right) \|\| id:ukb-b-18096 | scovid_vs_pop | Weighted median | 408 | 0.51334949 | 0.19309413 | 0.00784788 |
| Leg fat mass (right) \|\| id:ukb-b-18096 | scovid_vs_pop | Inverse variance weighted | 408 | 0.60434256 | 0.11625579 | 2.01E-07 |
| Leg fat mass (right) \|\| id:ukb-b-18096 | scovid_vs_pop | Simple mode | 408 | 0.41896781 | 0.62810047 | 0.50512491 |
| Leg fat mass (right) \|\| id:ukb-b-18096 | scovid_vs_pop | Weighted mode | 408 | 0.12848885 | 0.37916405 | 0.7348794 |
| Leg fat percentage (left) \|\| id:ukb-b-18377 | scovid_vs_pop | MR Egger | 360 | 0.38221283 | 0.54143996 | 0.48069753 |
| Leg fat percentage (left) \|\| id:ukb-b-18377 | scovid_vs_pop | Weighted median | 360 | 0.74360805 | 0.25842203 | 0.00400847 |
| Leg fat percentage (left) \|\| id:ukb-b-18377 | scovid_vs_pop | Inverse variance weighted | 360 | 0.89022501 | 0.16901885 | 1.39E-07 |
| Leg fat percentage (left) \|\| id:ukb-b-18377 | scovid_vs_pop | Simple mode | 360 | 0.96336334 | 0.8812431 | 0.27504478 |
| Leg fat percentage (left) \|\| id:ukb-b-18377 | scovid_vs_pop | Weighted mode | 360 | 0.65411939 | 0.59480203 | 0.27218833 |
| Leg fat percentage (right) \|\| id:ukb-b-20531 | scovid_vs_pop | MR Egger | 366 | 0.180247 | 0.5404456 | 0.73893721 |
| Leg fat percentage (right) \|\| id:ukb-b-20531 | scovid_vs_pop | Weighted median | 366 | 0.7967837 | 0.25308142 | 0.00164207 |
| Leg fat percentage (right) \|\| id:ukb-b-20531 | scovid_vs_pop | Inverse variance weighted | 366 | 0.98023043 | 0.17183514 | 1.17E-08 |
| Leg fat percentage (right) \|\| id:ukb-b-20531 | scovid_vs_pop | Simple mode | 366 | 1.21167791 | 0.89375127 | 0.17602597 |
| Leg fat percentage (right) \|\| id:ukb-b-20531 | scovid_vs_pop | Weighted mode | 366 | 0.91067751 | 0.63308019 | 0.15115286 |
| Leg fat-free mass (left) \|\| id:ukb-b-16099 | scovid_vs_pop | MR Egger | 486 | -0.1109594 | 0.31029454 | 0.72080325 |
| Leg fat-free mass (left) \|\| id:ukb-b-16099 | scovid_vs_pop | Weighted median | 486 | 0.007711 | 0.19777116 | 0.96889875 |
| Leg fat-free mass (left) \|\| id:ukb-b-16099 | scovid_vs_pop | Inverse variance weighted | 486 | 0.09679473 | 0.12749458 | 0.44772895 |
| Leg fat-free mass (left) \|\| id:ukb-b-16099 | scovid_vs_pop | Simple mode | 486 | 0.02208476 | 0.57249264 | 0.96924392 |
| Leg fat-free mass (left) \|\| id:ukb-b-16099 | scovid_vs_pop | Weighted mode | 486 | -0.0821693 | 0.33453445 | 0.80607817 |
| Leg fat-free mass (right) \|\| id:ukb-b-12828 | scovid_vs_pop | MR Egger | 491 | -0.2001904 | 0.29830328 | 0.50247564 |
| Leg fat-free mass (right) \|\| id:ukb-b-12828 | scovid_vs_pop | Weighted median | 491 | 0.07795838 | 0.18816609 | 0.67864987 |
| Leg fat-free mass (right) \|\| id:ukb-b-12828 | scovid_vs_pop | Inverse variance weighted | 491 | 0.01514081 | 0.12195459 | 0.90119558 |
| Leg fat-free mass (right) \|\| id:ukb-b-12828 | scovid_vs_pop | Simple mode | 491 | 0.01976344 | 0.5207017 | 0.9697387 |
| Leg fat-free mass (right) \|\| id:ukb-b-12828 | scovid_vs_pop | Weighted mode | 491 | -0.032273 | 0.33330638 | 0.92290347 |
| Lipoprotein A \|\| id:ukb-d-30790_irnt | scovid_vs_pop | MR Egger | 13 | 0.4074746 | 0.25490059 | 0.13822336 |
| Lipoprotein A \|\| id:ukb-d-30790_irnt | scovid_vs_pop | Weighted median | 13 | -0.0365615 | 0.19151843 | 0.84860136 |
| Lipoprotein A \|\| id:ukb-d-30790_irnt | scovid_vs_pop | Inverse variance weighted | 13 | -0.1267261 | 0.18926006 | 0.503121 |
| Lipoprotein A \|\| id:ukb-d-30790_irnt | scovid_vs_pop | Simple mode | 13 | 0.20223129 | 0.23548789 | 0.40729163 |
| Lipoprotein A \|\| id:ukb-d-30790_irnt | scovid_vs_pop | Weighted mode | 13 | 0.00941151 | 0.19037174 | 0.96138395 |
| Oestradiol \|\| id:ukb-d-30800_irnt | scovid_vs_pop | Wald ratio | 1 | -0.5488565 | 0.86136451 | 0.52399835 |
| Phosphate \|\| id:ukb-d-30810_irnt | scovid_vs_pop | MR Egger | 136 | 0.1050379 | 0.23573193 | 0.65661828 |
| Phosphate \|\| id:ukb-d-30810_irnt | scovid_vs_pop | Weighted median | 136 | -0.0010733 | 0.17798743 | 0.99518871 |
| Phosphate \|\| id:ukb-d-30810_irnt | scovid_vs_pop | Inverse variance weighted | 136 | 0.05179823 | 0.12533436 | 0.67940121 |
| Phosphate \|\| id:ukb-d-30810_irnt | scovid_vs_pop | Simple mode | 136 | -0.4300259 | 0.46022705 | 0.35177735 |
| Phosphate \|\| id:ukb-d-30810_irnt | scovid_vs_pop | Weighted mode | 136 | 0.02111469 | 0.20127192 | 0.91660589 |
| SHBG \|\| id:ukb-d-30830_irnt | scovid_vs_pop | MR Egger | 236 | 0.13320035 | 0.14821966 | 0.36975303 |
| SHBG \|\| id:ukb-d-30830_irnt | scovid_vs_pop | Weighted median | 236 | 0.13556583 | 0.13349167 | 0.30984957 |
| SHBG \|\| id:ukb-d-30830_irnt | scovid_vs_pop | Inverse variance weighted | 236 | 0.08274696 | 0.08286096 | 0.31797675 |
| SHBG \|\| id:ukb-d-30830_irnt | scovid_vs_pop | Simple mode | 236 | 0.14326767 | 0.31497406 | 0.64963314 |
| SHBG \|\| id:ukb-d-30830_irnt | scovid_vs_pop | Weighted mode | 236 | 0.06094378 | 0.16315007 | 0.70908013 |
| Systolic blood pressure, automated reading \|\| id:ukb-b-20175 | scovid_vs_pop | MR Egger | 232 | 0.60747073 | 0.42066992 | 0.15008414 |
| Systolic blood pressure, automated reading \|\| id:ukb-b-20175 | scovid_vs_pop | Weighted median | 232 | -0.0501892 | 0.19549951 | 0.79739286 |
| Systolic blood pressure, automated reading \|\| id:ukb-b-20175 | scovid_vs_pop | Inverse variance weighted | 232 | -0.0935699 | 0.13797991 | 0.49768208 |
| Systolic blood pressure, automated reading \|\| id:ukb-b-20175 | scovid_vs_pop | Simple mode | 232 | -0.9693618 | 0.62871648 | 0.12448837 |
| Systolic blood pressure, automated reading \|\| id:ukb-b-20175 | scovid_vs_pop | Weighted mode | 232 | 0.5517725 | 0.40883705 | 0.17846074 |
| Testosterone \|\| id:ukb-d-30850_irnt | scovid_vs_pop | MR Egger | 90 | -1.0144849 | 0.45212874 | 0.02735504 |
| Testosterone \|\| id:ukb-d-30850_irnt | scovid_vs_pop | Weighted median | 90 | -0.6964609 | 0.37113463 | 0.06057674 |
| Testosterone \|\| id:ukb-d-30850_irnt | scovid_vs_pop | Inverse variance weighted | 90 | -0.3945063 | 0.25890529 | 0.12757175 |
| Testosterone \|\| id:ukb-d-30850_irnt | scovid_vs_pop | Simple mode | 90 | -0.2660062 | 0.90497248 | 0.76948934 |
| Testosterone \|\| id:ukb-d-30850_irnt | scovid_vs_pop | Weighted mode | 90 | -0.572186 | 0.34376155 | 0.0995325 |
| Total bilirubin \|\| id:ukb-d-30840_irnt | scovid_vs_pop | MR Egger | 120 | 0.48664128 | 0.15855739 | 0.00266357 |
| Total bilirubin \|\| id:ukb-d-30840_irnt | scovid_vs_pop | Weighted median | 120 | 0.47178546 | 0.1556406 | 0.00243544 |
| Total bilirubin \|\| id:ukb-d-30840_irnt | scovid_vs_pop | Inverse variance weighted | 120 | 0.10762351 | 0.11509941 | 0.34976341 |
| Total bilirubin \|\| id:ukb-d-30840_irnt | scovid_vs_pop | Simple mode | 120 | 0.01625194 | 0.30978584 | 0.95824867 |
| Total bilirubin \|\| id:ukb-d-30840_irnt | scovid_vs_pop | Weighted mode | 120 | 0.42558539 | 0.14729275 | 0.00458803 |
| Total protein \|\| id:ukb-d-30860_irnt | scovid_vs_pop | MR Egger | 214 | 0.02263142 | 0.21040048 | 0.91444365 |
| Total protein \|\| id:ukb-d-30860_irnt | scovid_vs_pop | Weighted median | 214 | 0.11352321 | 0.160623 | 0.47971062 |
| Total protein \|\| id:ukb-d-30860_irnt | scovid_vs_pop | Inverse variance weighted | 214 | 0.14627594 | 0.09683677 | 0.13090537 |
| Total protein \|\| id:ukb-d-30860_irnt | scovid_vs_pop | Simple mode | 214 | -0.0833024 | 0.36525011 | 0.81981092 |
| Total protein \|\| id:ukb-d-30860_irnt | scovid_vs_pop | Weighted mode | 214 | 0.03848023 | 0.20971646 | 0.85459051 |
| Triglycerides \|\| id:ukb-d-30870_irnt | scovid_vs_pop | MR Egger | 213 | 0.26953571 | 0.12285322 | 0.0293297 |
| Triglycerides \|\| id:ukb-d-30870_irnt | scovid_vs_pop | Weighted median | 213 | 0.1796575 | 0.11763447 | 0.12669832 |
| Triglycerides \|\| id:ukb-d-30870_irnt | scovid_vs_pop | Inverse variance weighted | 213 | 0.23813016 | 0.07580988 | 0.00168286 |
| Triglycerides \|\| id:ukb-d-30870_irnt | scovid_vs_pop | Simple mode | 213 | -0.0473163 | 0.23469754 | 0.84041848 |
| Triglycerides \|\| id:ukb-d-30870_irnt | scovid_vs_pop | Weighted mode | 213 | 0.15769928 | 0.10842942 | 0.14731557 |
| Trunk fat mass \|\| id:ukb-b-20044 | scovid_vs_pop | MR Egger | 403 | 0.34192145 | 0.2659842 | 0.19936113 |
| Trunk fat mass \|\| id:ukb-b-20044 | scovid_vs_pop | Weighted median | 403 | 0.38267774 | 0.15193114 | 0.01177696 |
| Trunk fat mass \|\| id:ukb-b-20044 | scovid_vs_pop | Inverse variance weighted | 403 | 0.40421758 | 0.09592292 | 2.51E-05 |
| Trunk fat mass \|\| id:ukb-b-20044 | scovid_vs_pop | Simple mode | 403 | 0.55959374 | 0.47279289 | 0.23727415 |
| Trunk fat mass \|\| id:ukb-b-20044 | scovid_vs_pop | Weighted mode | 403 | 0.3009711 | 0.31468524 | 0.33943514 |
| Trunk fat percentage \|\| id:ukb-b-16407 | scovid_vs_pop | MR Egger | 371 | 0.6894462 | 0.36879432 | 0.06235067 |
| Trunk fat percentage \|\| id:ukb-b-16407 | scovid_vs_pop | Weighted median | 371 | 0.52485574 | 0.17053176 | 0.00208563 |
| Trunk fat percentage \|\| id:ukb-b-16407 | scovid_vs_pop | Inverse variance weighted | 371 | 0.56640489 | 0.11849663 | 1.75E-06 |
| Trunk fat percentage \|\| id:ukb-b-16407 | scovid_vs_pop | Simple mode | 371 | 0.59062742 | 0.55104645 | 0.28449558 |
| Trunk fat percentage \|\| id:ukb-b-16407 | scovid_vs_pop | Weighted mode | 371 | 0.47713237 | 0.4307681 | 0.26874046 |
| Trunk fat-free mass \|\| id:ukb-b-17409 | scovid_vs_pop | MR Egger | 538 | -0.1556393 | 0.27484191 | 0.57143603 |
| Trunk fat-free mass \|\| id:ukb-b-17409 | scovid_vs_pop | Weighted median | 538 | 0.21627144 | 0.17459315 | 0.21545047 |
| Trunk fat-free mass \|\| id:ukb-b-17409 | scovid_vs_pop | Inverse variance weighted | 538 | 0.12699996 | 0.11462359 | 0.26787304 |
| Trunk fat-free mass \|\| id:ukb-b-17409 | scovid_vs_pop | Simple mode | 538 | 0.56005807 | 0.54962738 | 0.30867238 |
| Trunk fat-free mass \|\| id:ukb-b-17409 | scovid_vs_pop | Weighted mode | 538 | 0.2126011 | 0.35014881 | 0.54399238 |
| Type 2 diabetes \|\| id:ebi-a-GCST006867 | scovid_vs_pop | MR Egger | 115 | -0.0041536 | 0.0939587 | 0.9648174 |
| Type 2 diabetes \|\| id:ebi-a-GCST006867 | scovid_vs_pop | Weighted median | 115 | 0.01447624 | 0.07521469 | 0.84737749 |
| Type 2 diabetes \|\| id:ebi-a-GCST006867 | scovid_vs_pop | Inverse variance weighted | 115 | -0.0201721 | 0.04102306 | 0.6229136 |
| Type 2 diabetes \|\| id:ebi-a-GCST006867 | scovid_vs_pop | Simple mode | 115 | -0.0418001 | 0.15010998 | 0.78116119 |
| Type 2 diabetes \|\| id:ebi-a-GCST006867 | scovid_vs_pop | Weighted mode | 115 | -0.0075838 | 0.08271488 | 0.92710838 |
| Urate \|\| id:ukb-d-30880_irnt | scovid_vs_pop | MR Egger | 226 | -0.0044695 | 0.17211935 | 0.97930654 |
| Urate \|\| id:ukb-d-30880_irnt | scovid_vs_pop | Weighted median | 226 | -0.0816906 | 0.1517118 | 0.59025998 |
| Urate \|\| id:ukb-d-30880_irnt | scovid_vs_pop | Inverse variance weighted | 226 | 0.02591718 | 0.10332534 | 0.8019449 |
| Urate \|\| id:ukb-d-30880_irnt | scovid_vs_pop | Simple mode | 226 | 0.11849174 | 0.34695557 | 0.73303204 |
| Urate \|\| id:ukb-d-30880_irnt | scovid_vs_pop | Weighted mode | 226 | -0.1306417 | 0.17461394 | 0.45513627 |
| Urea \|\| id:ukb-d-30670_irnt | scovid_vs_pop | MR Egger | 153 | 0.41245883 | 0.28644985 | 0.15196601 |
| Urea \|\| id:ukb-d-30670_irnt | scovid_vs_pop | Weighted median | 153 | 0.10142864 | 0.17936794 | 0.57174865 |
| Urea \|\| id:ukb-d-30670_irnt | scovid_vs_pop | Inverse variance weighted | 153 | 0.03840636 | 0.12530008 | 0.75921254 |
| Urea \|\| id:ukb-d-30670_irnt | scovid_vs_pop | Simple mode | 153 | -0.0226533 | 0.43459882 | 0.95849774 |
| Urea \|\| id:ukb-d-30670_irnt | scovid_vs_pop | Weighted mode | 153 | 0.31515556 | 0.285885 | 0.27203639 |
| Vitamin D \|\| id:ukb-d-30890_irnt | scovid_vs_pop | MR Egger | 56 | -0.0817259 | 0.18771402 | 0.66502593 |
| Vitamin D \|\| id:ukb-d-30890_irnt | scovid_vs_pop | Weighted median | 56 | -0.0945232 | 0.17510587 | 0.58933067 |
| Vitamin D \|\| id:ukb-d-30890_irnt | scovid_vs_pop | Inverse variance weighted | 56 | 0.07236748 | 0.12221761 | 0.55377029 |
| Vitamin D \|\| id:ukb-d-30890_irnt | scovid_vs_pop | Simple mode | 56 | 0.00921989 | 0.34957355 | 0.97905396 |
| Vitamin D \|\| id:ukb-d-30890_irnt | scovid_vs_pop | Weighted mode | 56 | -0.1142593 | 0.16711424 | 0.49702256 |
| Waist circumference \|\| id:ukb-b-9405 | scovid_vs_pop | MR Egger | 355 | 0.28869833 | 0.34196847 | 0.39911545 |
| Waist circumference \|\| id:ukb-b-9405 | scovid_vs_pop | Weighted median | 355 | 0.32603549 | 0.19125353 | 0.08824492 |
| Waist circumference \|\| id:ukb-b-9405 | scovid_vs_pop | Inverse variance weighted | 355 | 0.40638716 | 0.12256491 | 0.00091417 |
| Waist circumference \|\| id:ukb-b-9405 | scovid_vs_pop | Simple mode | 355 | 0.20568761 | 0.56851028 | 0.71771658 |
| Waist circumference \|\| id:ukb-b-9405 | scovid_vs_pop | Weighted mode | 355 | 0.01277252 | 0.32709236 | 0.9688736 |
| Waist-to-hip ratio \|\| id:ieu-a-73 | scovid_vs_pop | MR Egger | 29 | -0.7879274 | 0.92798228 | 0.40330473 |
| Waist-to-hip ratio \|\| id:ieu-a-73 | scovid_vs_pop | Weighted median | 29 | -0.112175 | 0.3040416 | 0.71216777 |
| Waist-to-hip ratio \|\| id:ieu-a-73 | scovid_vs_pop | Inverse variance weighted | 29 | -0.047388 | 0.21963008 | 0.82917282 |
| Waist-to-hip ratio \|\| id:ieu-a-73 | scovid_vs_pop | Simple mode | 29 | 0.10301796 | 0.53351434 | 0.84827986 |
| Waist-to-hip ratio \|\| id:ieu-a-73 | scovid_vs_pop | Weighted mode | 29 | -0.0662726 | 0.42415574 | 0.87696021 |
| Weight \|\| id:ukb-b-11842 | scovid_vs_pop | MR Egger | 479 | 0.29122385 | 0.24349053 | 0.23227604 |
| Weight \|\| id:ukb-b-11842 | scovid_vs_pop | Weighted median | 479 | 0.20849008 | 0.16011946 | 0.19288535 |
| Weight \|\| id:ukb-b-11842 | scovid_vs_pop | Inverse variance weighted | 479 | 0.26724798 | 0.09630825 | 0.00552148 |
| Weight \|\| id:ukb-b-11842 | scovid_vs_pop | Simple mode | 479 | -0.2063222 | 0.49819341 | 0.67895684 |
| Weight \|\| id:ukb-b-11842 | scovid_vs_pop | Weighted mode | 479 | -0.0423309 | 0.28524723 | 0.88208902 |
| Whole body fat mass \|\| id:ukb-b-19393 | scovid_vs_pop | MR Egger | 412 | 0.27569188 | 0.26282699 | 0.29481992 |
| Whole body fat mass \|\| id:ukb-b-19393 | scovid_vs_pop | Weighted median | 412 | 0.29687326 | 0.15331061 | 0.05281666 |
| Whole body fat mass \|\| id:ukb-b-19393 | scovid_vs_pop | Inverse variance weighted | 412 | 0.42801432 | 0.09612078 | 8.47E-06 |
| Whole body fat mass \|\| id:ukb-b-19393 | scovid_vs_pop | Simple mode | 412 | -0.3895229 | 0.51682783 | 0.45147285 |
| Whole body fat mass \|\| id:ukb-b-19393 | scovid_vs_pop | Weighted mode | 412 | -0.131269 | 0.31426095 | 0.67637951 |
| Whole body fat-free mass \|\| id:ukb-b-13354 | scovid_vs_pop | MR Egger | 527 | -0.1032996 | 0.2895991 | 0.72146064 |
| Whole body fat-free mass \|\| id:ukb-b-13354 | scovid_vs_pop | Weighted median | 527 | 0.11724203 | 0.18299705 | 0.52173245 |
| Whole body fat-free mass \|\| id:ukb-b-13354 | scovid_vs_pop | Inverse variance weighted | 527 | 0.2241088 | 0.11989244 | 0.06158821 |
| Whole body fat-free mass \|\| id:ukb-b-13354 | scovid_vs_pop | Simple mode | 527 | 0.0275428 | 0.52699013 | 0.95833789 |
| Whole body fat-free mass \|\| id:ukb-b-13354 | scovid_vs_pop | Weighted mode | 527 | 0.0275428 | 0.34546439 | 0.93648479 |
| bmi | scovid_vs_pop | MR Egger | 513 | 0.46761611 | 0.25418905 | 0.06640112 |
| bmi | scovid_vs_pop | Weighted median | 513 | 0.29076683 | 0.15515233 | 0.06092034 |
| bmi | scovid_vs_pop | Inverse variance weighted | 513 | 0.36295322 | 0.09570762 | 0.00014925 |
| bmi | scovid_vs_pop | Simple mode | 513 | -0.3017571 | 0.50742225 | 0.55231579 |
| bmi | scovid_vs_pop | Weighted mode | 513 | 0.11233951 | 0.2865753 | 0.69521619 |
| height | scovid_vs_pop | MR Egger | 813 | 0.04868582 | 0.12051186 | 0.68632517 |
| height | scovid_vs_pop | Weighted median | 813 | 0.00651975 | 0.08943232 | 0.94188451 |
| height | scovid_vs_pop | Inverse variance weighted | 813 | 0.03531618 | 0.05678631 | 0.53399864 |
| height | scovid_vs_pop | Simple mode | 813 | 0.01487567 | 0.24945986 | 0.95246378 |
| height | scovid_vs_pop | Weighted mode | 813 | 0.01487567 | 0.15159977 | 0.92185754 |
| interleukin 6 receptor \|\| id:prot-b-23 | scovid_vs_pop | Inverse variance weighted | 2 | -0.027792 | 0.03222711 | 0.38847794 |
| WHRadj | scovid_vs_pop | MR Egger | 317 | 0.13869219 | 0.26231871 | 0.59737456 |
| WHRadj | scovid_vs_pop | Weighted median | 317 | -0.1260267 | 0.16781147 | 0.4526516 |
| WHRadj | scovid_vs_pop | Inverse variance weighted | 317 | -0.0537452 | 0.10526259 | 0.60964338 |
| WHRadj | scovid_vs_pop | Simple mode | 317 | 0.00463215 | 0.46427064 | 0.99204572 |
| WHRadj | scovid_vs_pop | Weighted mode | 317 | -0.084851 | 0.27725841 | 0.75977889 |
