# Supplementary material for: Associations between body composition, fat distribution and metabolic consequences of excess adiposity with severe COVID-19 outcomes: observational study and Mendelian randomisation analysis
Source: Int J Obes (Lond). 2022 Jan 14;46(5):943–50. doi: 10.1038/s41366-021-01054-3 (PMC8758930; doi:10.1038/s41366-021-01054-3)
Supplement: Supplementary file 3 — Supplementary material 3 [file 41366_2021_1054_MOESM3_ESM.docx]

**Supplementary material 3**

| **Supplementary material 3. Egger test estimates from all exposures to the three COVID-19 outcomes** | | | | | | | | | | | |
| --- | --- | --- | --- | --- | --- | --- | --- | --- | --- | --- | --- |
| exposure | outcome | b | se | pval | nsnp | b_i | se_i | pval_i | Q | Q_df | Q_pval |
| Total bilirubin \|\| id:ukb-d-30840_irnt | covid_vs_pop | 0.08353301 | 0.05612473 | 0.139305 | 121 | -0.00388622 | 0.00193737 | 0.04713178 | 178.38196 | 119 | 0.00034966 |
| Total bilirubin \|\| id:ukb-d-30840_irnt | hcovid_vs_pop | 0.2682896 | 0.11211762 | 0.01830566 | 119 | -0.00805262 | 0.00380152 | 0.0362689 | 166.280152 | 117 | 0.00187851 |
| Total bilirubin \|\| id:ukb-d-30840_irnt | scovid_vs_pop | 0.48664128 | 0.15855739 | 0.00266357 | 120 | -0.01841447 | 0.00552457 | 0.00114734 | 152.526843 | 118 | 0.01778024 |
| Albumin \|\| id:ukb-d-30600_irnt | covid_vs_pop | -0.05422551 | 0.06662998 | 0.41678273 | 188 | 0.00023507 | 0.00175643 | 0.89367784 | 210.208142 | 186 | 0.10773042 |
| Albumin \|\| id:ukb-d-30600_irnt | hcovid_vs_pop | -0.07090936 | 0.14126559 | 0.61630393 | 184 | -0.0028339 | 0.0036877 | 0.44320275 | 218.137168 | 182 | 0.03469099 |
| Albumin \|\| id:ukb-d-30600_irnt | scovid_vs_pop | -0.0612099 | 0.19094178 | 0.74890128 | 186 | 7.11E-05 | 0.00515693 | 0.98901099 | 194.492745 | 184 | 0.28382799 |
| Total protein \|\| id:ukb-d-30860_irnt | covid_vs_pop | -0.04429576 | 0.07471995 | 0.55393094 | 214 | 0.00047678 | 0.00190757 | 0.80287332 | 249.127787 | 212 | 0.0409859 |
| Total protein \|\| id:ukb-d-30860_irnt | hcovid_vs_pop | -0.06685808 | 0.15033811 | 0.6569859 | 210 | 0.00154464 | 0.00377765 | 0.6830418 | 223.155035 | 208 | 0.2241595 |
| Total protein \|\| id:ukb-d-30860_irnt | scovid_vs_pop | 0.02263142 | 0.21040048 | 0.91444365 | 214 | 0.00360807 | 0.00544882 | 0.50857855 | 227.302496 | 212 | 0.22417028 |
| Cystatin C \|\| id:ukb-d-30720_irnt | covid_vs_pop | -0.09460722 | 0.05897686 | 0.10978663 | 288 | 0.00201602 | 0.00145189 | 0.16604979 | 331.472437 | 286 | 0.03314309 |
| Cystatin C \|\| id:ukb-d-30720_irnt | hcovid_vs_pop | -0.34532174 | 0.12204814 | 0.00499853 | 284 | 0.00880558 | 0.00298714 | 0.00346785 | 329.241848 | 282 | 0.02770831 |
| Cystatin C \|\| id:ukb-d-30720_irnt | scovid_vs_pop | -0.04851645 | 0.17787522 | 0.78523727 | 289 | 0.0026669 | 0.00438542 | 0.54358258 | 315.40831 | 287 | 0.11972962 |
| height | covid_vs_pop | 0.0851541 | 0.03906255 | 0.02954833 | 814 | -0.00071723 | 0.00085471 | 0.4016296 | 845.625207 | 812 | 0.20055623 |
| height | hcovid_vs_pop | 0.15382755 | 0.08042415 | 0.05613842 | 812 | -0.0030129 | 0.00177345 | 0.08972426 | 921.28317 | 810 | 0.00386218 |
| height | scovid_vs_pop | 0.04868582 | 0.12051186 | 0.68632517 | 813 | -0.00033716 | 0.00268011 | 0.89992022 | 924.683221 | 811 | 0.00329334 |
| Gamma glutamyltransferase \|\| id:ukb-d-30730_irnt | covid_vs_pop | -0.03553238 | 0.04739578 | 0.45413502 | 255 | 0.00108096 | 0.00145288 | 0.45755716 | 311.430111 | 253 | 0.00714412 |
| Gamma glutamyltransferase \|\| id:ukb-d-30730_irnt | hcovid_vs_pop | 0.02712718 | 0.09634946 | 0.77852251 | 251 | -0.00039476 | 0.00295213 | 0.89373141 | 307.160309 | 249 | 0.00701536 |
| Gamma glutamyltransferase \|\| id:ukb-d-30730_irnt | scovid_vs_pop | -0.03885883 | 0.14432876 | 0.78796404 | 257 | 0.0019735 | 0.004502 | 0.66149541 | 321.653428 | 255 | 0.00292125 |
| Urea \|\| id:ukb-d-30670_irnt | covid_vs_pop | -0.04724585 | 0.10872066 | 0.66450498 | 152 | 0.00116898 | 0.00270205 | 0.66590767 | 246.655477 | 150 | 1.11E-06 |
| Urea \|\| id:ukb-d-30670_irnt | hcovid_vs_pop | 0.08690474 | 0.19632123 | 0.65864499 | 152 | -0.00350029 | 0.00489843 | 0.47598353 | 199.665931 | 150 | 0.00416517 |
| Urea \|\| id:ukb-d-30670_irnt | scovid_vs_pop | 0.41245883 | 0.28644985 | 0.15196601 | 153 | -0.01044184 | 0.00719692 | 0.14888852 | 183.348482 | 151 | 0.03747793 |
| Body mass index \|\| id:ieu-a-2 | covid_vs_pop | 0.20556941 | 0.11609064 | 0.08071766 | 76 | -0.00106468 | 0.00324513 | 0.74377443 | 85.0970513 | 74 | 0.17764622 |
| Body mass index \|\| id:ieu-a-2 | hcovid_vs_pop | 0.3545189 | 0.21884479 | 0.10949588 | 76 | 0.00103187 | 0.00618997 | 0.86806057 | 81.5722776 | 74 | 0.25566339 |
| Body mass index \|\| id:ieu-a-2 | scovid_vs_pop | 0.12998272 | 0.30584913 | 0.67207746 | 76 | 0.00555205 | 0.00868166 | 0.52446281 | 73.3194659 | 74 | 0.50049274 |
| Phosphate \|\| id:ukb-d-30810_irnt | covid_vs_pop | -0.03494108 | 0.0657645 | 0.59607979 | 137 | 0.00142042 | 0.00191646 | 0.45987821 | 119.82071 | 135 | 0.821185 |
| Phosphate \|\| id:ukb-d-30810_irnt | hcovid_vs_pop | -0.1173295 | 0.15125046 | 0.43927541 | 136 | 0.00022888 | 0.00443938 | 0.95895869 | 178.028856 | 134 | 0.00655508 |
| Phosphate \|\| id:ukb-d-30810_irnt | scovid_vs_pop | 0.1050379 | 0.23573193 | 0.65661828 | 136 | -0.00185907 | 0.0069621 | 0.78985863 | 185.991662 | 134 | 0.00199947 |
| Alkaline phosphatase \|\| id:ukb-d-30610_irnt | covid_vs_pop | 0.04901459 | 0.0400563 | 0.22212324 | 280 | -0.00066969 | 0.00133055 | 0.61513947 | 356.014768 | 278 | 0.00107302 |
| Alkaline phosphatase \|\| id:ukb-d-30610_irnt | hcovid_vs_pop | 0.00882262 | 0.08231441 | 0.91472249 | 278 | 0.00322087 | 0.00274095 | 0.24097085 | 370.141916 | 276 | 0.00012983 |
| Alkaline phosphatase \|\| id:ukb-d-30610_irnt | scovid_vs_pop | 0.08343426 | 0.12764826 | 0.51388902 | 282 | -3.91E-05 | 0.00427823 | 0.99271604 | 393.708329 | 280 | 8.44E-06 |
| Apoliprotein A \|\| id:ukb-d-30630_irnt | covid_vs_pop | 0.08627228 | 0.04588115 | 0.06131973 | 233 | -0.00380958 | 0.00147615 | 0.01047715 | 302.060513 | 231 | 0.00115341 |
| Apoliprotein A \|\| id:ukb-d-30630_irnt | hcovid_vs_pop | 0.07948497 | 0.08689851 | 0.36132207 | 230 | -0.00296081 | 0.00281097 | 0.29331549 | 272.050812 | 228 | 0.02419172 |
| Apoliprotein A \|\| id:ukb-d-30630_irnt | scovid_vs_pop | 0.10973917 | 0.12915228 | 0.3963782 | 233 | -0.00268949 | 0.00421998 | 0.52454564 | 270.561744 | 231 | 0.03790791 |
| Testosterone \|\| id:ukb-d-30850_irnt | covid_vs_pop | -0.24550851 | 0.13257386 | 0.06743751 | 89 | 0.00550705 | 0.00223139 | 0.01554465 | 87.1200209 | 87 | 0.47621468 |
| Testosterone \|\| id:ukb-d-30850_irnt | hcovid_vs_pop | -0.58844038 | 0.27786513 | 0.03708556 | 88 | 0.0074412 | 0.00473346 | 0.11961289 | 98.6568231 | 86 | 0.16560347 |
| Testosterone \|\| id:ukb-d-30850_irnt | scovid_vs_pop | -1.01448486 | 0.45212874 | 0.02735504 | 90 | 0.01300642 | 0.00781293 | 0.09952336 | 123.036245 | 88 | 0.00812383 |
| Arm fat-free mass (left) \|\| id:ukb-b-19925 | covid_vs_pop | 0.09811757 | 0.10516413 | 0.35127851 | 496 | 0.00110094 | 0.00135112 | 0.41555903 | 545.401796 | 494 | 0.05448554 |
| Arm fat-free mass (left) \|\| id:ukb-b-19925 | hcovid_vs_pop | 0.06689413 | 0.21319026 | 0.75382391 | 493 | 0.00342595 | 0.00274743 | 0.21300549 | 546.283839 | 491 | 0.04243662 |
| Arm fat-free mass (left) \|\| id:ukb-b-19925 | scovid_vs_pop | -0.09811318 | 0.3313653 | 0.76728781 | 495 | 0.00447272 | 0.00430099 | 0.29888302 | 580.076662 | 493 | 0.00407544 |
| Arm fat-free mass (right) \|\| id:ukb-b-19520 | covid_vs_pop | 0.13070872 | 0.1024685 | 0.2026956 | 497 | 0.00045897 | 0.00129962 | 0.72412301 | 490.422992 | 495 | 0.5496239 |
| Arm fat-free mass (right) \|\| id:ukb-b-19520 | hcovid_vs_pop | 0.00574824 | 0.21207025 | 0.97838671 | 493 | 0.00380623 | 0.00269843 | 0.15901463 | 512.282158 | 491 | 0.24485506 |
| Arm fat-free mass (right) \|\| id:ukb-b-19520 | scovid_vs_pop | -0.28620087 | 0.33117572 | 0.38789857 | 497 | 0.00768817 | 0.00425134 | 0.07114914 | 553.975543 | 495 | 0.03394814 |
| Trunk fat-free mass \|\| id:ukb-b-17409 | covid_vs_pop | 0.10018641 | 0.08688587 | 0.24939029 | 539 | 0.00047985 | 0.00116327 | 0.68013814 | 536.65766 | 537 | 0.49605167 |
| Trunk fat-free mass \|\| id:ukb-b-17409 | hcovid_vs_pop | -0.00101434 | 0.17959478 | 0.99549571 | 537 | 0.00234048 | 0.0024095 | 0.33181011 | 563.076012 | 535 | 0.19378496 |
| Trunk fat-free mass \|\| id:ukb-b-17409 | scovid_vs_pop | -0.15563927 | 0.27484191 | 0.57143603 | 538 | 0.00419461 | 0.00370744 | 0.25839069 | 573.790965 | 536 | 0.12539988 |
| Whole body fat-free mass \|\| id:ukb-b-13354 | covid_vs_pop | 0.12058806 | 0.0899376 | 0.18056404 | 529 | 0.00049215 | 0.00120364 | 0.68278875 | 540.098801 | 527 | 0.33699832 |
| Whole body fat-free mass \|\| id:ukb-b-13354 | hcovid_vs_pop | 0.093561 | 0.18359187 | 0.61053682 | 525 | 0.00255697 | 0.00246573 | 0.30021387 | 549.129962 | 523 | 0.20742659 |
| Whole body fat-free mass \|\| id:ukb-b-13354 | scovid_vs_pop | -0.10329965 | 0.2895991 | 0.72146064 | 527 | 0.00485929 | 0.00391293 | 0.21484369 | 599.698471 | 525 | 0.01305679 |
| Alanine aminotransferase \|\| id:ukb-d-30620_irnt | covid_vs_pop | -0.17888356 | 0.07217745 | 0.01412415 | 181 | 0.00598053 | 0.00179318 | 0.00103642 | 191.668535 | 179 | 0.24527961 |
| Alanine aminotransferase \|\| id:ukb-d-30620_irnt | hcovid_vs_pop | -0.3399562 | 0.14776831 | 0.02258029 | 179 | 0.01042609 | 0.00365663 | 0.00487209 | 193.170191 | 177 | 0.19221676 |
| Alanine aminotransferase \|\| id:ukb-d-30620_irnt | scovid_vs_pop | -0.22960312 | 0.22945169 | 0.3183496 | 180 | 0.00727292 | 0.00568518 | 0.2024644 | 199.252948 | 178 | 0.13147148 |
| Aspartate aminotransferase \|\| id:ukb-d-30650_irnt | covid_vs_pop | -0.04337178 | 0.07886645 | 0.58292907 | 218 | 0.00115041 | 0.00199769 | 0.56530417 | 316.128229 | 216 | 1.04E-05 |
| Aspartate aminotransferase \|\| id:ukb-d-30650_irnt | hcovid_vs_pop | -0.1181497 | 0.14889385 | 0.42837703 | 211 | 0.00027715 | 0.00373821 | 0.94096972 | 254.932421 | 209 | 0.01648554 |
| Aspartate aminotransferase \|\| id:ukb-d-30650_irnt | scovid_vs_pop | -0.2111859 | 0.2355743 | 0.37098931 | 220 | -0.00096769 | 0.00597729 | 0.87153918 | 293.806353 | 218 | 0.00046691 |
| SHBG \|\| id:ukb-d-30830_irnt | covid_vs_pop | 0.03692269 | 0.04815896 | 0.44403906 | 237 | -0.00224243 | 0.00146001 | 0.12590897 | 259.58661 | 235 | 0.1298092 |
| SHBG \|\| id:ukb-d-30830_irnt | hcovid_vs_pop | 0.13408807 | 0.10392674 | 0.19825424 | 235 | -0.00401779 | 0.00316729 | 0.20587613 | 302.675121 | 233 | 0.00142864 |
| SHBG \|\| id:ukb-d-30830_irnt | scovid_vs_pop | 0.13320035 | 0.14821966 | 0.36975303 | 236 | -0.00186815 | 0.00454677 | 0.68154174 | 270.096113 | 234 | 0.05259765 |
| Creatinine \|\| id:ukb-d-30700_irnt | covid_vs_pop | -0.01195908 | 0.0760004 | 0.87506913 | 306 | 0.00017405 | 0.00163019 | 0.91504439 | 348.089903 | 304 | 0.04136903 |
| Creatinine \|\| id:ukb-d-30700_irnt | hcovid_vs_pop | -0.10502642 | 0.15617934 | 0.50180011 | 302 | 0.00434125 | 0.0033622 | 0.19763072 | 359.428576 | 300 | 0.01047042 |
| Creatinine \|\| id:ukb-d-30700_irnt | scovid_vs_pop | 0.23384021 | 0.23663686 | 0.32384695 | 307 | -0.00101172 | 0.00511966 | 0.84347862 | 356.34551 | 305 | 0.02279536 |
| Triglycerides \|\| id:ukb-d-30870_irnt | covid_vs_pop | 0.10217744 | 0.04397586 | 0.02110496 | 213 | -0.00016884 | 0.00147266 | 0.90883002 | 263.432784 | 211 | 0.00822018 |
| Triglycerides \|\| id:ukb-d-30870_irnt | hcovid_vs_pop | 0.17080961 | 0.08294631 | 0.04071305 | 210 | -0.00104181 | 0.00280009 | 0.71022377 | 235.606459 | 208 | 0.09172114 |
| Triglycerides \|\| id:ukb-d-30870_irnt | scovid_vs_pop | 0.26953571 | 0.12285322 | 0.0293297 | 213 | -0.00135641 | 0.0041699 | 0.74528713 | 230.509263 | 211 | 0.17000961 |
| Glycated haemoglobin \|\| id:ukb-d-30750_irnt | covid_vs_pop | -0.00349513 | 0.04997786 | 0.94429743 | 277 | 0.00118813 | 0.0014755 | 0.42137625 | 321.239228 | 275 | 0.02878823 |
| Glycated haemoglobin \|\| id:ukb-d-30750_irnt | hcovid_vs_pop | 0.0067105 | 0.09659294 | 0.94466522 | 273 | 0.00229347 | 0.00285341 | 0.42223809 | 291.262321 | 271 | 0.18996755 |
| Glycated haemoglobin \|\| id:ukb-d-30750_irnt | scovid_vs_pop | 0.1061004 | 0.15188619 | 0.48542454 | 276 | -0.00184453 | 0.00450744 | 0.68269817 | 311.552529 | 274 | 0.05886135 |
| WHRadj | covid_vs_pop | 0.12395314 | 0.08403574 | 0.14121315 | 316 | -0.00277413 | 0.00160533 | 0.08495732 | 355.079406 | 314 | 0.05489992 |
| WHRadj | hcovid_vs_pop | 0.14883485 | 0.16906339 | 0.37934334 | 316 | -0.002959 | 0.00321994 | 0.35882106 | 346.976361 | 314 | 0.09699815 |
| WHRadj | scovid_vs_pop | 0.13869219 | 0.26231871 | 0.59737456 | 317 | -0.00398825 | 0.00497908 | 0.42373516 | 344.009529 | 315 | 0.12539221 |
| Diastolic blood pressure, automated reading \|\| id:ukb-b-7992 | covid_vs_pop | -0.0769993 | 0.14341529 | 0.59182769 | 246 | -8.42E-05 | 0.00259767 | 0.97416494 | 338.186718 | 244 | 6.10E-05 |
| Diastolic blood pressure, automated reading \|\| id:ukb-b-7992 | hcovid_vs_pop | -0.10780222 | 0.26590619 | 0.68553254 | 243 | -0.00049268 | 0.0048305 | 0.91884674 | 283.378826 | 241 | 0.03162282 |
| Diastolic blood pressure, automated reading \|\| id:ukb-b-7992 | scovid_vs_pop | -0.56865398 | 0.42969079 | 0.18694017 | 246 | 0.00688947 | 0.00781659 | 0.37897378 | 321.231142 | 244 | 0.00065751 |
| Vitamin D \|\| id:ukb-d-30890_irnt | covid_vs_pop | -0.11337406 | 0.06299199 | 0.07747699 | 56 | 0.00196615 | 0.00272535 | 0.47375692 | 63.2713979 | 54 | 0.18167684 |
| Vitamin D \|\| id:ukb-d-30890_irnt | hcovid_vs_pop | -0.08739622 | 0.13201118 | 0.51087126 | 54 | 0.00343834 | 0.00554605 | 0.53798979 | 62.9392262 | 52 | 0.14233594 |
| Vitamin D \|\| id:ukb-d-30890_irnt | scovid_vs_pop | -0.08172589 | 0.18771402 | 0.66502593 | 56 | 0.00830923 | 0.00769134 | 0.28479423 | 55.0822565 | 54 | 0.43346761 |
| Arm fat mass (left) \|\| id:ukb-b-8338 | covid_vs_pop | 0.1769408 | 0.08479075 | 0.03753318 | 406 | -0.000235 | 0.00151043 | 0.87643806 | 404.776926 | 404 | 0.47975561 |
| Arm fat mass (left) \|\| id:ukb-b-8338 | hcovid_vs_pop | 0.37108331 | 0.17025253 | 0.02986646 | 404 | 0.00036767 | 0.00306162 | 0.90447204 | 422.539725 | 402 | 0.23092193 |
| Arm fat mass (left) \|\| id:ukb-b-8338 | scovid_vs_pop | 0.23558556 | 0.2549055 | 0.35593137 | 405 | 0.00124331 | 0.0046292 | 0.78839129 | 428.565312 | 403 | 0.18260125 |
| Arm fat percentage (left) \|\| id:ukb-b-20188 | covid_vs_pop | 0.26070488 | 0.150517 | 0.08407816 | 380 | -0.00032973 | 0.00200229 | 0.86928615 | 542.822012 | 378 | 5.26E-08 |
| Arm fat percentage (left) \|\| id:ukb-b-20188 | hcovid_vs_pop | 0.53135614 | 0.26422549 | 0.04503743 | 379 | 0.00094273 | 0.00354393 | 0.79037535 | 436.518892 | 377 | 0.01844702 |
| Arm fat percentage (left) \|\| id:ukb-b-20188 | scovid_vs_pop | -0.08921955 | 0.38456835 | 0.81666418 | 380 | 0.00915875 | 0.00519893 | 0.07893506 | 417.065313 | 378 | 0.08095929 |
| Arm fat percentage (right) \|\| id:ukb-b-12854 | covid_vs_pop | 0.26760334 | 0.14838304 | 0.07211588 | 378 | -0.00030541 | 0.00199058 | 0.87814274 | 529.841405 | 376 | 2.70E-07 |
| Arm fat percentage (right) \|\| id:ukb-b-12854 | hcovid_vs_pop | 0.56259254 | 0.26639623 | 0.03535902 | 377 | 0.00097769 | 0.00360007 | 0.78609849 | 443.940027 | 375 | 0.0081584 |
| Arm fat percentage (right) \|\| id:ukb-b-12854 | scovid_vs_pop | 0.10897044 | 0.39951649 | 0.78519067 | 378 | 0.00656608 | 0.00544887 | 0.22894792 | 448.949087 | 376 | 0.00571509 |
| Body fat percentage \|\| id:ukb-b-8909 | covid_vs_pop | 0.35250949 | 0.14330977 | 0.01435417 | 376 | -0.00165805 | 0.0019402 | 0.393334 | 419.371683 | 374 | 0.05257427 |
| Body fat percentage \|\| id:ukb-b-8909 | hcovid_vs_pop | 0.80661995 | 0.29107013 | 0.00586589 | 373 | -0.00245187 | 0.00396917 | 0.53713366 | 446.47056 | 371 | 0.00431482 |
| Body fat percentage \|\| id:ukb-b-8909 | scovid_vs_pop | 0.38621738 | 0.43345445 | 0.37349183 | 375 | 0.00407573 | 0.0059576 | 0.49432214 | 449.846473 | 373 | 0.00385296 |
| Leg fat mass (left) \|\| id:ukb-b-7212 | covid_vs_pop | 0.21620385 | 0.11212869 | 0.05453899 | 403 | 6.34E-05 | 0.00160051 | 0.96843358 | 427.415095 | 401 | 0.17452382 |
| Leg fat mass (left) \|\| id:ukb-b-7212 | hcovid_vs_pop | 0.50248366 | 0.2165061 | 0.02079561 | 402 | 0.00078127 | 0.0031168 | 0.80220222 | 411.609563 | 400 | 0.33357182 |
| Leg fat mass (left) \|\| id:ukb-b-7212 | scovid_vs_pop | 0.34413619 | 0.3187521 | 0.28095331 | 403 | 0.00226069 | 0.00463649 | 0.62610922 | 398.227637 | 401 | 0.52971953 |
| Leg fat percentage (right) \|\| id:ukb-b-20531 | covid_vs_pop | 0.35322377 | 0.18013975 | 0.05065872 | 367 | -0.00060947 | 0.00196642 | 0.75678318 | 396.967505 | 365 | 0.12003832 |
| Leg fat percentage (right) \|\| id:ukb-b-20531 | hcovid_vs_pop | 0.70635132 | 0.35490632 | 0.04731335 | 365 | 0.00171148 | 0.00390473 | 0.66142304 | 397.758582 | 363 | 0.10105758 |
| Leg fat percentage (right) \|\| id:ukb-b-20531 | scovid_vs_pop | 0.180247 | 0.5404456 | 0.73893721 | 366 | 0.00937512 | 0.00600622 | 0.11941566 | 417.174538 | 364 | 0.02828188 |
| Trunk fat mass \|\| id:ukb-b-20044 | covid_vs_pop | 0.25561563 | 0.08431427 | 0.00258884 | 405 | -0.00155152 | 0.00154449 | 0.31571577 | 399.329521 | 403 | 0.54227972 |
| Trunk fat mass \|\| id:ukb-b-20044 | hcovid_vs_pop | 0.60679241 | 0.17204202 | 0.0004689 | 403 | -0.00399787 | 0.00317052 | 0.2080588 | 423.812435 | 401 | 0.2077705 |
| Trunk fat mass \|\| id:ukb-b-20044 | scovid_vs_pop | 0.34192145 | 0.2659842 | 0.19936113 | 403 | 0.00124166 | 0.00494386 | 0.8018257 | 449.179043 | 401 | 0.04837147 |
| Trunk fat percentage \|\| id:ukb-b-16407 | covid_vs_pop | 0.34444613 | 0.11558324 | 0.00307273 | 371 | -0.00282268 | 0.00188589 | 0.13531661 | 384.218231 | 369 | 0.28192577 |
| Trunk fat percentage \|\| id:ukb-b-16407 | hcovid_vs_pop | 0.93084969 | 0.24116706 | 0.00013402 | 370 | -0.0068545 | 0.00396029 | 0.08432408 | 433.935888 | 368 | 0.01008935 |
| Trunk fat percentage \|\| id:ukb-b-16407 | scovid_vs_pop | 0.6894462 | 0.36879432 | 0.06235067 | 371 | -0.00214943 | 0.00610007 | 0.7247688 | 454.803095 | 369 | 0.00150663 |
| Whole body fat mass \|\| id:ukb-b-19393 | covid_vs_pop | 0.15253554 | 0.08999955 | 0.09086176 | 413 | 0.00073688 | 0.00158723 | 0.64271168 | 451.453847 | 411 | 0.08222566 |
| Whole body fat mass \|\| id:ukb-b-19393 | hcovid_vs_pop | 0.42759109 | 0.17364907 | 0.01421195 | 412 | 0.00042055 | 0.00308327 | 0.89157382 | 430.004618 | 410 | 0.23862483 |
| Whole body fat mass \|\| id:ukb-b-19393 | scovid_vs_pop | 0.27569188 | 0.26282699 | 0.29481992 | 412 | 0.00293098 | 0.00470642 | 0.53378687 | 438.005134 | 410 | 0.16361321 |
| Leg fat mass (right) \|\| id:ukb-b-18096 | covid_vs_pop | 0.15176614 | 0.11346357 | 0.18178534 | 407 | 0.0012966 | 0.00162718 | 0.42601093 | 445.215593 | 405 | 0.08195578 |
| Leg fat mass (right) \|\| id:ukb-b-18096 | hcovid_vs_pop | 0.47061484 | 0.21735437 | 0.03095596 | 407 | 0.00219026 | 0.00314461 | 0.48650755 | 424.37428 | 405 | 0.24399288 |
| Leg fat mass (right) \|\| id:ukb-b-18096 | scovid_vs_pop | 0.20746149 | 0.31951775 | 0.51651442 | 408 | 0.00623428 | 0.00467571 | 0.18317007 | 413.225239 | 406 | 0.39149249 |
| Leg fat percentage (left) \|\| id:ukb-b-18377 | covid_vs_pop | 0.39721502 | 0.18832449 | 0.03561883 | 361 | -0.00095437 | 0.00202607 | 0.63789418 | 403.280436 | 359 | 0.05329301 |
| Leg fat percentage (left) \|\| id:ukb-b-18377 | hcovid_vs_pop | 0.85337649 | 0.35414293 | 0.01646976 | 360 | 4.85E-05 | 0.00383988 | 0.9899372 | 367.871657 | 358 | 0.34810382 |
| Leg fat percentage (left) \|\| id:ukb-b-18377 | scovid_vs_pop | 0.38221283 | 0.54143996 | 0.48069753 | 360 | 0.00585439 | 0.00592778 | 0.32400663 | 382.848055 | 358 | 0.17547768 |
| Leg fat-free mass (left) \|\| id:ukb-b-16099 | covid_vs_pop | 0.16183392 | 0.09358715 | 0.08440383 | 487 | -3.63E-05 | 0.00125516 | 0.97694799 | 461.379808 | 485 | 0.77324517 |
| Leg fat-free mass (left) \|\| id:ukb-b-16099 | hcovid_vs_pop | 0.23661682 | 0.19407224 | 0.22335565 | 485 | -0.00032512 | 0.00262109 | 0.90133452 | 517.738247 | 483 | 0.13279272 |
| Leg fat-free mass (left) \|\| id:ukb-b-16099 | scovid_vs_pop | -0.11095938 | 0.31029454 | 0.72080325 | 486 | 0.00310857 | 0.00423242 | 0.46302114 | 589.987843 | 484 | 0.00067158 |
| Leg fat-free mass (right) \|\| id:ukb-b-12828 | covid_vs_pop | 0.09488665 | 0.09308448 | 0.30853481 | 492 | 0.00048058 | 0.00125941 | 0.70292641 | 456.324899 | 490 | 0.8599221 |
| Leg fat-free mass (right) \|\| id:ukb-b-12828 | hcovid_vs_pop | 0.10329901 | 0.19145115 | 0.58974846 | 489 | 0.00076004 | 0.00260747 | 0.77080319 | 509.45107 | 487 | 0.23272726 |
| Leg fat-free mass (right) \|\| id:ukb-b-12828 | scovid_vs_pop | -0.20019042 | 0.29830328 | 0.50247564 | 491 | 0.0032508 | 0.00410954 | 0.42930644 | 553.10753 | 489 | 0.0234208 |
| Hip circumference \|\| id:ukb-b-15590 | covid_vs_pop | 0.06814331 | 0.08504203 | 0.4234408 | 401 | 0.00148781 | 0.00155796 | 0.34016703 | 422.568346 | 399 | 0.19991761 |
| Hip circumference \|\| id:ukb-b-15590 | hcovid_vs_pop | 0.19670836 | 0.17478278 | 0.26108055 | 399 | 0.00279971 | 0.0032183 | 0.3848626 | 449.295138 | 397 | 0.03565108 |
| Hip circumference \|\| id:ukb-b-15590 | scovid_vs_pop | 0.10240858 | 0.26314173 | 0.6973537 | 400 | 0.00464703 | 0.00487317 | 0.34086774 | 454.427709 | 398 | 0.02643428 |
| Urate \|\| id:ukb-d-30880_irnt | covid_vs_pop | -0.07249724 | 0.05343154 | 0.1761905 | 228 | 0.00202718 | 0.00138414 | 0.14442626 | 273.238119 | 226 | 0.01723864 |
| Urate \|\| id:ukb-d-30880_irnt | hcovid_vs_pop | -0.09463574 | 0.10941573 | 0.38802115 | 223 | 0.00447725 | 0.00281246 | 0.11282869 | 269.998963 | 221 | 0.01359079 |
| Urate \|\| id:ukb-d-30880_irnt | scovid_vs_pop | -0.00446947 | 0.17211935 | 0.97930654 | 226 | 0.00097612 | 0.00441663 | 0.82528657 | 284.766733 | 224 | 0.003716 |
| Weight \|\| id:ukb-b-11842 | covid_vs_pop | 0.11324035 | 0.07787644 | 0.14657406 | 480 | 0.00095872 | 0.00130484 | 0.46285763 | 449.551264 | 478 | 0.82051775 |
| Weight \|\| id:ukb-b-11842 | hcovid_vs_pop | 0.25287837 | 0.1566653 | 0.10716162 | 478 | 0.00283446 | 0.00264647 | 0.28469708 | 485.53589 | 476 | 0.37123164 |
| Weight \|\| id:ukb-b-11842 | scovid_vs_pop | 0.29122385 | 0.24349053 | 0.23227604 | 479 | -0.00044592 | 0.00415852 | 0.91465102 | 520.984237 | 477 | 0.08017279 |
| Type 2 diabetes \|\| id:ebi-a-GCST006867 | covid_vs_pop | -0.05122401 | 0.03512367 | 0.14750703 | 115 | 0.00313338 | 0.00253144 | 0.21836232 | 125.143987 | 113 | 0.20481704 |
| Type 2 diabetes \|\| id:ebi-a-GCST006867 | hcovid_vs_pop | -0.07707034 | 0.06431925 | 0.23332802 | 115 | 0.00563404 | 0.00470365 | 0.23350092 | 112.730785 | 113 | 0.48944865 |
| Type 2 diabetes \|\| id:ebi-a-GCST006867 | scovid_vs_pop | -0.00415364 | 0.0939587 | 0.9648174 | 115 | -0.00132384 | 0.00698596 | 0.85004114 | 100.070885 | 113 | 0.80245146 |
| Cholesterol \|\| id:ukb-d-30690_irnt | covid_vs_pop | 0.12593787 | 0.05545816 | 0.02448063 | 163 | -0.00338925 | 0.00199428 | 0.09115891 | 245.382084 | 161 | 2.05E-05 |
| Cholesterol \|\| id:ukb-d-30690_irnt | hcovid_vs_pop | 0.07052149 | 0.10427469 | 0.49982961 | 161 | -0.00213645 | 0.00374554 | 0.56921311 | 212.397786 | 159 | 0.00298832 |
| Cholesterol \|\| id:ukb-d-30690_irnt | scovid_vs_pop | 0.03932239 | 0.14698916 | 0.78940989 | 164 | 0.00148921 | 0.00533498 | 0.78049336 | 187.489695 | 162 | 0.08309376 |
| Systolic blood pressure, automated reading \|\| id:ukb-b-20175 | covid_vs_pop | 0.15681336 | 0.13581363 | 0.24944885 | 231 | -0.00361723 | 0.0024387 | 0.13937968 | 264.331745 | 229 | 0.05438782 |
| Systolic blood pressure, automated reading \|\| id:ukb-b-20175 | hcovid_vs_pop | 0.39946505 | 0.25938815 | 0.12493414 | 231 | -0.00714293 | 0.00465114 | 0.12598307 | 242.295772 | 229 | 0.2608756 |
| Systolic blood pressure, automated reading \|\| id:ukb-b-20175 | scovid_vs_pop | 0.60747073 | 0.42066992 | 0.15008414 | 232 | -0.01324946 | 0.00751479 | 0.07920847 | 277.507112 | 230 | 0.01746354 |
| Apolipoprotein B \|\| id:ukb-d-30640_irnt | covid_vs_pop | 0.03908029 | 0.05411029 | 0.47130591 | 148 | -0.00237619 | 0.00191261 | 0.21608728 | 212.15848 | 146 | 0.00028789 |
| Apolipoprotein B \|\| id:ukb-d-30640_irnt | hcovid_vs_pop | 0.04235335 | 0.10272539 | 0.6807481 | 143 | -0.00161962 | 0.00361184 | 0.65453781 | 177.42604 | 141 | 0.02041835 |
| Apolipoprotein B \|\| id:ukb-d-30640_irnt | scovid_vs_pop | 0.08554815 | 0.1622374 | 0.59878405 | 148 | -0.00258788 | 0.00568125 | 0.64941702 | 190.231067 | 146 | 0.00812103 |
| HDL cholesterol \|\| id:ukb-d-30760_irnt | covid_vs_pop | 0.01996886 | 0.04592309 | 0.66405899 | 252 | -0.00216184 | 0.00147004 | 0.14265765 | 348.932628 | 250 | 3.51E-05 |
| HDL cholesterol \|\| id:ukb-d-30760_irnt | hcovid_vs_pop | -0.04652739 | 0.0825766 | 0.57364083 | 250 | -0.00037988 | 0.00267265 | 0.8870893 | 290.705128 | 248 | 0.03238305 |
| HDL cholesterol \|\| id:ukb-d-30760_irnt | scovid_vs_pop | -0.03636115 | 0.11807185 | 0.75837082 | 252 | 0.00075864 | 0.00385486 | 0.84414278 | 267.537058 | 250 | 0.21292664 |
| Arm fat mass (right) \|\| id:ukb-b-6704 | covid_vs_pop | 0.1592895 | 0.08497111 | 0.06155595 | 411 | 6.76E-05 | 0.00150103 | 0.96408152 | 421.945868 | 409 | 0.3187477 |
| Arm fat mass (right) \|\| id:ukb-b-6704 | hcovid_vs_pop | 0.31598805 | 0.16568261 | 0.0571981 | 410 | 0.00219183 | 0.00295667 | 0.45892719 | 417.018778 | 408 | 0.36816336 |
| Arm fat mass (right) \|\| id:ukb-b-6704 | scovid_vs_pop | 0.10535935 | 0.25520788 | 0.67994263 | 410 | 0.00511826 | 0.00461106 | 0.26765441 | 446.204166 | 408 | 0.09325492 |
| Calcium \|\| id:ukb-d-30680_irnt | covid_vs_pop | -0.02349651 | 0.07269711 | 0.74689276 | 191 | 0.00065145 | 0.00193739 | 0.73705573 | 177.646314 | 189 | 0.7128121 |
| Calcium \|\| id:ukb-d-30680_irnt | hcovid_vs_pop | -0.04857377 | 0.16414172 | 0.76761338 | 190 | 0.00359265 | 0.00438416 | 0.41356016 | 238.947671 | 188 | 0.00703634 |
| Calcium \|\| id:ukb-d-30680_irnt | scovid_vs_pop | -0.28848862 | 0.24560958 | 0.24163973 | 191 | 0.01168513 | 0.00658027 | 0.07737764 | 230.637374 | 189 | 0.02089563 |
| Insulin Resistance | covid_vs_pop | -0.05719911 | 0.14190246 | 0.68871168 | 49 | 0.0026705 | 0.00310849 | 0.39464659 | 37.8442622 | 47 | 0.82734499 |
| Insulin Resistance | hcovid_vs_pop | -0.08336059 | 0.27203605 | 0.76065838 | 48 | 0.00269831 | 0.00602929 | 0.65658994 | 46.5611778 | 46 | 0.44916289 |
| Insulin Resistance | scovid_vs_pop | -0.24166252 | 0.40672875 | 0.5552535 | 49 | 0.00516081 | 0.00919444 | 0.57726149 | 52.1454359 | 47 | 0.28071448 |
| Waist-to-hip ratio \|\| id:ieu-a-73 | covid_vs_pop | 0.35849047 | 0.33659392 | 0.29628408 | 29 | -0.00574391 | 0.0087346 | 0.51636006 | 29.111987 | 27 | 0.35548981 |
| Waist-to-hip ratio \|\| id:ieu-a-73 | hcovid_vs_pop | -0.31932572 | 0.62982023 | 0.61626326 | 29 | 0.01244678 | 0.0163958 | 0.45434311 | 14.7648818 | 27 | 0.97260191 |
| Waist-to-hip ratio \|\| id:ieu-a-73 | scovid_vs_pop | -0.78792744 | 0.92798228 | 0.40330473 | 29 | 0.01989969 | 0.02422816 | 0.41864306 | 23.2123739 | 27 | 0.67355195 |
| bmi | covid_vs_pop | 0.27060027 | 0.08374781 | 0.00131247 | 513 | -0.00146373 | 0.00134734 | 0.27781899 | 567.902826 | 511 | 0.04105813 |
| bmi | hcovid_vs_pop | 0.38665114 | 0.17227783 | 0.02523731 | 513 | 0.00033426 | 0.00278729 | 0.9045917 | 615.318084 | 511 | 0.00101848 |
| bmi | scovid_vs_pop | 0.46761611 | 0.25418905 | 0.06640112 | 513 | -0.00183945 | 0.00413807 | 0.65685588 | 595.212509 | 511 | 0.00582478 |
| Waist circumference \|\| id:ukb-b-9405 | covid_vs_pop | 0.18787926 | 0.11017706 | 0.08902535 | 356 | 0.00033997 | 0.00169359 | 0.8410174 | 364.717327 | 354 | 0.33586202 |
| Waist circumference \|\| id:ukb-b-9405 | hcovid_vs_pop | 0.43347842 | 0.21662428 | 0.04615101 | 355 | 0.00162619 | 0.003362 | 0.62889994 | 371.100838 | 353 | 0.24355437 |
| Waist circumference \|\| id:ukb-b-9405 | scovid_vs_pop | 0.28869833 | 0.34196847 | 0.39911545 | 355 | 0.00196859 | 0.00533916 | 0.71256598 | 408.137868 | 353 | 0.02270874 |
| IGF-1 \|\| id:ukb-d-30770_irnt | covid_vs_pop | -0.06088593 | 0.05037552 | 0.22769027 | 323 | 0.00216981 | 0.00136738 | 0.1135327 | 347.830961 | 321 | 0.14527262 |
| IGF-1 \|\| id:ukb-d-30770_irnt | hcovid_vs_pop | -0.12834679 | 0.10917948 | 0.24066223 | 316 | 0.00187576 | 0.00299502 | 0.53157836 | 405.991547 | 314 | 0.00035019 |
| IGF-1 \|\| id:ukb-d-30770_irnt | scovid_vs_pop | -0.05274932 | 0.1656766 | 0.75039827 | 323 | 0.00046658 | 0.00459093 | 0.91911382 | 428.602374 | 321 | 5.37E-05 |
| Glucose \|\| id:ukb-d-30740_irnt | covid_vs_pop | -0.12762882 | 0.09092778 | 0.16349671 | 103 | 0.00342717 | 0.00277701 | 0.22002093 | 167.71149 | 101 | 3.44E-05 |
| Glucose \|\| id:ukb-d-30740_irnt | hcovid_vs_pop | -0.03293038 | 0.1670223 | 0.84410126 | 102 | 0.00074745 | 0.00510429 | 0.88387208 | 139.618266 | 100 | 0.00547033 |
| Glucose \|\| id:ukb-d-30740_irnt | scovid_vs_pop | 0.13586209 | 0.23776114 | 0.56899532 | 102 | 0.00333517 | 0.00728903 | 0.64826095 | 122.517615 | 100 | 0.06268007 |
| C-reactive protein \|\| id:ukb-d-30710_irnt | covid_vs_pop | 0.02557456 | 0.05546854 | 0.64529413 | 187 | 0.00185465 | 0.00172703 | 0.28426733 | 268.44374 | 185 | 5.89E-05 |
| C-reactive protein \|\| id:ukb-d-30710_irnt | hcovid_vs_pop | 0.03027163 | 0.10168516 | 0.76627019 | 185 | 0.00376521 | 0.00315901 | 0.23484553 | 220.407017 | 183 | 0.03072133 |
| C-reactive protein \|\| id:ukb-d-30710_irnt | scovid_vs_pop | 0.03467709 | 0.14445267 | 0.81055072 | 187 | 0.00517838 | 0.00453104 | 0.25457004 | 195.209403 | 185 | 0.28916984 |
| Direct bilirubin \|\| id:ukb-d-30660_irnt | covid_vs_pop | -0.02839723 | 0.05549648 | 0.61038865 | 76 | 0.00024643 | 0.00263924 | 0.92586049 | 121.69837 | 74 | 0.00040177 |
| Direct bilirubin \|\| id:ukb-d-30660_irnt | hcovid_vs_pop | -0.03626707 | 0.10041976 | 0.71907149 | 72 | 0.00310612 | 0.00467843 | 0.50892025 | 90.0578163 | 70 | 0.05358384 |
| Direct bilirubin \|\| id:ukb-d-30660_irnt | scovid_vs_pop | 0.07398572 | 0.14501495 | 0.61139432 | 78 | -0.00447495 | 0.00680116 | 0.51254677 | 92.0168355 | 76 | 0.10188358 |
| Adiponectin | covid_vs_pop | 0.0126294 | 0.06367066 | 0.84543057 | 17 | -0.00504229 | 0.00572797 | 0.39258644 | 29.6920011 | 15 | 0.01308187 |
| Adiponectin | hcovid_vs_pop | -0.04247919 | 0.10679369 | 0.69640411 | 17 | 0.00130701 | 0.00965981 | 0.89417062 | 22.8737031 | 15 | 0.08685843 |
| Adiponectin | scovid_vs_pop | -0.03564189 | 0.15704471 | 0.82352375 | 17 | 0.00510406 | 0.01428711 | 0.72588162 | 23.3048401 | 15 | 0.07787853 |
| LDL direct \|\| id:ukb-d-30780_irnt | covid_vs_pop | 0.07184043 | 0.05951362 | 0.22940486 | 143 | -0.00136075 | 0.00206303 | 0.51059215 | 225.886089 | 141 | 7.39E-06 |
| LDL direct \|\| id:ukb-d-30780_irnt | hcovid_vs_pop | 0.06568375 | 0.11132887 | 0.55615721 | 140 | -0.0001569 | 0.00380359 | 0.96715507 | 181.292745 | 138 | 0.00789654 |
| LDL direct \|\| id:ukb-d-30780_irnt | scovid_vs_pop | -0.10688994 | 0.15133651 | 0.48113885 | 146 | 0.00321638 | 0.0054015 | 0.55247065 | 173.615679 | 144 | 0.04679822 |
| Lipoprotein A \|\| id:ukb-d-30790_irnt | covid_vs_pop | -0.09149096 | 0.09193705 | 0.33930814 | 14 | -0.00247221 | 0.00962777 | 0.80169916 | 20.0436236 | 12 | 0.06626524 |
| Lipoprotein A \|\| id:ukb-d-30790_irnt | hcovid_vs_pop | 0.1379259 | 0.22471903 | 0.55184833 | 13 | -0.02747048 | 0.02380403 | 0.27294654 | 28.0973919 | 11 | 0.00312797 |
| Lipoprotein A \|\| id:ukb-d-30790_irnt | scovid_vs_pop | 0.4074746 | 0.25490059 | 0.13822336 | 13 | -0.07014154 | 0.02659767 | 0.02311124 | 14.5465637 | 11 | 0.20421038 |
