# Supplementary material for: Associations between body composition, fat distribution and metabolic consequences of excess adiposity with severe COVID-19 outcomes: observational study and Mendelian randomisation analysis
Source: Int J Obes (Lond). 2022 Jan 14;46(5):943–50. doi: 10.1038/s41366-021-01054-3 (PMC8758930; doi:10.1038/s41366-021-01054-3)
Supplement: Supplementary file 4 — Supplementary information [file 41366_2021_1054_MOESM4_ESM.pdf]

Supplementary

Tables

Table S1. Details of GWAS summary data
Table S2. Descriptive characteristics of the included population in the UK Biobank study
Table S3a. Association between BMI z-score and COVID-19 outcomes with sequential adjustments in the UK Biobank study
Table S3b. Association between FMI z-score and COVID-19 outcomes with sequential adjustments in the UK Biobank study
Table S3c. Association between SMMI z-score and COVID-19 outcomes with sequential adjustments in the UK Biobank study
Table S3d. Association between WHR z-score and COVID-19 outcomes with sequential adjustments in the UK Biobank study
Table S3e. Association between metabolic disturbance and COVID-19 outcomes with sequential adjustments in the UK Biobank study
Table S3f. Association between type 2 diabetes and COVID-19 outcomes with sequential adjustments in the UK Biobank study
Table S4. Associations of adiposity markers with COVID-19 outcomes with correction for regression dilution bias in the UK Biobank study

Figures

Figure S1. DAG for the association between BMI, body fat distribution, and conditions caused by metabolic disturbance and incidence of severe COVID-19 outcomes
Figure S2a. Restricted cubic spline models for BMI z scores showing the hazard ratio for its association with COVID-19 outcomes
Figure S2b. Restricted cubic spline models for FMI z scores showing the hazard ratio for its association with COVID-19 outcomes
Figure S2c. Restricted cubic spline models for SMMI z scores showing the hazard ratio for its association with COVID-19 outcomes
Figure S2d. Restricted cubic spline models for WHR z scores showing the hazard ratio for its association with COVID-19 outcomes
Figure S3a. The causal effect estimates on COVID-19 test across different MR methods
Figure S3b. The causal effect estimates on COVID-19 hospital admission across different MR methods
Figure S3c. The causal effect estimates on very severe confirmed COVID-19 across different MR methods
Figure S4a. Effect modification of the associations between BMI and COVID-19 outcomes by age, gender, ethnicity, and cardiovascular disease
Figure S4b. Effect modification of the associations between FMI and COVID-19 outcomes by age, gender, ethnicity, and cardiovascular disease
Figure S4c. Effect modification of the associations between WHR and COVID-19 outcomes by age, gender, ethnicity, and cardiovascular disease

Figure S4d. Effect modification of the associations between metabolic disturbance and COVID-19 outcomes by age, gender, ethnicity, and cardiovascular disease
Figure S4e. Effect modification of the associations between type 2 diabetes and COVID-19 outcomes by age, gender, ethnicity, and cardiovascular disease
Figure S5. Multivariable MR analysis assessing the causal effect estimates of BMI with individually adjusting for the risk factors listed on the y-axis
Figure S6. Multivariate MR analysis assessing the comparative causal role of each individual risk factor (listed on the y-axis) on the risk of COVID-19 conditional on body mass index

Table S1. Details of GWAS summary data.

	Type	Category	Full Name	MR-BASE ID	Consortium	Year	Population	Sample_size	Ncase	Ncontrol	PMID
1	Outcome		covid vs. population		COVID19HG	2021	European	1683768	38984	1644784	NA
2	Outcome		hospitalized covid vs. population		COVID19HG	2021	European	1887658	9986	1877672	NA
3	Outcome		very severe respiratory confirmed covid vs. population		COVID19HG	2021	European	1388342	5101	1383241	NA
1	Exposure	Total adiposity	BMI (GIANT+UKBB)		GIANT	2018	European	681275	NA	NA	30124842
2	Exposure	Total adiposity	Body fat percentage	ukb-b-8909	MRC-IEU	2018	European	454633	NA	NA	NA
3	Exposure	Lean mass	Whole body fat-free mass	ukb-b-13354	MRC-IEU	2018	European	454850	NA	NA	NA
4	Exposure	Lean mass	Arm fat-free mass (left)	ukb-b-19925	MRC-IEU	2018	European	454672	NA	NA	NA
5	Exposure	Lean mass	Leg fat-free mass (left)	ukb-b-16099	MRC-IEU	2018	European	454805	NA	NA	NA
6	Exposure	Fat mass distribution	Arm fat mass (left)	ukb-b-8338	MRC-IEU	2018	European	454684	NA	NA	NA
7	Exposure	Fat mass distribution	Leg fat mass (left)	ukb-b-7212	MRC-IEU	2018	European	454823	NA	NA	NA
8	Exposure	Fat mass distribution	Trunk fat mass	ukb-b-20044	MRC-IEU	2018	European	454588	NA	NA	NA
9	Exposure	Fat mass distribution	Waist circumference	ukb-b-9405	MRC-IEU	2018	European	462166	NA	NA	NA
10	Exposure	Fat mass distribution	Hip circumference	ukb-b-15590	MRC-IEU	2018	European	462117	NA	NA	NA
11	Exposure	Fat mass distribution	Waist-to-hip ratio	ieu-a-73	NA	2015	European	212244	NA	NA	25673412
12	Exposure	Fat mass distribution	WHRadjBMI		GIANT	2019	European	694649	NA	NA	30239722
13	Exposure	Metabolic biomarkers	Insulin Resistance		NA	2017	European	188577	NA	NA	27841877
14	Exposure	Metabolic biomarkers	IGF-1	ukb-d-30770_irnt	NEALE LAB	2018	European	342439	NA	NA	NA
15	Exposure	Metabolic biomarkers	Glucose	ukb-d-30740_irnt	NEALE LAB	2018	European	314916	NA	NA	NA
16	Exposure	Metabolic biomarkers	Glycated haemoglobin	ukb-d-30750_irnt	NEALE LAB	2018	European	344182	NA	NA	NA
17	Exposure	Metabolic biomarkers	Adiponectin		NA	2019	Mixed	67739	NA	NA	31178129
18	Exposure	Metabolic biomarkers	Apolipoprotein A	ukb-d-30630_irnt	NEALE LAB	2018	European	313387	NA	NA	NA
19	Exposure	Metabolic biomarkers	HDL cholesterol	ukb-d-30760_irnt	NEALE LAB	2018	European	315133	NA	NA	NA
20	Exposure	Metabolic biomarkers	Apolipoprotein B	ukb-d-30640_irnt	NEALE LAB	2018	European	342590	NA	NA	NA
21	Exposure	Metabolic biomarkers	LDL	ukb-d-30780_irnt	NEALE LAB	2018	European	343621	NA	NA	NA
22	Exposure	Metabolic biomarkers	Triglycerides	ukb-d-30870_irnt	NEALE LAB	2018	European	343992	NA	NA	NA
23	Exposure	Diabetes	Type 2 diabetes	ebi-a-GCST006867	NA	2018	European	655666	61714	593952	30054458

Note: COVID19HG Covid19 Host Genetics Initiative; GIANT international Genetic Investigation of ANthropometric Traits; MRC-IEU MRC Integrative Epidemiology Unit; NA: not applicable

Table S2. Descriptive characteristics of the included population in the UK Biobank study

	All	COVID-19 test			COVID-19 hospital admission			COVID-19 ICU admission			COVID-19 death		
		No	Yes	p-value	No	Yes	p-value	No	Yes	p-value	No	Yes	p-value
N	435504	429938	5566	<0.001	434937	567	<0.001	435397	107	<0.001	435138	366	<0.001
Exposures													
BMI, mean (SD)	27.4 (4.8)	27.4 (4.8)	28.8 (5.3)	<0.001	27.4 (4.8)	29.6 (5.2)	<0.001	27.4 (4.8)	30.7 (5.2)	<0.001	27.4 (4.8)	29.7 (5.8)	<0.001
FMI, mean (SD)	8.8 (3.6)	8.8 (3.6)	9.5 (4.0)	<0.001	8.8 (3.6)	9.8 (3.9)	<0.001	8.8 (3.6)	10.2 (4.2)	<0.001	8.8 (3.6)	9.9 (4.5)	<0.001
SMMI, mean (SD)	7.8 (1.3)	7.8 (1.3)	8.1 (1.4)	<0.001	7.8 (1.3)	8.4 (1.4)	<0.001	7.8 (1.3)	8.8 (1.4)	<0.001	7.8 (1.3)	8.4 (1.5)	<0.001
WHR, mean (SD)	0.9 (0.1)	0.9 (0.1)	0.9 (0.1)	<0.001	0.9 (0.1)	0.9 (0.1)	<0.001	0.9 (0.1)	0.9 (0.1)	<0.001	0.9 (0.1)	0.9 (0.1)	<0.001
Metabolic disturbance	55232 (12.7%)	54821 (12.6%)	411 (25.0%)	<0.001	55039 (12.7%)	193 (34.3%)	<0.001	55198 (12.7%)	34 (32.7%)	<0.001	55095 (12.7%)	137 (37.6%)	<0.001
Type 2 diabetes	40266 (9.2%)	39941 (9.2%)	325 (19.7%)	<0.001	40120 (9.2%)	146 (26.0%)	<0.001	40242 (9.2%)	24 (23.1%)	<0.001	40153 (9.2%)	113 (31.0%)	<0.001
Demographics													
Age, mean (SD)	67.5 (8.1)	67.5 (8.1)	67.0 (9.2)	0.012	67.5 (8.1)	69.8 (8.3)	<0.001	67.5 (8.1)	67.2 (8.0)	0.74	67.5 (8.1)	73.6 (6.1)	<0.001
Sex				<0.001			<0.001			<0.001			<0.001
Female	237317 (54.5%)	236531 (54.5%)	786 (47.8%)		237105 (54.5%)	212 (37.7%)		237281 (54.5%)	36 (34.6%)		237186 (54.5%)	131 (36.0%)	
Male	198187 (45.5%)	197327 (45.5%)	860 (52.2%)		197837 (45.5%)	350 (62.3%)		198119 (45.5%)	68 (65.4%)		197954 (45.5%)	233 (64.0%)	
Ethnicity				<0.001			<0.001			<0.001			<0.001
White	408458 (93.8%)	407034 (93.8%)	1424 (86.5%)		407984 (93.8%)	474 (84.3%)		408374 (93.8%)	84 (80.8%)		408134 (93.8%)	324 (89.0%)	
Black	7609 (1.7%)	7523 (1.7%)	86 (5.2%)		7566 (1.7%)	43 (7.7%)		7599 (1.7%)	10 (9.6%)		7587 (1.7%)	22 (6.0%)	
Asian	10468 (2.4%)	10387 (2.4%)	81 (4.9%)		10440 (2.4%)	28 (5.0%)		10462 (2.4%)	6 (5.8%)		10458 (2.4%)	10 (2.7%)	
Others	6829 (1.6%)	6782 (1.6%)	47 (2.9%)		6815 (1.6%)	14 (2.5%)		6826 (1.6%)	3 (2.9%)		6824 (1.6%)	5 (1.4%)	
Missing	2140 (0.5%)	2132 (0.5%)	8 (0.5%)		2137 (0.5%)	3 (0.5%)		2139 (0.5%)	1 (1.0%)		2137 (0.5%)	3 (0.8%)	
Townsend index (quintiles)				<0.001			<0.001			<0.001			<0.001
Q1	86368 (19.8%)	86138 (19.9%)	230 (14.0%)		86287 (19.8%)	81 (14.4%)		86354 (19.8%)	14 (13.5%)		86311 (19.8%)	57 (15.7%)	
Q2	88080 (20.2%)	87806 (20.2%)	274 (16.6%)		87998 (20.2%)	82 (14.6%)		88067 (20.2%)	13 (12.5%)		88024 (20.2%)	56 (15.4%)	
Q3	87417 (20.1%)	87130 (20.1%)	287 (17.4%)		87336 (20.1%)	81 (14.4%)		87399 (20.1%)	18 (17.3%)		87358 (20.1%)	59 (16.2%)	
Q4	87213 (20.0%)	86865 (20.0%)	348 (21.1%)		87095 (20.0%)	118 (21.0%)		87192 (20.0%)	21 (20.2%)		87143 (20.0%)	70 (19.2%)	
Q5	85928 (19.7%)	85422 (19.7%)	506 (30.7%)		85728 (19.7%)	200 (35.6%)		85890 (19.7%)	38 (36.5%)		85807 (19.7%)	121 (33.2%)	

Missing	498 (0.1%)	497 (0.1%)	1 (0.1%)		498 (0.1%)	0 (0.0%)		498 (0.1%)	0 (0.0%)		497 (0.1%)	1 (0.3%)	
Education				<0.001			<0.001			0.057			<0.001
None of the above	72498 (16.6%)	72100 (16.6%)	398 (24.2%)		72331 (16.6%)	167 (29.7%)		72477 (16.6%)	21 (20.2%)		72372 (16.6%)	126 (34.6%)	
Vocational qualification (NVQ, HND or HNC)	61783 (14.2%)	61523 (14.2%)	260 (15.8%)		61703 (14.2%)	80 (14.2%)		61771 (14.2%)	12 (11.5%)		61738 (14.2%)	45 (12.4%)	
Any school degree (A-level, AS-level, O-level, GCSE, CSE)	123397 (28.3%)	123043 (28.4%)	354 (21.5%)		123286 (28.3%)	111 (19.8%)		123367 (28.3%)	30 (28.8%)		123334 (28.3%)	63 (17.3%)	
Higher degree (college, university or professional degree/qualification)	168977 (38.8%)	168385 (38.8%)	592 (36.0%)		168788 (38.8%)	189 (33.6%)		168942 (38.8%)	35 (33.7%)		168862 (38.8%)	115 (31.6%)	
Missing	8849 (2.0%)	8807 (2.0%)	42 (2.6%)		8834 (2.0%)	15 (2.7%)		8843 (2.0%)	6 (5.8%)		8834 (2.0%)	15 (4.1%)	
Health behaviour													
Smoking status				<0.001			<0.001			0.007			<0.001
Never	237035 (54.4%)	236236 (54.5%)	799 (48.5%)		236779 (54.4%)	256 (45.6%)		236995 (54.4%)	40 (38.5%)		236900 (54.4%)	135 (37.1%)	
Previous	151375 (34.8%)	150735 (34.7%)	640 (38.9%)		151139 (34.7%)	236 (42.0%)		151323 (34.8%)	52 (50.0%)		151204 (34.7%)	171 (47.0%)	
Current	44842 (10.3%)	44651 (10.3%)	191 (11.6%)		44781 (10.3%)	61 (10.9%)		44831 (10.3%)	11 (10.6%)		44788 (10.3%)	54 (14.8%)	
Missing	2252 (0.5%)	2236 (0.5%)	16 (1.0%)		2243 (0.5%)	9 (1.6%)		2251 (0.5%)	1 (1.0%)		2248 (0.5%)	4 (1.1%)	
Physical activity(MET-minutes per week)				<0.001			<0.001			0.50			0.25
Low (<600)	98517 (22.6%)	98094 (22.6%)	423 (25.7%)		98348 (22.6%)	169 (30.1%)		98491 (22.6%)	26 (25.0%)		98424 (22.6%)	93 (25.5%)	
Moderate(≥600 and <3000)	201447 (46.3%)	200782 (46.3%)	665 (40.4%)		201233 (46.3%)	214 (38.1%)		201406 (46.3%)	41 (39.4%)		201294 (46.3%)	153 (42.0%)	
High(≥ 3000)	118011 (27.1%)	117545 (27.1%)	466 (28.3%)		117864 (27.1%)	147 (26.2%)		117980 (27.1%)	31 (29.8%)		117912 (27.1%)	99 (27.2%)	
Missing	17529 (4.0%)	17437 (4.0%)	92 (5.6%)		17497 (4.0%)	32 (5.7%)		17523 (4.0%)	6 (5.8%)		17510 (4.0%)	19 (5.2%)	
Alcohol intake group(unit/week)				<0.001			<0.001			0.23			<0.001
None	11613 (2.7%)	11558 (2.7%)	55 (3.3%)		11589 (2.7%)	24 (4.3%)		11610 (2.7%)	3 (2.9%)		11600 (2.7%)	13 (3.6%)	
Occasional (<1)	8916 (2.0%)	8875 (2.0%)	41 (2.5%)		8908 (2.0%)	8 (1.4%)		8915 (2.0%)	1 (1.0%)		8907 (2.0%)	9 (2.5%)	
Moderate (1-14)	134150 (30.8%)	133717 (30.8%)	433 (26.3%)		134012 (30.8%)	138 (24.6%)		134127 (30.8%)	23 (22.1%)		134070 (30.8%)	80 (22.0%)	
Heavy (>14)	185420 (42.6%)	184788 (42.6%)	632 (38.4%)		185197 (42.6%)	223 (39.7%)		185373 (42.6%)	47 (45.2%)		185263 (42.6%)	157 (43.1%)	
Missing	95405 (21.9%)	94920 (21.9%)	485 (29.5%)		95236 (21.9%)	169 (30.1%)		95375 (21.9%)	30 (28.8%)		95300 (21.9%)	105 (28.8%)	
Fruit & vegetables intake(servings/day)				<0.001			0.023			0.16			0.033

0-1	11371 (2.6%)	11304 (2.6%)	67 (4.1%)		11344 (2.6%)	27 (4.8%)		11365 (2.6%)	6 (5.8%)		11357 (2.6%)	14 (3.8%)	
1-2	43695 (10.0%)	43509 (10.0%)	186 (11.3%)		43636 (10.0%)	59 (10.5%)		43688 (10.0%)	7 (6.7%)		43663 (10.0%)	32 (8.8%)	
2-5	268188 (61.6%)	267229 (61.6%)	959 (58.3%)		267855 (61.6%)	333 (59.3%)		268119 (61.6%)	69 (66.3%)		267983 (61.6%)	205 (56.3%)	
5+	110862 (25.5%)	110435 (25.5%)	427 (25.9%)		110720 (25.5%)	142 (25.3%)		110840 (25.5%)	22 (21.2%)		110752 (25.5%)	110 (30.2%)	
Missing	1388 (0.3%)	1381 (0.3%)	7 (0.4%)		1387 (0.3%)	1 (0.2%)		1388 (0.3%)	0 (0.0%)		1385 (0.3%)	3 (0.8%)	
Non-obesity-related morbidity (with)													
COPD	17448 (4.0%)	17289 (4.0%)	159 (9.7%)	<0.001	17361 (4.0%)	87 (15.5%)	<0.001	17437 (4.0%)	11 (10.6%)	<0.001	17385 (4.0%)	63 (17.3%)	<0.001
Asthma	62666 (14.4%)	62356 (14.4%)	310 (18.8%)	<0.001	62541 (14.4%)	125 (22.2%)	<0.001	62641 (14.4%)	25 (24.0%)	0.005	62602 (14.4%)	64 (17.6%)	0.082
Autoimmune condition	10425 (2.4%)	10375 (2.4%)	50 (3.0%)	0.087	10402 (2.4%)	23 (4.1%)	0.008	10420 (2.4%)	5 (4.8%)	0.11	10409 (2.4%)	16 (4.4%)	0.012
Colitis	4990 (1.1%)	4959 (1.1%)	31 (1.9%)	0.005	4975 (1.1%)	15 (2.7%)	<0.001	4987 (1.1%)	3 (2.9%)	0.096	4982 (1.1%)	8 (2.2%)	0.059
Crohn disease	2601 (0.6%)	2587 (0.6%)	14 (0.9%)	0.18	2591 (0.6%)	10 (1.8%)	<0.001	2599 (0.6%)	2 (1.9%)	0.079	2597 (0.6%)	4 (1.1%)	0.21
Obesity-related morbidity(with)													
Hypertension	254659 (58.5%)	253554 (58.4%)	1105 (67.1%)	<0.001	254220 (58.4%)	439 (78.1%)	<0.001	254576 (58.5%)	83 (79.8%)	<0.001	254341 (58.5%)	318 (87.4%)	<0.001
CVD	186112 (42.7%)	185031 (42.6%)	1081 (65.7%)	<0.001	185653 (42.7%)	459 (81.7%)	<0.001	186022 (42.7%)	90 (86.5%)	<0.001	185798 (42.7%)	314 (86.3%)	<0.001
Reflux	58281 (13.4%)	57959 (13.4%)	322 (19.6%)	<0.001	58140 (13.4%)	141 (25.1%)	<0.001	58258 (13.4%)	23 (22.1%)	0.009	58208 (13.4%)	73 (20.1%)	<0.001
Sleep apnoea	8789 (2.0%)	8728 (2.0%)	61 (3.7%)	<0.001	8758 (2.0%)	31 (5.5%)	<0.001	8781 (2.0%)	8 (7.7%)	<0.001	8765 (2.0%)	24 (6.6%)	<0.001

Note: BMI body mass index; FMI fat mass index; SMMI skeletal muscle mass index; WHR Waist to hip ratio; ICU intensive care unit

Table S3a. Association between BMI z-score and COVID-19 outcomes with sequential adjustments in the UK Biobank study

Model and adjustment	Unweighted HR (95% CI)	Weighted HR (95% CI)
COVID-19 test		
Model 1=Age and gender	1.31 (1.25 to 1.37)	1.21 (1.16 to 1.27)
Model 2=Model 1+demographics	1.26 (1.20 to 1.32)	1.19 (1.13 to 1.24)
Model 3=Model 2+behavioural risks	1.23 (1.18 to 1.29)	1.17 (1.12 to 1.22)
Model 4=Model 3+Non-obesity-related morbidity	1.22 (1.16 to 1.28)	1.16 (1.11 to 1.22)
Model 5=Model 4+obesity-related morbidity	1.14 (1.09 to 1.20)	1.13 (1.07 to 1.19)
COVID-19 hospital admission		
Model 1=Age and gender	1.49 (1.38 to 1.6)	1.39 (1.30 to 1.48)
Model 2=Model 1+demographics	1.40 (1.30 to 1.51)	1.34 (1.26 to 1.44)
Model 3=Model 2+behavioural risks	1.36 (1.26 to 1.47)	1.31 (1.22 to 1.41)
Model 4=Model 3+Non-obesity-related morbidity	1.33 (1.23 to 1.43)	1.29 (1.20 to 1.39)
Model 5=Model 4+obesity-related morbidity	1.21 (1.11 to 1.31)	1.21 (1.11 to 1.31)
COVID-19 ICU admission		
Model 1=Age and gender	1.69 (1.46 to 1.97)	1.61 (1.44 to 1.80)
Model 2=Model 1+demographics	1.63 (1.40 to 1.90)	1.58 (1.41 to 1.77)
Model 3=Model 2+behavioural risks	1.62 (1.38 to 1.90)	1.58 (1.39 to 1.80)
Model 4=Model 3+Non-obesity-related morbidity	1.59 (1.35 to 1.86)	1.55 (1.36 to 1.77)
Model 5=Model 4+obesity-related morbidity	1.38 (1.16 to 1.65)	1.38 (1.20 to 1.58)
COVID-19 death		
Model 1=Age and gender	1.52 (1.38 to 1.66)	1.44 (1.30 to 1.60)
Model 2=Model 1+demographics	1.43 (1.30 to 1.57)	1.39 (1.25 to 1.54)
Model 3=Model 2+behavioural risks	1.41 (1.28 to 1.56)	1.37 (1.23 to 1.53)
Model 4=Model 3+Non-obesity-related morbidity	1.39 (1.26 to 1.53)	1.36 (1.22 to 1.51)
Model 5=Model 4+obesity-related morbidity	1.23 (1.11 to 1.37)	1.23 (1.09 to 1.39)

Note: Estimates are hazard ratios (HR) with 95% CI per one unit increase in z score of the exposure; except for metabolic disturbance and type 2 diabetes as binary exposures. Weighted for likelihood of being tested for COVID-19 by Inverse Probability Weighting

Table S3b. Association between FMI z-score and COVID-19 outcomes with sequential adjustments in the UK Biobank study

Model and adjustment	Unweighted HR(95% CI)	Weighted HR(95% CI)
COVID-19 test		
Model 1=Age and gender	1.26 (1.16 to 1.37)	1.17 (1.07 to 1.28)
Model 2=Model 1+demographics	1.19 (1.09 to 1.29)	1.14 (1.04 to 1.24)
Model 3=Model 2+behavioural risks	1.17 (1.08 to 1.28)	1.13 (1.03 to 1.24)
Model 4=Model 3+Non-obesity-related morbidity	1.15 (1.06 to 1.25)	1.12 (1.03 to 1.23)
Model 5=Model 4+obesity-related morbidity	1.11 (1.01 to 1.21)	1.10 (1.00 to 1.20)
COVID-19 hospital admission		
Model 1=Age and gender	1.47 (1.28 to 1.70)	1.41 (1.22 to 1.63)
Model 2=Model 1+demographics	1.37 (1.19 to 1.57)	1.35 (1.17 to 1.55)
Model 3=Model 2+behavioural risks	1.32 (1.15 to 1.52)	1.31 (1.13 to 1.51)
Model 4=Model 3+Non-obesity-related morbidity	1.27 (1.11 to 1.47)	1.28 (1.11 to 1.48)
Model 5=Model 4+obesity-related morbidity	1.21 (1.05 to 1.40)	1.23 (1.06 to 1.42)
COVID-19 ICU admission		
Model 1=Age and gender	1.21 (0.89 to 1.66)	1.21 (0.88 to 1.66)
Model 2=Model 1+demographics	1.16 (0.85 to 1.58)	1.18 (0.87 to 1.61)
Model 3=Model 2+behavioural risks	1.15 (0.84 to 1.57)	1.17 (0.86 to 1.60)
Model 4=Model 3+Non-obesity-related morbidity	1.12 (0.82 to 1.53)	1.16 (0.84 to 1.58)
Model 5=Model 4+obesity-related morbidity	1.04 (0.76 to 1.43)	1.08 (0.79 to 1.48)
COVID-19 death		
Model 1=Age and gender	1.42 (1.20 to 1.70)	1.35 (1.10 to 1.65)
Model 2=Model 1+demographics	1.34 (1.13 to 1.59)	1.29 (1.06 to 1.57)
Model 3=Model 2+behavioural risks	1.32 (1.11 to 1.57)	1.27 (1.04 to 1.55)
Model 4=Model 3+Non-obesity-related morbidity	1.28 (1.08 to 1.53)	1.25 (1.02 to 1.53)
Model 5=Model 4+obesity-related morbidity	1.21 (1.02 to 1.45)	1.19 (0.97 to 1.46)
Note: Estimates are hazard ratios (HR) with 95% CI per one increase one unit increase in z score of the exposure; except for metabolic disturbance and type 2 diabetes as binary exposures.<br>Weighted for likelihood of being tested for COVID-19 by Inverse Probability Weighting		

Table S3c. Association between SMMI z-score and COVID-19 outcomes with sequential adjustments in the UK Biobank study

Model and adjustment	Unweighted HR(95% CI)	Weighted HR(95% CI)
COVID-19 test		
Model 1=Age and gender	1.07 (0.98 to 1.16)	1.05 (0.96 to 1.14)
Model 2=Model 1+demographics	1.08 (1.00 to 1.17)	1.06 (0.97 to 1.15)
Model 3=Model 2+behavioural risks	1.07 (0.99 to 1.16)	1.05 (0.96 to 1.14)
Model 4=Model 3+Non-obesity-related morbidity	1.08 (0.99 to 1.17)	1.05 (0.97 to 1.14)
Model 5=Model 4+obesity-related morbidity	1.05 (0.96 to 1.13)	1.04 (0.95 to 1.13)
COVID-19 hospital admission		
Model 1=Age and gender	1.03 (0.90 to 1.18)	1.01 (0.87 to 1.16)
Model 2=Model 1+demographics	1.05 (0.92 to 1.20)	1.02 (0.89 to 1.17)
Model 3=Model 2+behavioural risks	1.05 (0.92 to 1.20)	1.02 (0.89 to 1.17)
Model 4=Model 3+Non-obesity-related morbidity	1.06 (0.93 to 1.21)	1.03 (0.90 to 1.18)
Model 5=Model 4+obesity-related morbidity	1.01 (0.89 to 1.15)	0.99 (0.87 to 1.14)
COVID-19 ICU admission		
Model 1=Age and gender	1.41 (1.05 to 1.90)	1.35 (1.03 to 1.78)
Model 2=Model 1+demographics	1.42 (1.06 to 1.89)	1.35 (1.04 to 1.76)
Model 3=Model 2+behavioural risks	1.43 (1.07 to 1.91)	1.36 (1.04 to 1.78)
Model 4=Model 3+Non-obesity-related morbidity	1.43 (1.07 to 1.90)	1.36 (1.04 to 1.77)
Model 5=Model 4+obesity-related morbidity	1.32 (0.99 to 1.76)	1.27 (0.98 to 1.65)
COVID-19 death		
Model 1=Age and gender	1.09 (0.93 to 1.29)	1.10 (0.91 to 1.33)
Model 2=Model 1+demographics	1.09 (0.93 to 1.29)	1.10 (0.91 to 1.33)
Model 3=Model 2+behavioural risks	1.10 (0.93 to 1.29)	1.11 (0.92 to 1.34)
Model 4=Model 3+Non-obesity-related morbidity	1.11 (0.94 to 1.30)	1.11 (0.92 to 1.34)
Model 5=Model 4+obesity-related morbidity	1.03 (0.88 to 1.22)	1.05 (0.87 to 1.26)

Note: Estimates are hazard ratios (HR) with 95% CI per one increase one unit increase in z score of the exposure; except for metabolic disturbance and type 2 diabetes as binary exposures.
Weighted for likelihood of being tested for COVID-19 by Inverse Probability Weighting

Table S3d. Association between WHR z-score and COVID-19 outcomes with sequential adjustments in the UK Biobank study

Model and adjustment	Unweighted HR(95% CI)	Weighted HR(95% CI)
COVID-19 test		
Model 1=Age and gender	1.50 (1.40 to 1.6)	1.65 (1.47 to 1.85)
Model 2=Model 1+demographics	1.39 (1.29 to 1.49)	1.51 (1.35 to 1.70)
Model 3=Model 2+behavioural risks	1.36 (1.26 to 1.46)	1.47 (1.31 to 1.65)
Model 4=Model 3+Non-obesity-related morbidity	1.32 (1.23 to 1.42)	1.43 (1.27 to 1.62)
Model 5=Model 4+obesity-related morbidity	1.21 (1.12 to 1.31)	1.29 (1.14 to 1.46)
COVID-19 hospital admission		
Model 1=Age and gender	1.77 (1.61 to 1.94)	1.62 (1.46 to 1.79)
Model 2=Model 1+demographics	1.67 (1.49 to 1.86)	1.54 (1.37 to 1.72)
Model 3=Model 2+behavioural risks	1.61 (1.44 to 1.80)	1.49 (1.33 to 1.67)
Model 4=Model 3+Non-obesity-related morbidity	1.54 (1.37 to 1.73)	1.44 (1.28 to 1.62)
Model 5=Model 4+obesity-related morbidity	1.36 (1.20 to 1.55)	1.31 (1.16 to 1.49)
COVID-19 ICU admission		
Model 1=Age and gender	1.84 (1.52 to 2.22)	1.84 (1.57 to 2.16)
Model 2=Model 1+demographics	1.78 (1.44 to 2.22)	1.80 (1.50 to 2.16)
Model 3=Model 2+behavioural risks	1.76 (1.40 to 2.21)	1.79 (1.47 to 2.17)
Model 4=Model 3+Non-obesity-related morbidity	1.71 (1.34 to 2.18)	1.75 (1.42 to 2.15)
Model 5=Model 4+obesity-related morbidity	1.43 (1.07 to 1.92)	1.48 (1.16 to 1.90)
COVID-19 death		
Model 1=Age and gender	1.78 (1.58 to 2.02)	1.73 (1.50 to 2.00)
Model 2=Model 1+demographics	1.68 (1.46 to 1.93)	1.65 (1.41 to 1.94)
Model 3=Model 2+behavioural risks	1.62 (1.40 to 1.88)	1.60 (1.35 to 1.90)
Model 4=Model 3+Non-obesity-related morbidity	1.57 (1.35 to 1.83)	1.56 (1.31 to 1.86)
Model 5=Model 4+obesity-related morbidity	1.37 (1.17 to 1.61)	1.39 (1.16 to 1.68)

Note: Estimates are hazard ratios (HR) with 95% CI per one increase one unit increase in z score of the exposure; except for metabolic disturbance and type 2 diabetes as binary exposures.
Weighted for likelihood of being tested for COVID-19 by Inverse Probability Weighting

Table S3e. Association between metabolic disturbance and COVID-19 outcomes with sequential adjustments in the UK Biobank study

Model and adjustment	Unweighted HR(95% CI)	Weighted HR(95% CI)
COVID-19 test		
Model 1=Age and gender	2.34 (2.09 to 2.62)	1.72 (1.51 to 1.94)
Model 2=Model 1+demographics	1.98 (1.77 to 2.23)	1.56 (1.37 to 1.77)
Model 3=Model 2+behavioural risks	1.90 (1.69 to 2.14)	1.51 (1.33 to 1.71)
Model 4=Model 3+Non-obesity-related morbidity	1.81 (1.61 to 2.04)	1.47 (1.29 to 1.68)
Model 5=Model 4+obesity-related morbidity	1.50 (1.32 to 1.69)	1.31 (1.15 to 1.50)
COVID-19 hospital admission		
Model 1=Age and gender	3.13 (2.62 to 3.73)	2.36 (1.96 to 2.85)
Model 2=Model 1+demographics	2.51 (2.09 to 3.01)	2.06 (1.70 to 2.50)
Model 3=Model 2+behavioural risks	2.37 (1.97 to 2.85)	1.98 (1.63 to 2.40)
Model 4=Model 3+Non-obesity-related morbidity	2.19 (1.82 to 2.64)	1.87 (1.53 to 2.27)
Model 5=Model 4+obesity-related morbidity	1.69 (1.39 to 2.04)	1.52 (1.24 to 1.85)
COVID-19 ICU admission		
Model 1=Age and gender	3.24 (2.13 to 4.92)	2.38 (1.55 to 3.66)
Model 2=Model 1+demographics	2.58 (1.67 to 3.97)	2.07 (1.33 to 3.21)
Model 3=Model 2+behavioural risks	2.50 (1.62 to 3.87)	2.03 (1.31 to 3.16)
Model 4=Model 3+Non-obesity-related morbidity	2.34 (1.50 to 3.63)	1.92 (1.22 to 3.01)
Model 5=Model 4+obesity-related morbidity	1.54 (0.99 to 2.41)	1.32 (0.83 to 2.10)
COVID-19 death		
Model 1=Age and gender	3.03 (2.44 to 3.75)	2.35 (1.85 to 2.99)
Model 2=Model 1+demographics	2.57 (2.06 to 3.20)	2.07 (1.62 to 2.65)
Model 3=Model 2+behavioural risks	2.42 (1.94 to 3.03)	1.95 (1.53 to 2.48)
Model 4=Model 3+Non-obesity-related morbidity	2.30 (1.84 to 2.88)	1.86 (1.45 to 2.38)
Model 5=Model 4+obesity-related morbidity	1.77 (1.41 to 2.22)	1.49 (1.16 to 1.91)

Note: Estimates are hazard ratios (HR) with 95% CI per one increase one unit increase in z score of the exposure; except for metabolic disturbance and type 2 diabetes as binary exposures.
Weighted for likelihood of being tested for COVID-19 by Inverse Probability Weighting

Table S3f. Association between type 2 diabetes and COVID-19 outcomes with sequential adjustments in the UK Biobank study

Model and adjustment	Unweighted HR(95% CI)	Weighted HR(95% CI)
COVID-19 test		
Model 1=Age and gender	2.44 (2.16 to 2.76)	1.72 (1.51 to 1.94)
Model 2=Model 1+demographics	2.05 (1.80 to 2.32)	1.56 (1.37 to 1.77)
Model 3=Model 2+behavioural risks	1.95 (1.72 to 2.22)	1.51 (1.33 to 1.71)
Model 4=Model 3+Non-obesity-related morbidity	1.86 (1.63 to 2.11)	1.47 (1.29 to 1.68)
Model 5=Model 4+obesity-related morbidity	1.51 (1.33 to 1.72)	1.31 (1.15 to 1.50)
COVID-19 hospital admission		
Model 1=Age and gender	2.92 (2.41 to 3.54)	2.18 (1.78 to 2.66)
Model 2=Model 1+demographics	2.30 (1.89 to 2.80)	1.86 (1.52 to 2.28)
Model 3=Model 2+behavioural risks	2.15 (1.76 to 2.63)	1.77 (1.44 to 2.17)
Model 4=Model 3+Non-obesity-related morbidity	1.99 (1.63 to 2.43)	1.67 (1.35 to 2.05)
Model 5=Model 4+obesity-related morbidity	1.51 (1.23 to 1.85)	1.34 (1.09 to 1.66)
COVID-19 ICU admission		
Model 1=Age and gender	2.75 (1.73 to 4.38)	2.03 (1.26 to 3.26)
Model 2=Model 1+demographics	2.15 (1.33 to 3.46)	1.71 (1.06 to 2.77)
Model 3=Model 2+behavioural risks	2.06 (1.27 to 3.34)	1.66 (1.02 to 2.70)
Model 4=Model 3+Non-obesity-related morbidity	1.92 (1.18 to 3.12)	1.57 (0.96 to 2.57)
Model 5=Model 4+obesity-related morbidity	1.24 (0.76 to 2.02)	1.06 (0.64 to 1.75)
COVID-19 death		
Model 1=Age and gender	3.17 (2.53 to 3.97)	2.37 (1.84 to 3.05)
Model 2=Model 1+demographics	2.65 (2.11 to 3.35)	2.07 (1.60 to 2.68)
Model 3=Model 2+behavioural risks	2.50 (1.98 to 3.16)	1.95 (1.51 to 2.52)
Model 4=Model 3+Non-obesity-related morbidity	2.38 (1.88 to 3.01)	1.86 (1.44 to 2.41)
Model 5=Model 4+obesity-related morbidity	1.80 (1.42 to 2.28)	1.47 (1.13 to 1.91)

Note: Estimates are hazard ratios (HR) with 95% CI per one increase one unit increase in z score of the exposure; except for metabolic disturbance and type 2 diabetes as binary exposures.
Weighted for likelihood of being tested for COVID-19 by Inverse Probability Weighting

Table S4. Associations of adiposity markers with COVID-19 outcomes with correction for regression dilution bias in the UK Biobank study (n=435,504)

	**COVID-19 positive test (N=5566)**	**COVID-19 hospital admission (N= 567)**	**COVID-19 ICU admission (N= 107)**	**COVID-19 death (N=366)**
**Exposures**	HR (95% CI)	HR (95% CI)	HR (95% CI)	HR (95% CI)
BMI	1.13(1.07-1.19)	1.21(1.11-1.31)	1.38(1.20-1.58)	1.23(1.09-1.39)
FMI	1.10(1.00-1.20)	1.23(1.06-1.42)	1.08(0.79-1.48)	1.19(0.97-1.46)
SMMI	1.04(0.95-1.13)	0.99(0.87-1.14)	1.27(0.98-1.65)	1.05(0.87-1.26)
WHR	1.15(1.07-1.25)	1.31(1.16-1.49)	1.48(1.16-1.90)	1.39(1.16-1.68)
Metabolic disturbance	1.29(1.14-1.46)	1.52(1.24-1.85)	1.32(0.83-2.10)	1.49(1.16-1.91)
Type 2 diabetes	1.31(1.15-1.50)	1.34(1.09-1.66)	1.06(0.64-1.75)	1.47(1.13-1.91)

Note: Estimates are hazard ratios (HR) with 95% CI per one increase in z score of the exposure except for metabolic disturbance and type 2 diabetes as binary exposure.
Adjusted for demographic factors (age, gender, Townsend index, education), behavioural risk factors (smoking, alcohol consumption, diet and physical activity), non-obesity-related morbidity (COPD, asthma, autoimmune rheumatological conditions, ulcerative colitis and Crohn's disease) and obesity-related morbidity (hypertension, CVD, GORD and sleep apnoea)

Figure S1. DAG for the association between BMI, body fat distribution, and conditions caused by metabolic disturbance and incidence of severe COVID-19 outcomes

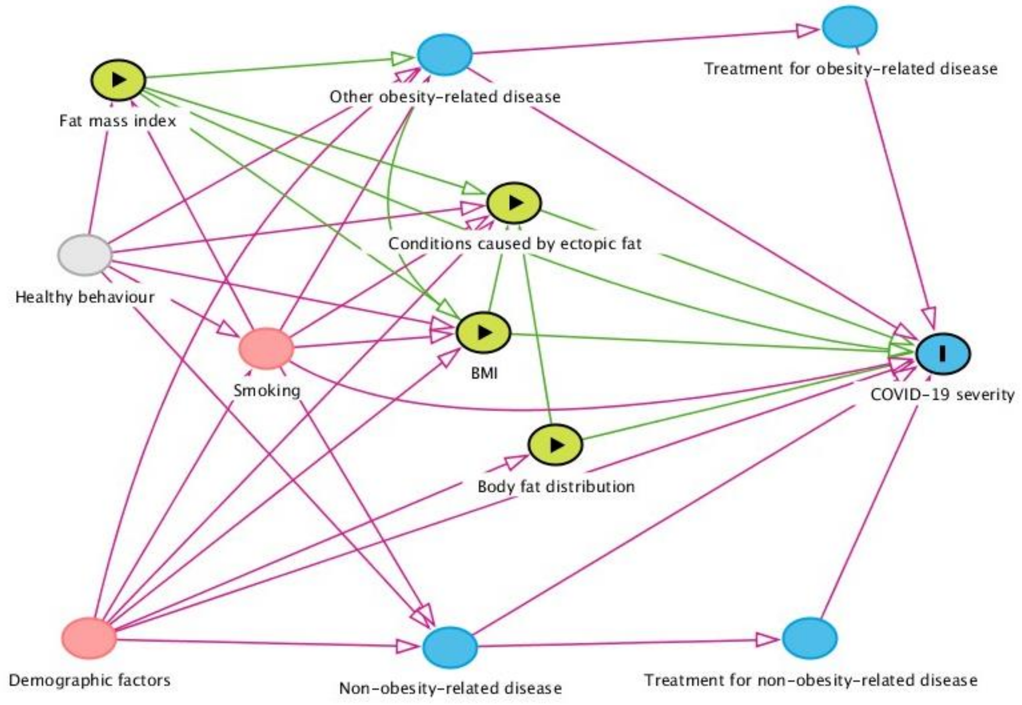

Note: Key- exposures of interest are shown in green, the outcome and direct ancestors of the outcome in blue, unmeasured variables in grey, and other variables in red. Green paths represent the causal paths of interest, while red paths represent biasing paths.

Figure S2a. Restricted cubic spline models for BMI z scores showing the hazard ratio for its association with COVID-19 outcomes

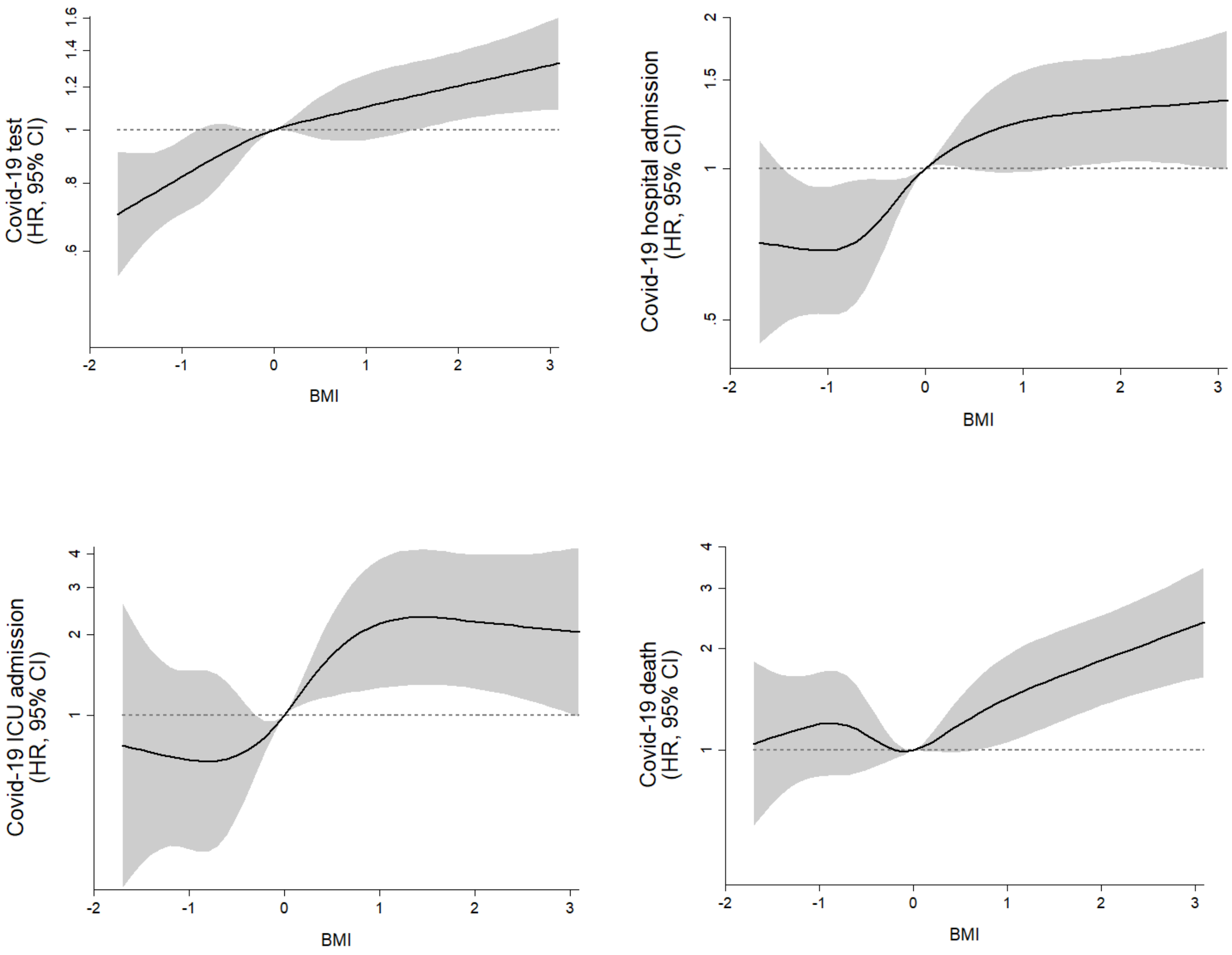

Note: The y axis is on a logarithmic scale.

Figure S2b. Restricted cubic spline models for FMI z scores showing the hazard ratio for its association with COVID-19 outcomes

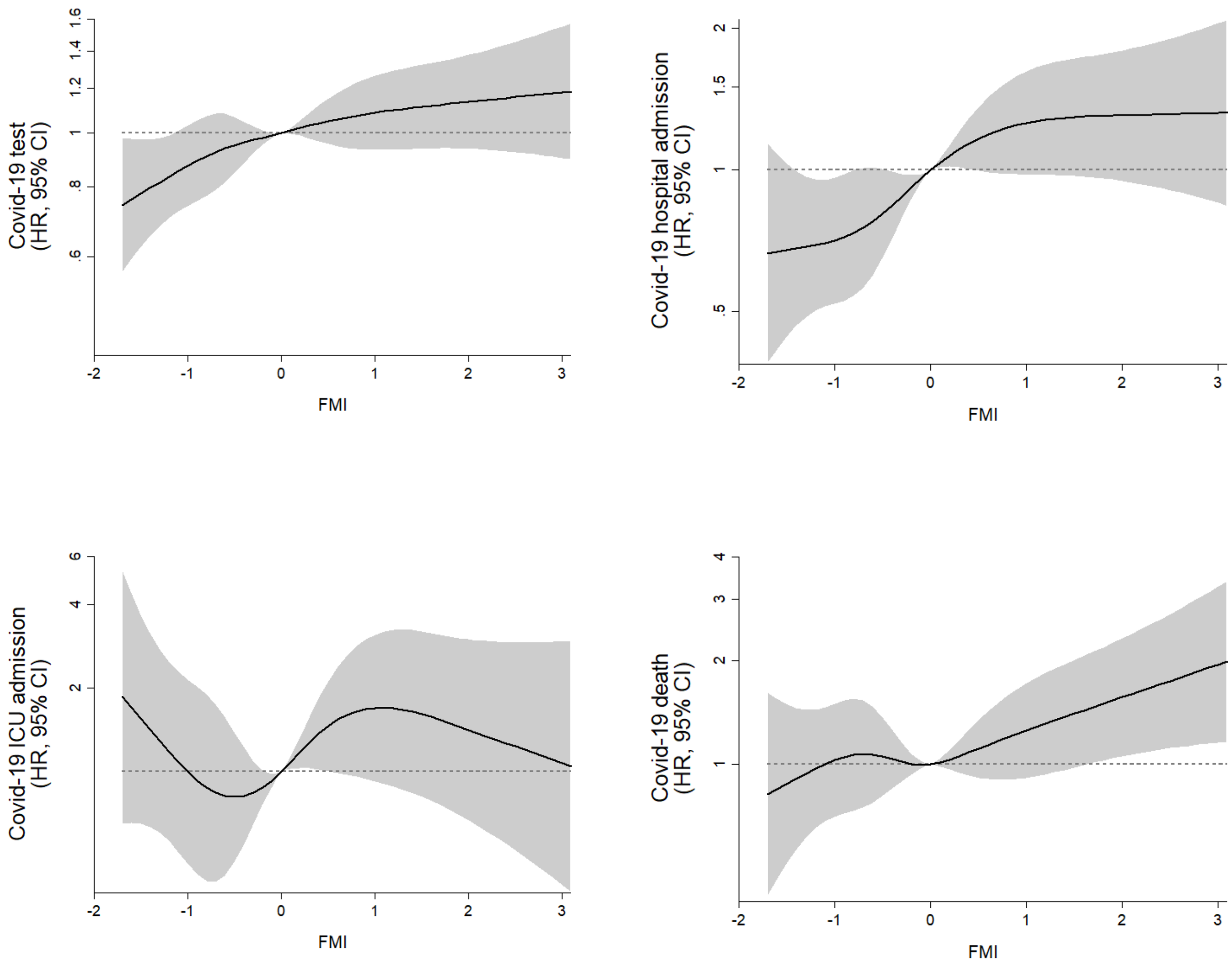

Note: The y axis is on a logarithmic scale.

Figure S2c. Restricted cubic spline models for SMMI z scores showing the hazard ratio for its association with COVID-19 outcomes

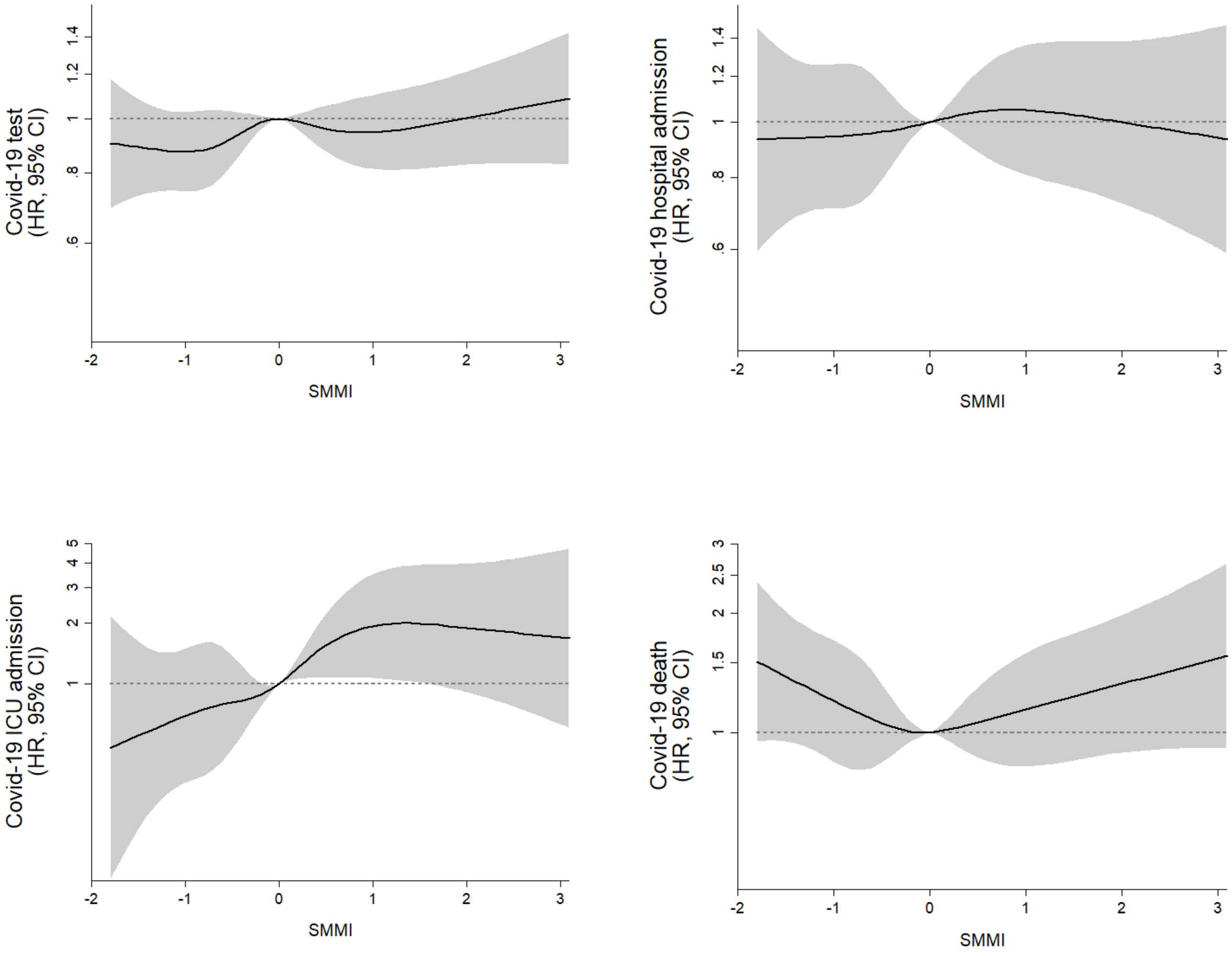

Note: The y axis is on a logarithmic scale.

Figure S2d. Restricted cubic spline models for WHR z scores showing the hazard ratio for its association with COVID-19 outcomes

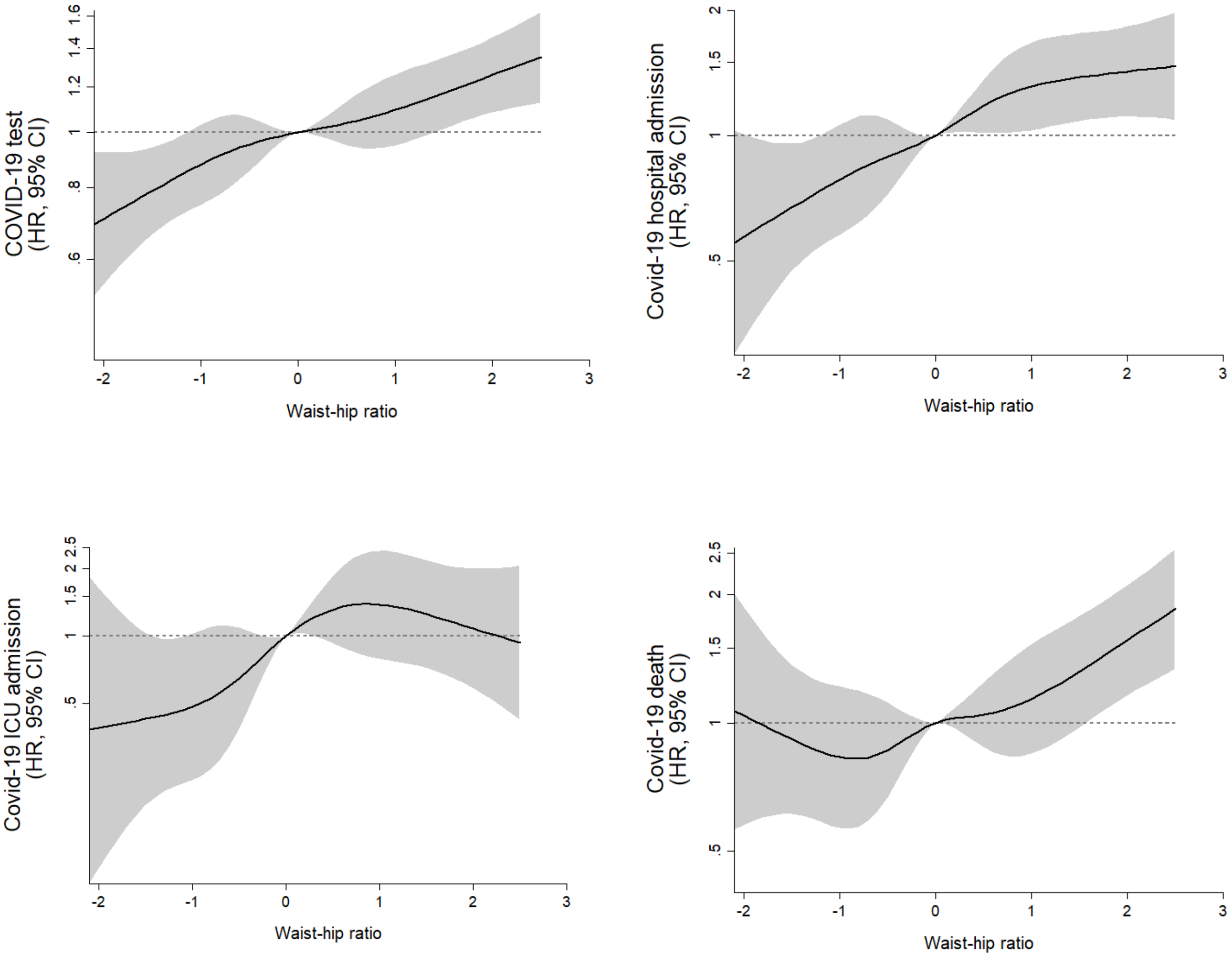

Note: The y axis is on a logarithmic scale.

Figure S3a. The causal effect estimates on COVID-19 test across different MR methods

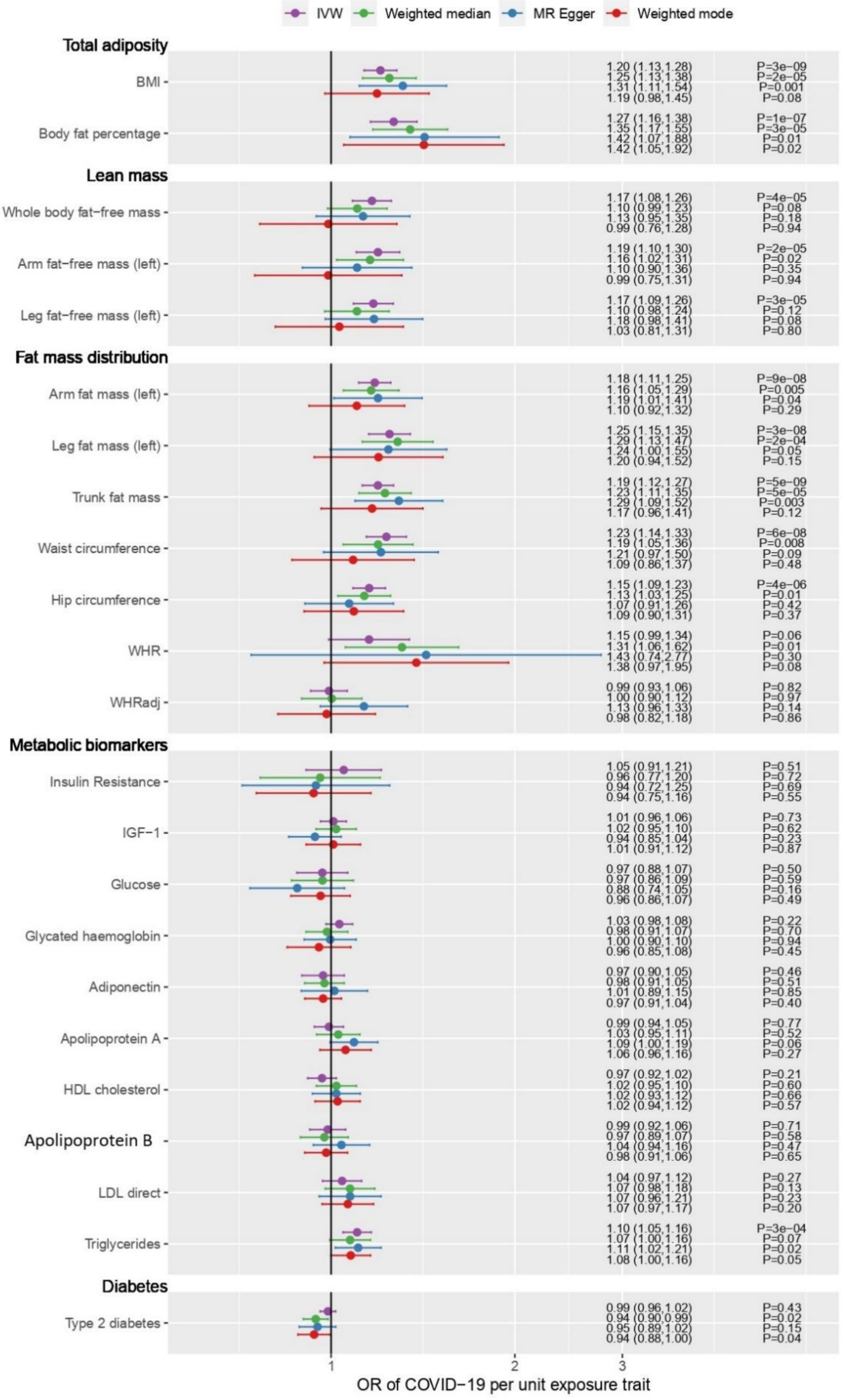

Note: For quantitative traits, the units are OR per SD; for binary traits, the units are OR per log(OR)

Figure S3b. The causal effect estimates on COVID-19 hospital admission across different MR methods

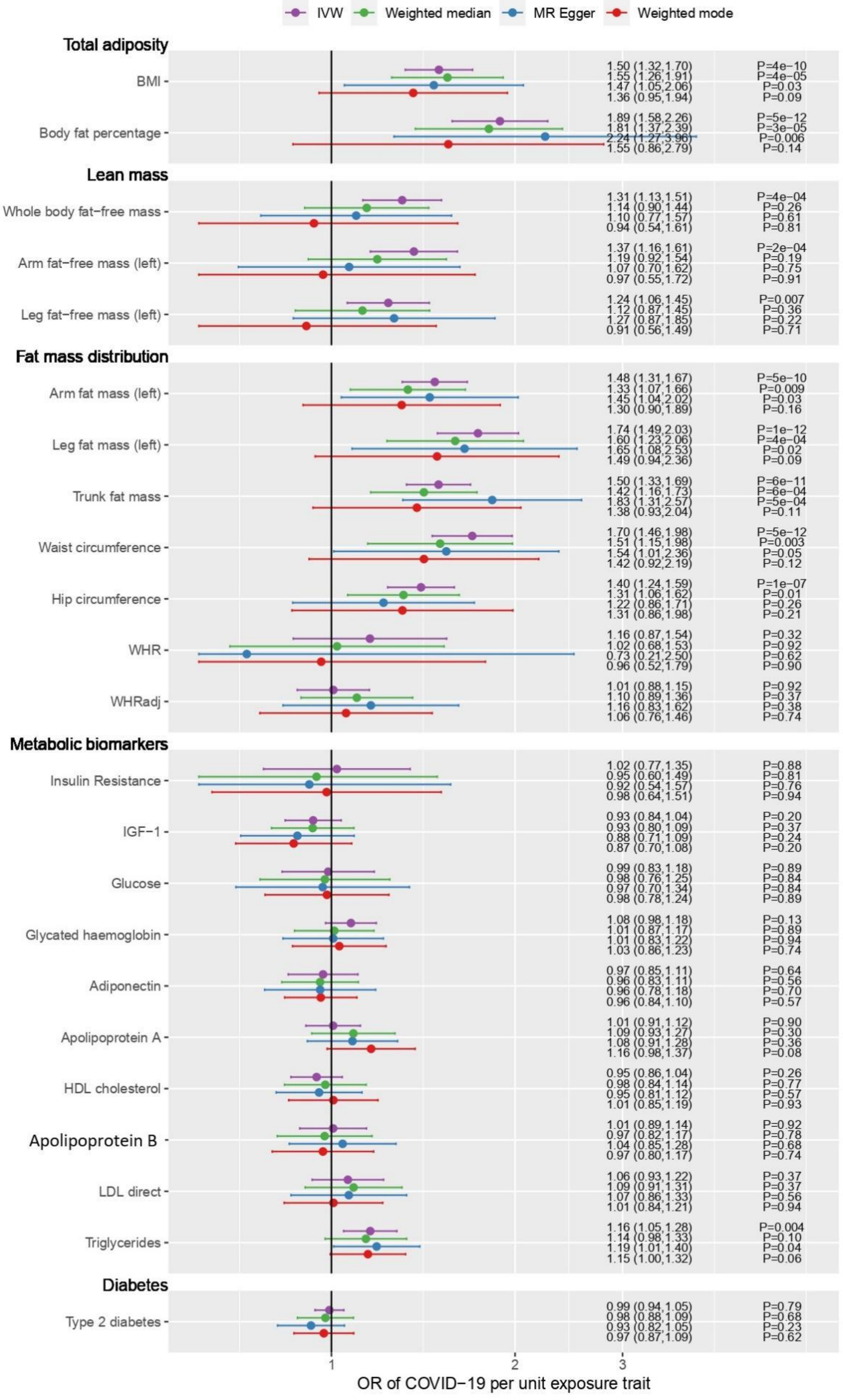

Note: For quantitative traits, the units are OR per SD; for binary traits, the units are OR per log(OR)

Figure S3c. The causal effect estimates on very severe confirmed COVID-19 across different MR methods

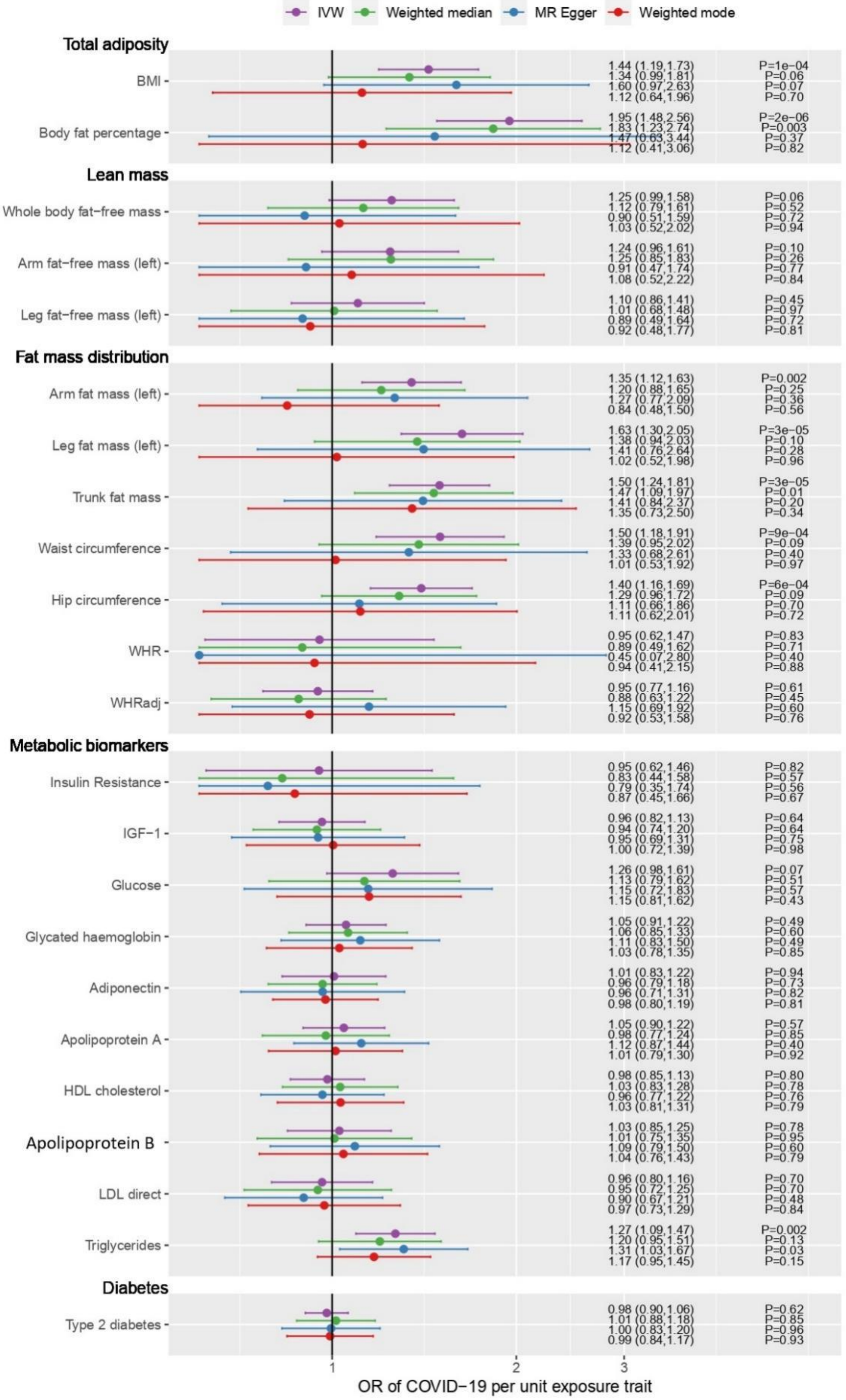

Note: For quantitative traits, the units are OR per SD; for binary traits, the units are OR per log(OR)

Figure S4a. Effect modification of the associations between BMI and COVID-19 outcomes by age, gender, ethnicity, and cardiovascular disease

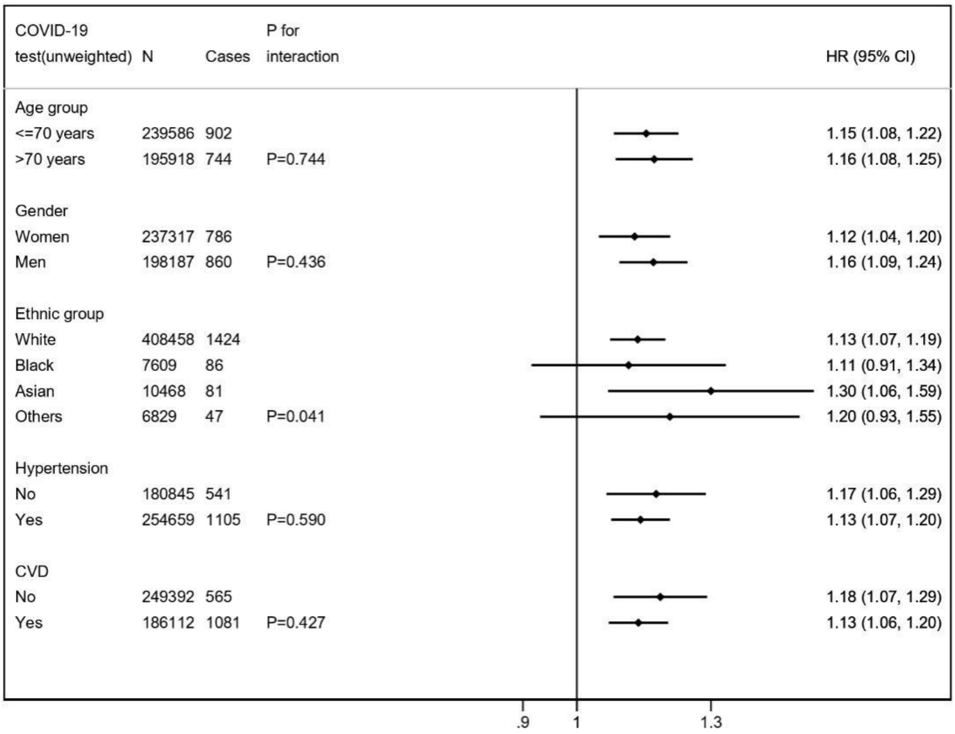

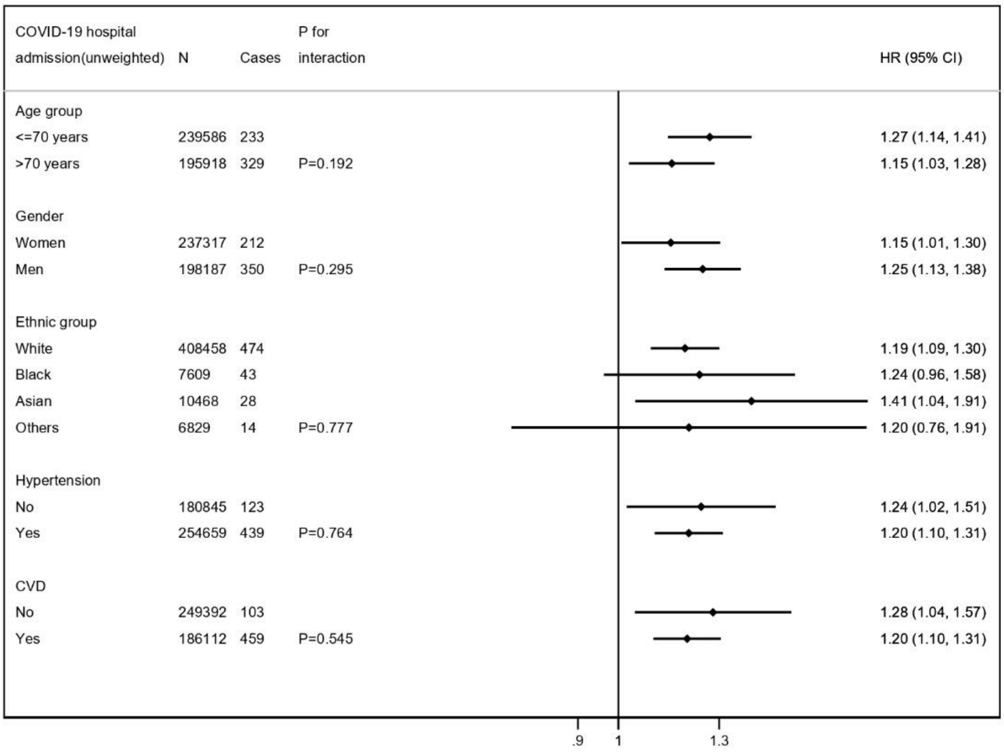

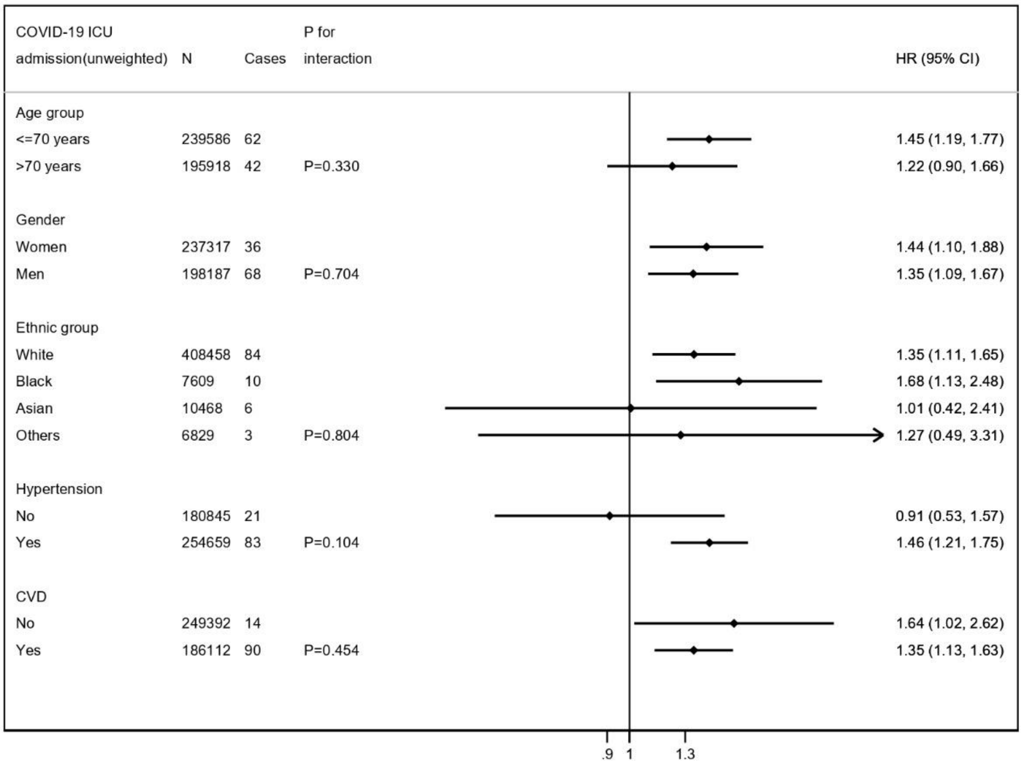
COVID-19 ICU admission(unweighted)
N
Cases
P for interaction
HR (95% CI)
Age group
<=70 years 239586 62 1.45 (1.19, 1.77)
>70 years 195918 42 P=0.330 1.22 (0.90, 1.66)
Gender
Women 237317 36 1.44 (1.10, 1.88)
Men 198187 68 P=0.704 1.35 (1.09, 1.67)
Ethnic group
White 408458 84 1.35 (1.11, 1.65)
Black 7609 10 1.68 (1.13, 2.48)
Asian 10468 6 1.01 (0.42, 2.41)
Others 6829 3 P=0.804 1.27 (0.49, 3.31)
Hypertension
No 180845 21 0.91 (0.53, 1.57)
Yes 254659 83 P=0.104 1.46 (1.21, 1.75)
CVD
No 249392 14 1.64 (1.02, 2.62)
Yes 186112 90 P=0.454 1.35 (1.13, 1.63)
.9
1
1.3

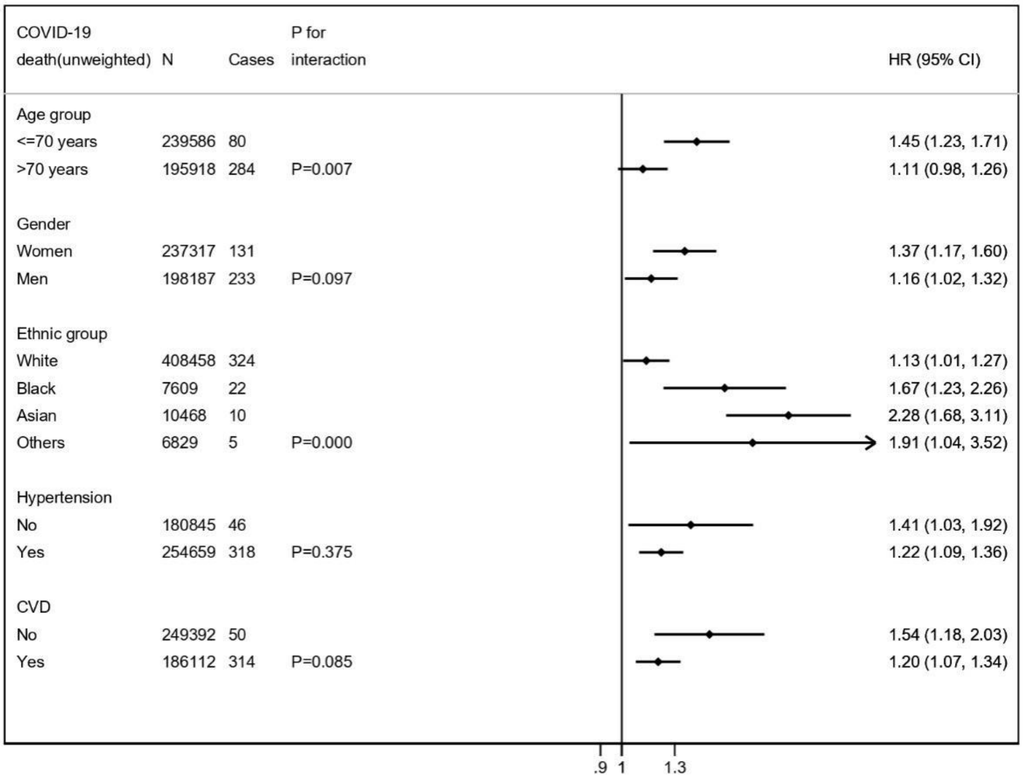
COVID-19 death(unweighted)
N
Cases
P for interaction
HR (95% CI)
Age group
<=70 years 239586 80 1.45 (1.23, 1.71)
>70 years 195918 284 P=0.007 1.11 (0.98, 1.26)
Gender
Women 237317 131 1.37 (1.17, 1.60)
Men 198187 233 P=0.097 1.16 (1.02, 1.32)
Ethnic group
White 408458 324 1.13 (1.01, 1.27)
Black 7609 22 1.67 (1.23, 2.26)
Asian 10468 10 2.28 (1.68, 3.11)
Others 6829 5 P=0.000 1.91 (1.04, 3.52)
Hypertension
No 180845 46 1.41 (1.03, 1.92)
Yes 254659 318 P=0.375 1.22 (1.09, 1.36)
CVD
No 249392 50 1.54 (1.18, 2.03)
Yes 186112 314 P=0.085 1.20 (1.07, 1.34)
.9
1
1.3

Figure S4b. Effect modification of the associations between FMI and COVID-19 outcomes by age, gender, ethnicity, and cardiovascular disease

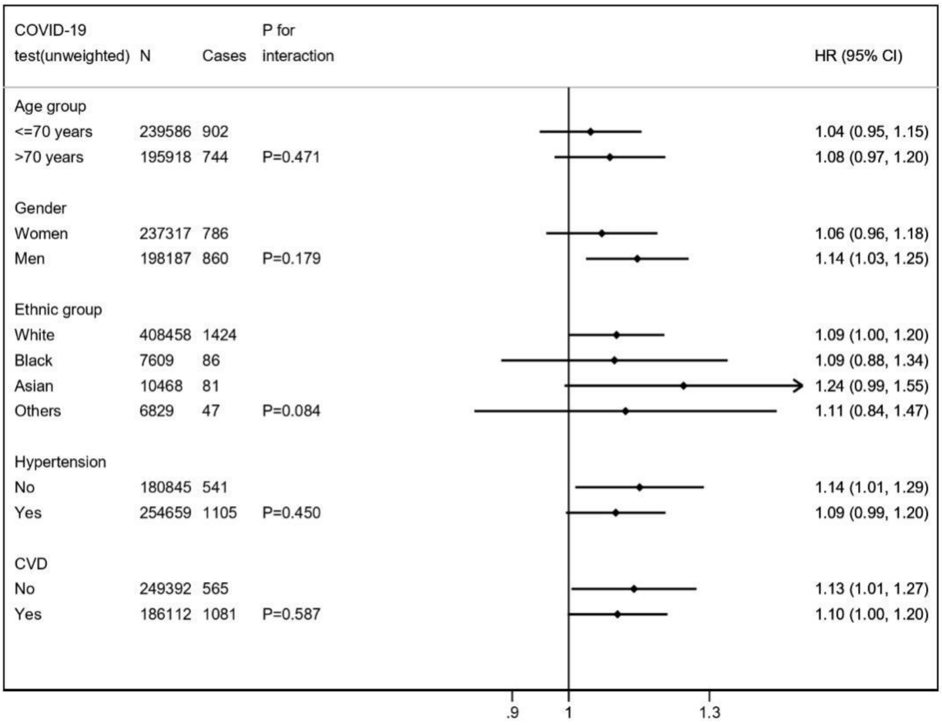

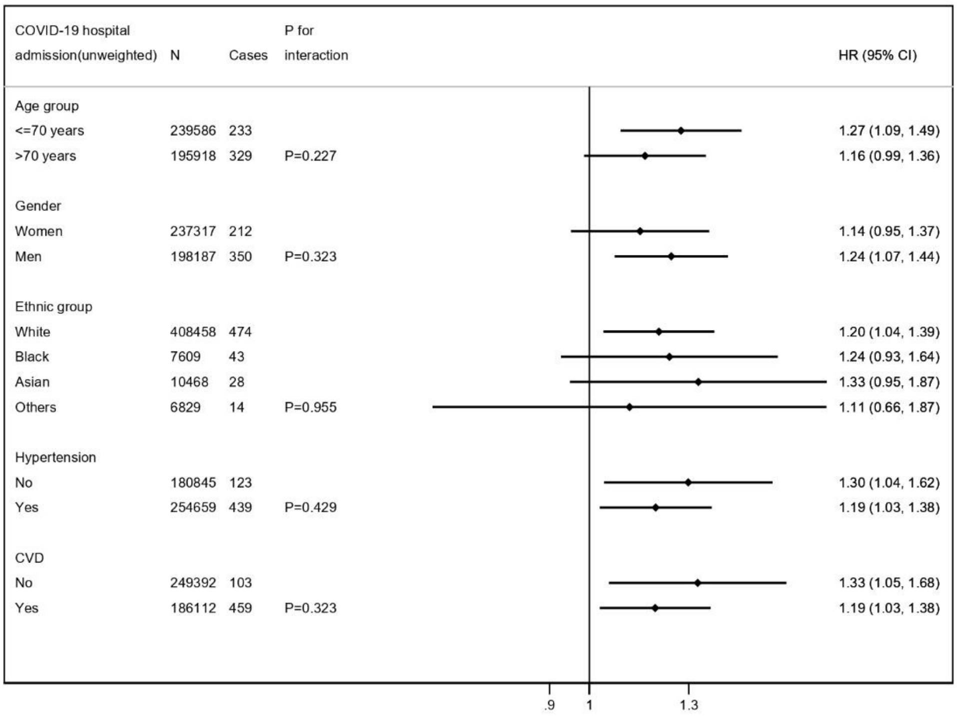

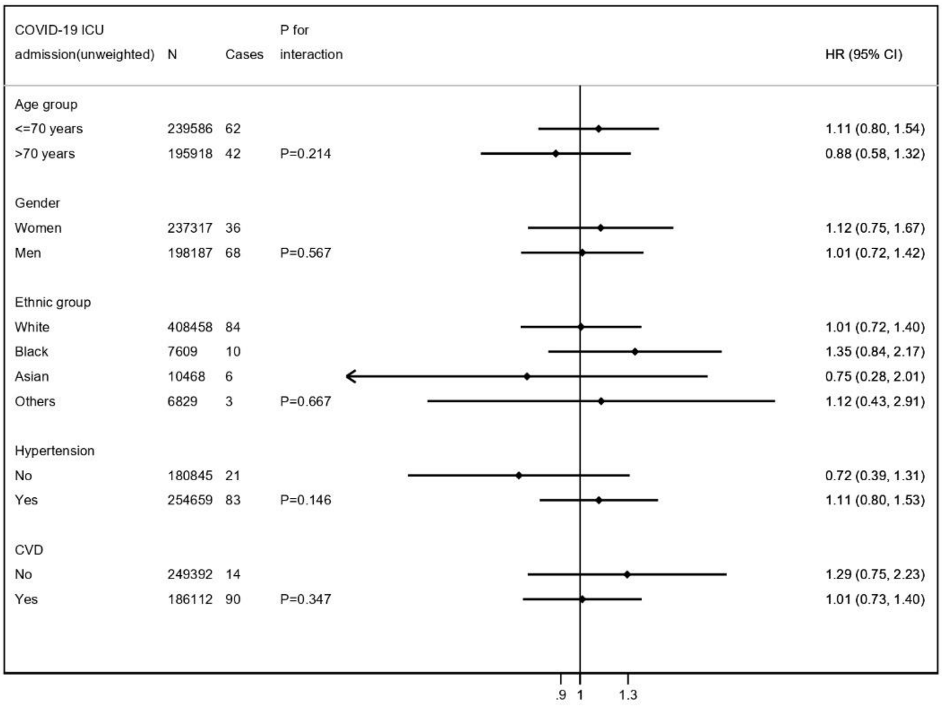
COVID-19 ICU admission(unweighted)
N
Cases
P for interaction
HR (95% CI)
Age group
<=70 years 239586 62 1.11 (0.80, 1.54)
>70 years 195918 42 P=0.214 0.88 (0.58, 1.32)
Gender
Women 237317 36 1.12 (0.75, 1.67)
Men 198187 68 P=0.567 1.01 (0.72, 1.42)
Ethnic group
White 408458 84 1.01 (0.72, 1.40)
Black 7609 10 1.35 (0.84, 2.17)
Asian 10468 6 0.75 (0.28, 2.01)
Others 6829 3 P=0.667 1.12 (0.43, 2.91)
Hypertension
No 180845 21 0.72 (0.39, 1.31)
Yes 254659 83 P=0.146 1.11 (0.80, 1.53)
CVD
No 249392 14 1.29 (0.75, 2.23)
Yes 186112 90 P=0.347 1.01 (0.73, 1.40)
.9
1
1.3

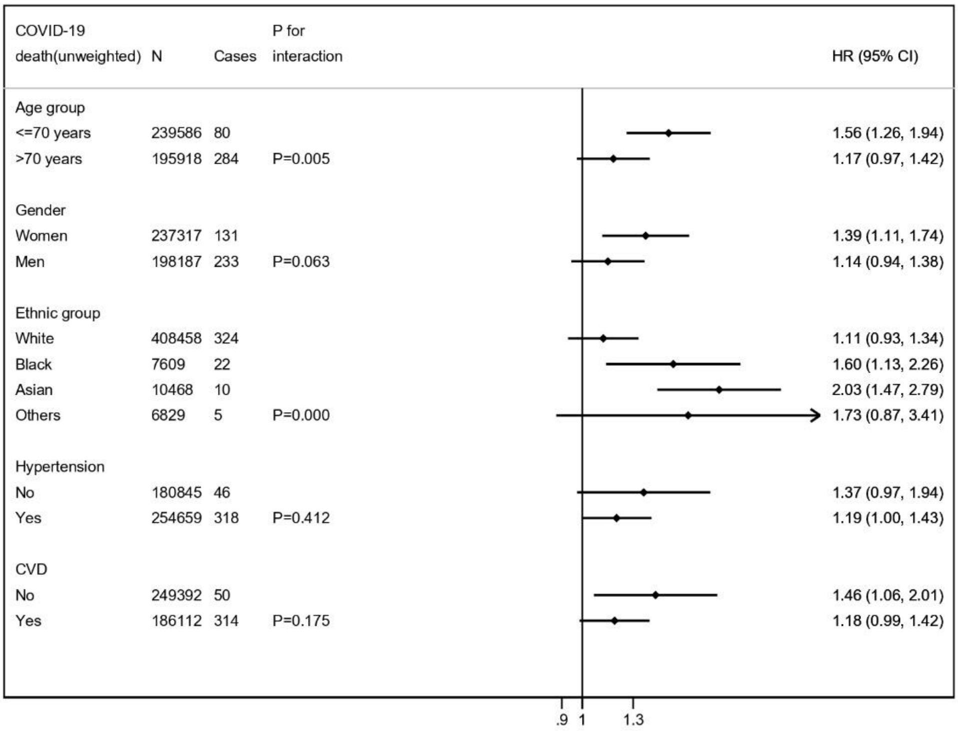
COVID-19 death(unweighted)
N
Cases
P for interaction
HR (95% CI)
Age group
<=70 years 239586 80 1.56 (1.26, 1.94)
>70 years 195918 284 P=0.005 1.17 (0.97, 1.42)
Gender
Women 237317 131 1.39 (1.11, 1.74)
Men 198187 233 P=0.063 1.14 (0.94, 1.38)
Ethnic group
White 408458 324 1.11 (0.93, 1.34)
Black 7609 22 1.60 (1.13, 2.26)
Asian 10468 10 2.03 (1.47, 2.79)
Others 6829 5 P=0.000 1.73 (0.87, 3.41)
Hypertension
No 180845 46 1.37 (0.97, 1.94)
Yes 254659 318 P=0.412 1.19 (1.00, 1.43)
CVD
No 249392 50 1.46 (1.06, 2.01)
Yes 186112 314 P=0.175 1.18 (0.99, 1.42)
.9
1
1.3

Figure S4c. Effect modification of the associations between WHR and COVID-19 outcomes by age, gender, ethnicity, and cardiovascular disease

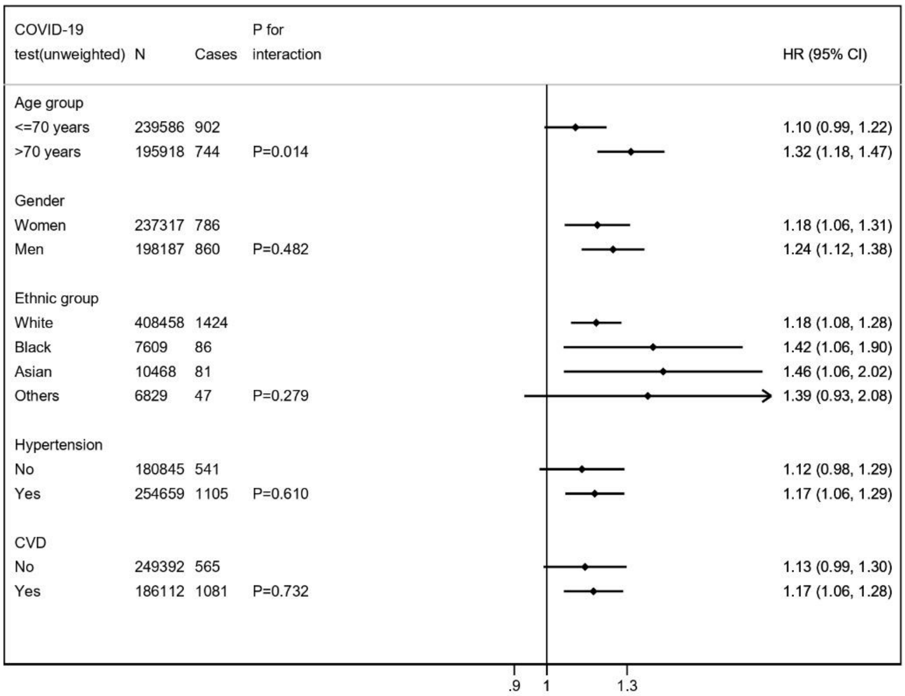

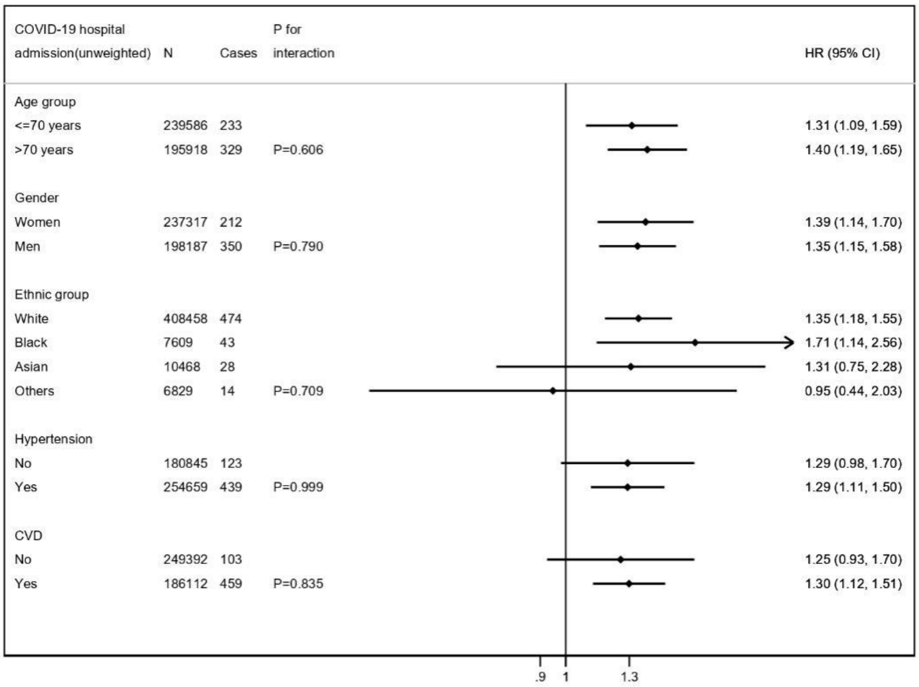

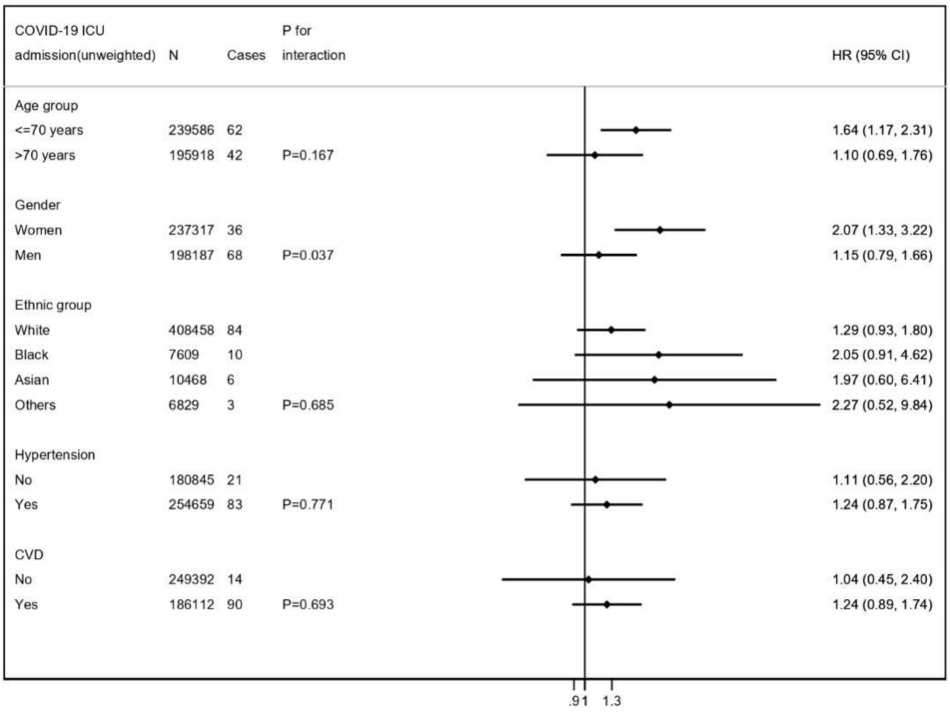
COVID-19 ICU admission(unweighted)
N
Cases
P for interaction
HR (95% CI)
Age group
<=70 years 239586 62 1.64 (1.17, 2.31)
>70 years 195918 42 P=0.167 1.10 (0.69, 1.76)
Gender
Women 237317 36 2.07 (1.33, 3.22)
Men 198187 68 P=0.037 1.15 (0.79, 1.66)
Ethnic group
White 408458 84 1.29 (0.93, 1.80)
Black 7609 10 2.05 (0.91, 4.62)
Asian 10468 6 1.97 (0.60, 6.41)
Others 6829 3 P=0.685 2.27 (0.52, 9.84)
Hypertension
No 180845 21 1.11 (0.56, 2.20)
Yes 254659 83 P=0.771 1.24 (0.87, 1.75)
CVD
No 249392 14 1.04 (0.45, 2.40)
Yes 186112 90 P=0.693 1.24 (0.89, 1.74)
.9
1
1.3

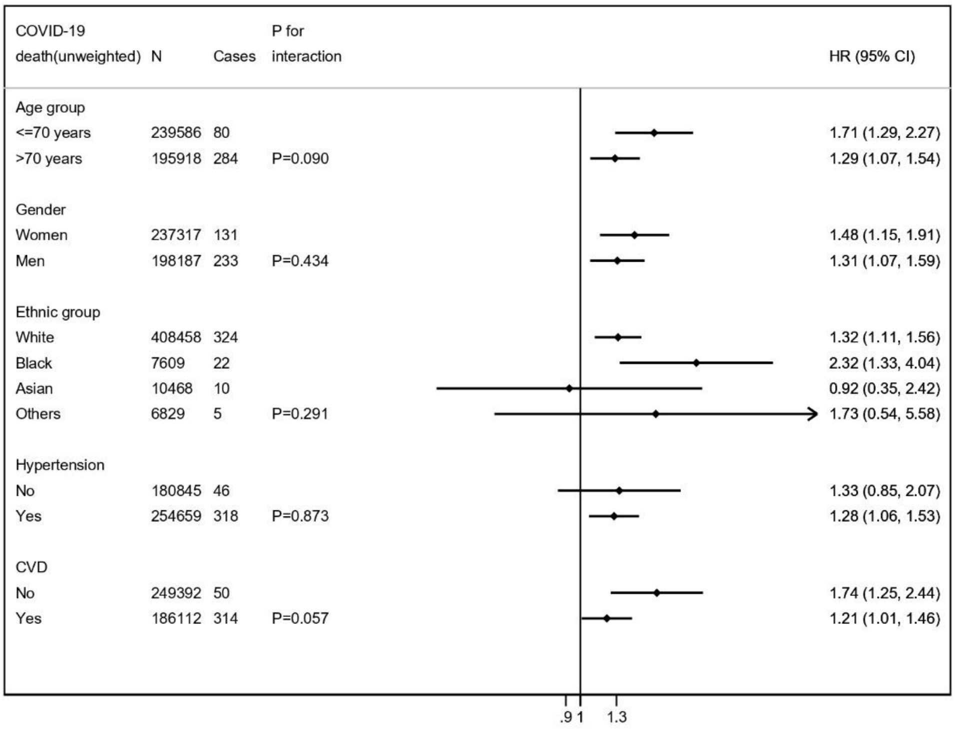
COVID-19 death(unweighted)
N
Cases
P for interaction
HR (95% CI)
Age group
<=70 years 239586 80 1.71 (1.29, 2.27)
>70 years 195918 284 P=0.090 1.29 (1.07, 1.54)
Gender
Women 237317 131 1.48 (1.15, 1.91)
Men 198187 233 P=0.434 1.31 (1.07, 1.59)
Ethnic group
White 408458 324 1.32 (1.11, 1.56)
Black 7609 22 2.32 (1.33, 4.04)
Asian 10468 10 0.92 (0.35, 2.42)
Others 6829 5 P=0.291 1.73 (0.54, 5.58)
Hypertension
No 180845 46 1.33 (0.85, 2.07)
Yes 254659 318 P=0.873 1.28 (1.06, 1.53)
CVD
No 249392 50 1.74 (1.25, 2.44)
Yes 186112 314 P=0.057 1.21 (1.01, 1.46)
.9
1
1.3

Figure S4d. Effect modification of the associations between metabolic disturbance and COVID-19 outcomes by age, gender, ethnicity, and cardiovascular disease

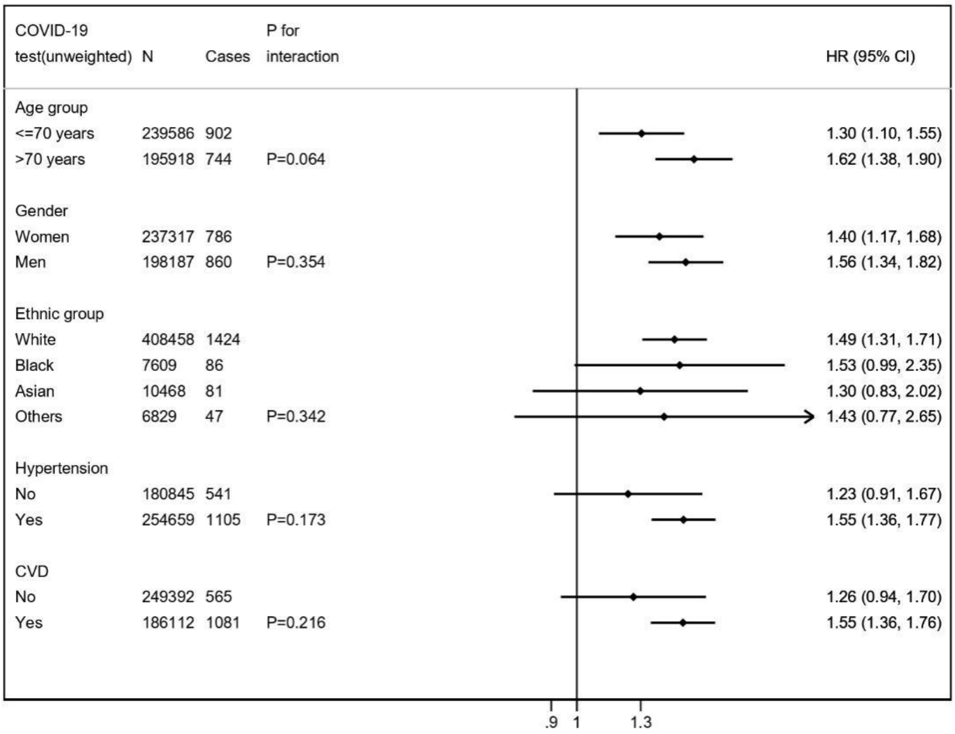

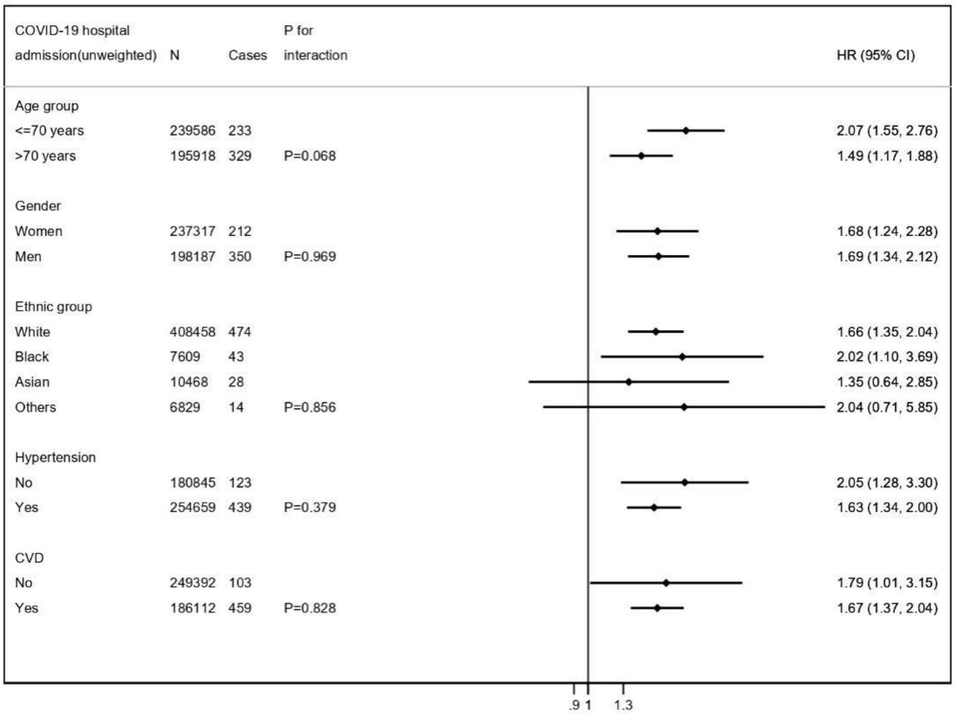

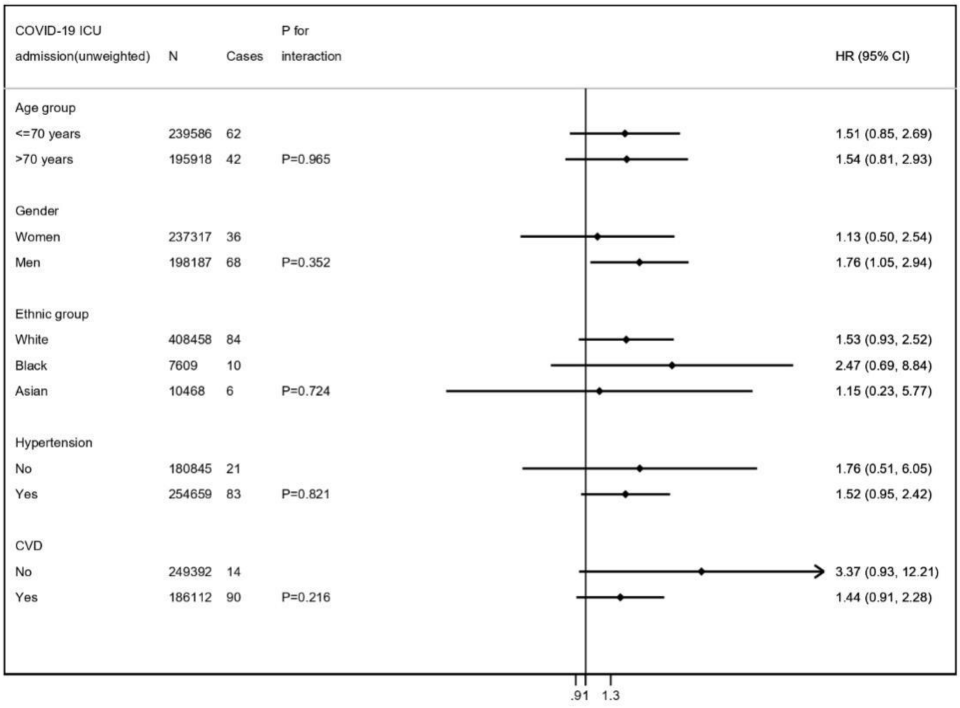

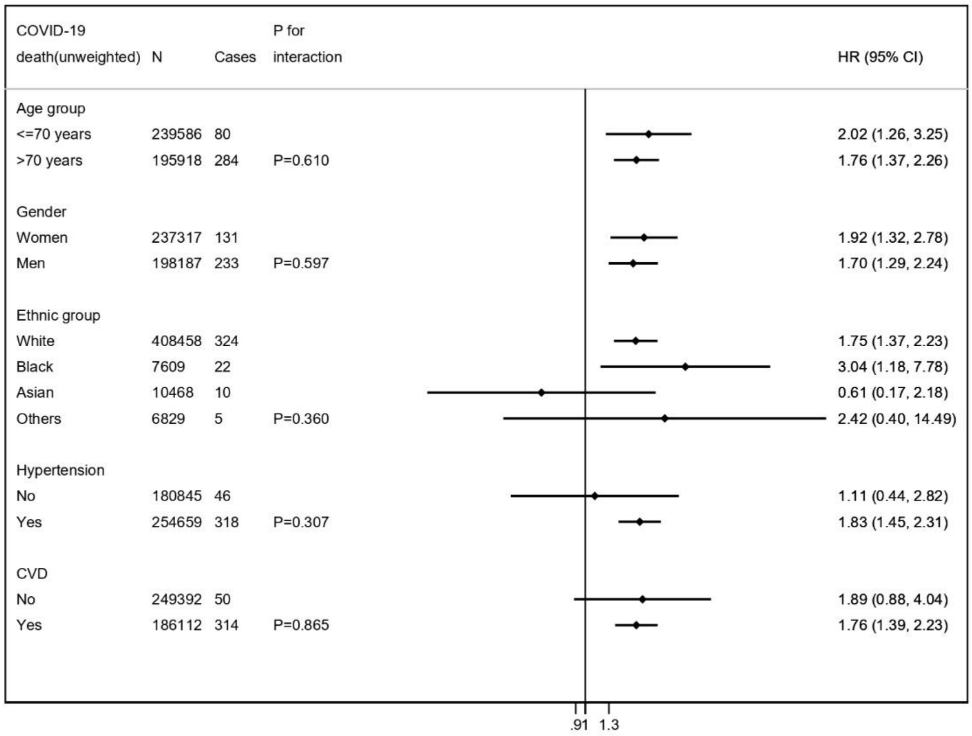

Note: For metabolic disturbance, we did not present people with an ethnicity defined as "others" in analyses of COVID-19 ICU admission because there were only three people with this outcome.

Figure S4e. Effect modification of the associations between type 2 diabetes and COVID-19 outcomes by age, gender, ethnicity, and cardiovascular disease

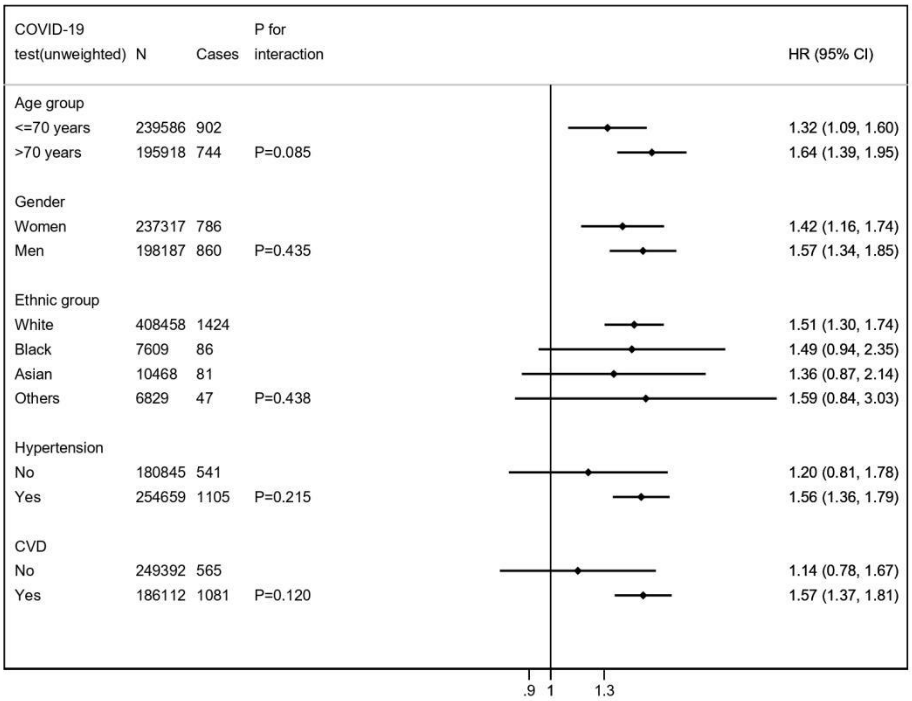

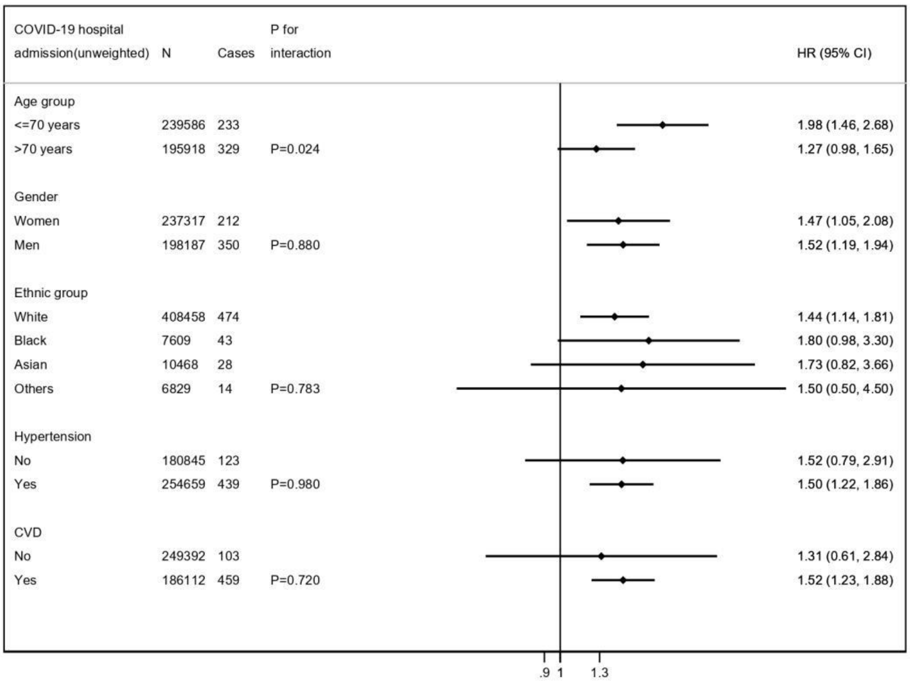

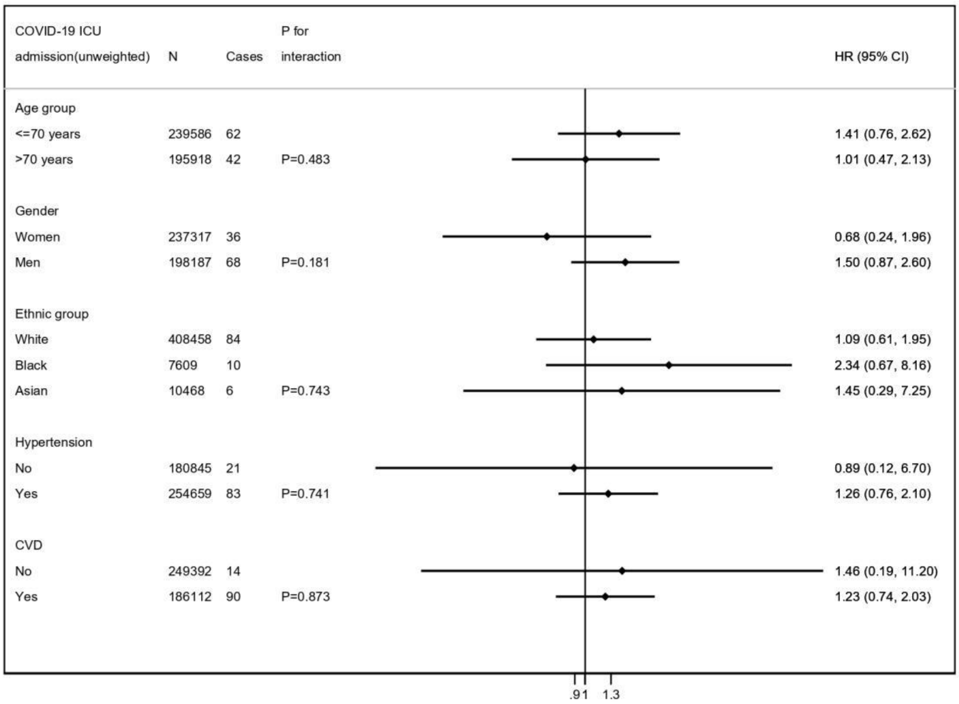

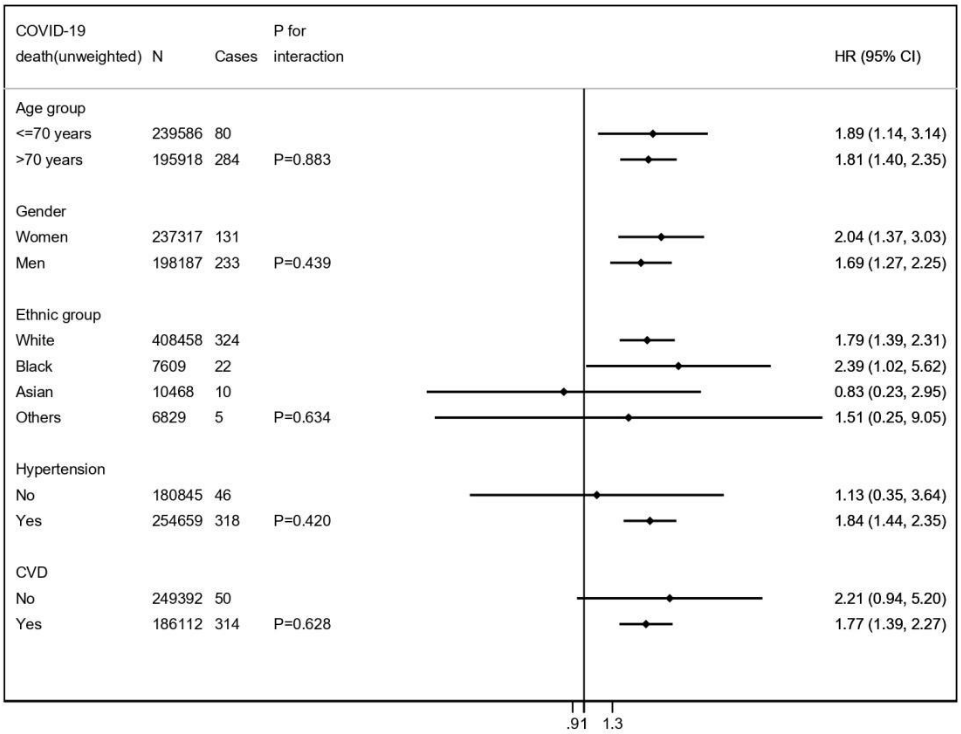

Note: For type 2 diabetes, we did not present people with an ethnicity defined as “others” in analyses of COVID-19 ICU admission because there were only three people with this outcome.

Figure S5. Multivariable MR analysis assessing the causal effect estimates of BMI with individually adjusting for the risk factors listed on the y-axis.

The causal effects of BMI are shown for Covid-19 positive (left) and hospitalised Covid-19 (right).

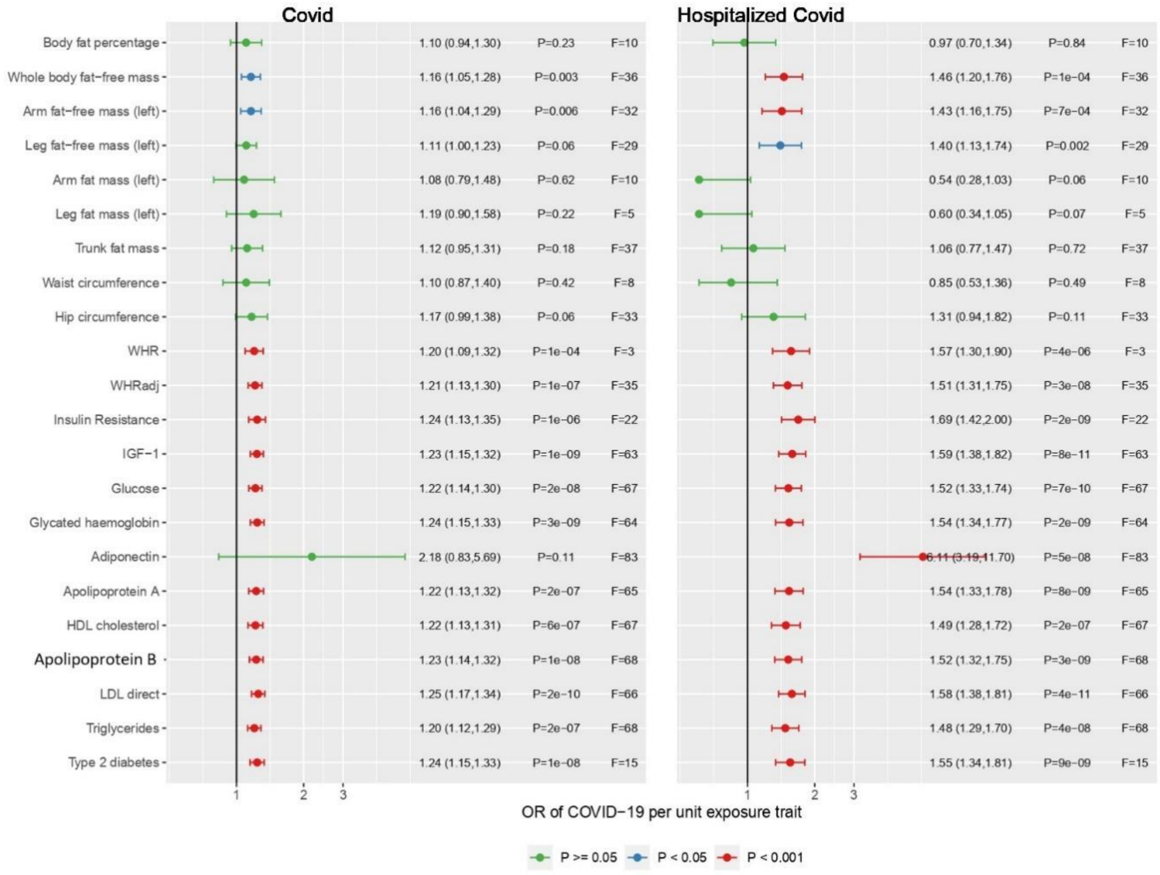

Note: For quantitative traits, the units are OR per SD; for binary traits, the units are OR per log(OR)

Figure S6. Multivariate MR analysis assessing the comparative causal role of each individual risk factor (listed on the y-axis) on the risk of COVID-19 conditional on body mass index

The comparative effects are shown for Covid-19 positive (left) and hospitalised Covid-19 (right). The comparative effect estimates from MVMR are further compared to those from univariable MR.

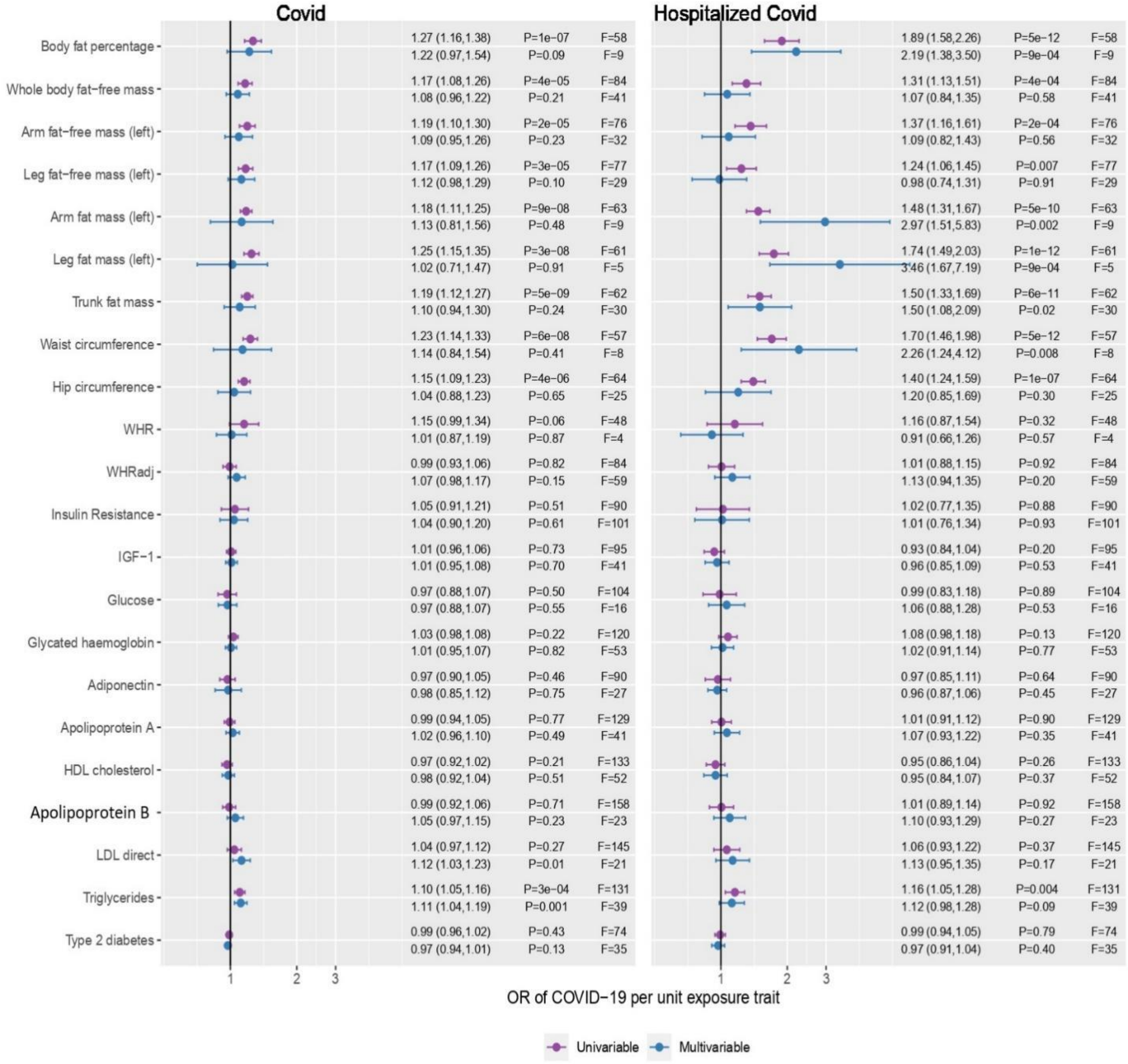

Note: For quantitative traits, the units are OR per SD; for binary traits, the units are OR per log(OR)
